# A barcoding pipeline for mosquito surveillance in Nepal, a biodiverse dengue-endemic country

**DOI:** 10.1186/s13071-022-05255-1

**Published:** 2022-04-24

**Authors:** Juliane Hartke, Friederike Reuss, Isabelle Marie Kramer, Axel Magdeburg, Isra Deblauwe, Reshma Tuladhar, Ishan Gautam, Meghnath Dhimal, Ruth Müller

**Affiliations:** 1 Unit Entomology, Institute of Tropical Medicine, Nationalestraat 155, 2000 Antwerp, Belgium; 2 Senckenberg Biodiversity and Climate Research Centre, Georg-Voigt-Str. 14-16, 60325 Frankfurt am Main, Germany; 3 Institute of Occupational, Social and Environmental Medicine, Goethe University, Theodor-Stern-Kai 7, 60590 Frankfurt am Main, Germany; 4 Central Department of Microbiology, Tribhuvan University, Kathmandu, Nepal; 5 Natural History Museum, Tribhuvan University, Kathmandu, Nepal; 6 Nepal Health Research Council, Ramshah Path, Kathmandu, 44600 Nepal; 7 Present Address: Institute of Organismic and Molecular Evolution, Johannes Gutenberg University Mainz, 55128 Mainz, Germany

**Keywords:** Species identification, VBD, Surveillance, Webinar, Low-cost, *Aedes*, *Anopheles*

## Abstract

**Background:**

Vector-borne diseases are on the rise on a global scale, which is anticipated to further accelerate because of anthropogenic climate change. Resource-limited regions are especially hard hit by this increment with the currently implemented surveillance programs being inadequate for the observed expansion of potential vector species. Cost-effective methods that can be easily implemented in resource-limited settings, e.g. under field conditions, are thus urgently needed to function as an early warning system for vector-borne disease epidemics. Our aim was to enhance entomological capacity in Nepal, a country with endemicity of numerous vector-borne diseases and with frequent outbreaks of dengue fever.

**Methods:**

We used a field barcoding pipeline based on DNA nanopore sequencing (Oxford Nanopore Technologies) and verified its use for different mosquito life stages and storage methods. We furthermore hosted an online workshop to facilitate knowledge transfer to Nepalese scientific experts from different disciplines.

**Results:**

The use of the barcoding pipeline could be verified for adult mosquitos and eggs, as well as for homogenized samples, dried specimens, samples that were stored in ethanol and frozen tissue. The transfer of knowledge was successful, as reflected by feedback from the participants and their wish to implement the method.

**Conclusions:**

Cost effective strategies are urgently needed to assess the likelihood of disease outbreaks. We were able to show that field sequencing provides a solution that is cost-effective, undemanding in its implementation and easy to learn. The knowledge transfer to Nepalese scientific experts from different disciplines provides an opportunity for sustainable implementation of low-cost portable sequencing solutions in Nepal.

**Graphical Abstract:**

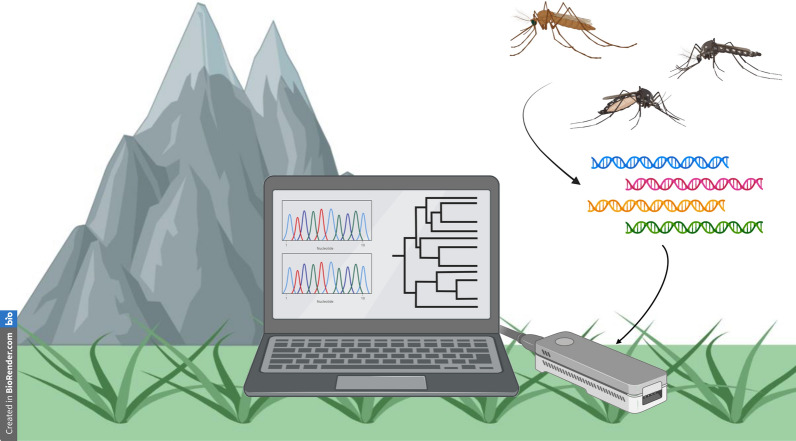

**Supplementary Information:**

The online version contains supplementary material available at 10.1186/s13071-022-05255-1.

## Background

Most vector-borne diseases (VBDs) result from infections with pathogens transmitted by arthropods. They encompass a substantial proportion (17%) of infectious diseases [1] and cause approximately 700,000 preventable deaths every year [1]. United global efforts are being undertaken to reduce the burden of VBDs. Despite scientific advances in the control of vector populations and ameliorating the consequences of infections, several VBDs, such as dengue fever, schistosomiasis and Lyme borreliosis, are still on the rise worldwide [2]. This concerns not only the tropical regions, classically associated with such diseases, but increasingly also temperate regions. In the USA, for example, the number of cases linked to diseases transmitted by mosquitoes, ticks and fleas tripled between 2004 and 2016 [3], and in Nepal, for instance, VBDs show significant expansion in geographical range [4].

During the past 2 decades, the world witnessed a surge in dengue fever (DF) cases following the spread of dengue virus (DENV) vectors as a consequence of globalization, trade and travel, land use change and deforestation [5–7]. While historically DF epidemics were limited in number and occurred in only few countries, DF is now endemic in > 100 countries [8], with the number and frequency of epidemics dramatically increasing [9]. This trend will most likely continue, as global warming is enhancing the suitability of previously unoccupied habitats for vector species [10]. Arbovirus vector species have already established in the regions deemed too cold for overwintering in Europe, the Americas and Asia [11–15], including the highlands of Nepal.

The first DF case in Nepal was reported in 2004 [16]. The number of infections has increased steadily since then, and Nepal witnessed its largest DF epidemic so far in 2019 with > 14,000 confirmed cases [17], although underreporting is likely [18]. The distribution of DF is negatively influenced by increasing elevation with the highest risk of infection at < 500 m above sea level (asl) [17]. Alarmingly, during the 2019 outbreak, the capital city Kathmandu, with 1.4 million inhabitants, at an elevation of 1400 m asl, was especially hard hit [19], while only sporadic cases were reported earlier [20]. Cases have also been reported from even higher elevations (2100 m asl), and the most likely driving factor for the distribution of vector species in the regions of higher elevation is increasing temperature associated with anthropogenic climate change [4, 17].

The most important vector species of DENV are the yellow fever mosquito *Aedes aegypti* (Linnaeus, 1762) (Diptera: Culicidae) and the Asian tiger mosquito *Ae. albopictus* Skuse, 1894. Both species are distributed throughout the tropical and subtropical regions, although *Ae. albopictus* has a markedly wider distribution range that extends into temperate regions because of their higher ecological plasticity and cold tolerance [21–23]. The increasing spread of both species in the temperate regions and subalpine zones of Nepal is probably driving the escalation of DF epidemics [24–26].

DF, however, is not the only VBD in this region, and the increasingly alarming situation regarding its spread must not influence the financial and human resources allocated to control other vector-borne diseases such as malaria, lymphatic filariasis, visceral and cutaneous leishmaniasis, chikungunya and Japanese encephalitis [25, 27]. With the exception of leishmaniasis, the disease agents are transmitted by mosquito species belonging to the genera *Aedes* Meigen, 1818, *Culex* Linnaeus, 1758, and *Anopheles* Meigen, 1818, with oftentimes several pathogens sharing the same vector species [28]. For all discussed diseases, entomological data on occurrence and distribution ranges of vectors are paramount to assess the risk of outbreaks and inform early warning systems, which will provide sufficient time to prepare medical health care professionals and generate awareness in the potentially afflicted populations.

Classically, species identification is done via distinct morphological traits. However, this requires extensive entomological training and expertise and is rather time consuming [29]. Alternatively, next-generation sequencing techniques can be relatively cheap and less time-consuming and offer simultaneous identification of numerous mosquito individuals [30]. NGS sequencing thus can aid classical morphological species identification provided that a reference sequence database exists [31]. Recent studies show that with a portable MinION sequencer (Oxford Nanopore Technologies, UK), barcoding can be conducted under field conditions [32], while simultaneously sequencing many individuals [33, 34]. Thus, field sequencing provides a fast, accurate and cost-effective alternative for morphological species identification.

**Fig. 1:**
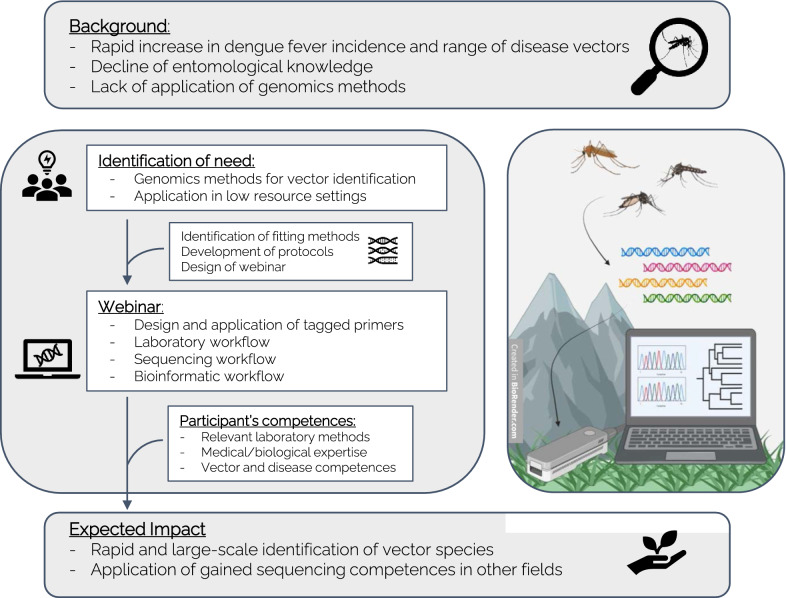
Summary of described actions to strengthen entomological capacity in Nepal

This technique offers accessibility of sequencing in resource-limited settings, such as in developing countries or in remote areas [32, 35–37]. Access to classical sequencing approaches in those settings can be limited by a lack of funding, a lack of infrastructure or logistical issues. These limitations apply to the situation in Nepal [38], especially to the survey of mosquitoes in regions that are oftentimes difficult to reach and make timely analysis of samples impossible [39, 40]. Therefore, our aim was to establish a barcoding pipeline (Fig. 1) for mosquitoes that is applicable in the field and supports current entomological efforts in reliably identifying vector species. As a secondary objective, we provided training to health care professionals and researchers in Nepal on the implementation of the pipeline.

## Methods

### Mosquito samples used for barcoding

The testing of the barcoding pipeline was conducted with four batches of mosquito samples that were obtained from different sampling campaigns and geographic areas with the aim to encompass different mosquito life stages (adults and eggs) and history of sample storage (Table 1). The NP1 samples comprised adult *Aedes*, *Anopheles* and *Mansonia* species collected in Nepal in 2013 [25], which were homogenized and stored at − 20 °C. The NP2 samples were unidentified adult *Aedes*, *Anopheles*, *Armigeres* Theobald, 1901, and *Mansonia* Blanchard, 1902 mosquitoes sampled along a climate gradient in Nepal in 2018 ([23]; NHRC Ref No 748/2017) and stored in 100% ethanol. The BEL samples (MEMO project CES-2016-02) were adult *Aedes* mosquitoes that were sampled in Belgium from 2017 to 2019 and stored dry at room temperature. These samples were included to test the influence of storage technique on the sequencing pipeline. The GER samples were eggs of the genus *Aedes* that were collected from graveyards in Hesse, Germany, during routine monitoring [Senckenberg Biodiversity and Climate Research Centre (SBiK-F), Frankfurt and Institute of Occupational Medicine, Social Medicine and Environmental Medicine, Goethe University Frankfurt] in 2018/2019 and stored at − 20 °C. As the pipeline only works on individuals or pools of individuals of the same species, pools of ten morphologically pre-sorted eggs each were used to test the barcoding pipeline.

**Table 1:** Overview of the samples used to test the barcoding pipeline

Sample code	Origin	Sampling year	Genera	Life stage	Storage conditions	Number of tested individuals	DNA extraction	Method to verify accuracy
NP1	Nepal	2013	*Aedes*, *Anopheles*, *Mansonia*	Adults	Homogenates, − 20 °C	15	Qiagen DNeasy	Morphology
NP2	Nepal	2018	*Aedes*, *Anopheles*, *Armigeres*, *Mansonia*	Adults	100% ethanol, room temperature	20	Lucigen	Sanger
BEL	Belgium	2019	*Aedes*	Adults	Dried, room temperature	8	Lucigen	Morphology
GER	Germany	2018/2019	*Aedes*	Eggs (pools of 10)	− 20 °C	28 pools	Qiagen DNeasy	Sanger + Morphology

The samples NP1 and BEL had been morphologically identified to species level, whereas the species identity of the samples NP2 and GER was unknown. All the morphologically identified samples were analyzed blindly during PCR amplification, and sequencing steps and the species status were verified afterwards. The species identities of samples NP2 and GER as identified through the Oxford nanopore barcoding pipeline were verified by Sanger sequencing.

### Oxford nanopore sequencing workflow

For DNA extraction from undamaged adult mosquitos (NP2, BEL), two legs of each mosquito were used, leaving the remaining individual intact for morphological analysis or further Sanger sequencing to verify Oxford nanopore results. The legs were placed in 20 µl QuickExtract solution (Lucigen, Middleton, WI, USA) and heated to 65 °C for 15 min and 98 °C for 2 min. DNA from homogenized adults (NP1) was extracted with the DNeasy Blood and Tissue kit (QIAGEN). We adapted the protocol to fit the lesser volume of used homogenate by using 50 µl of the homogenate, adding 10 µl proteinase K, 100 µl buffer AL and 100 µl ethanol. Elution was done in 50 µl Buffer AE to increase DNA concentration.

DNA extraction from egg samples (GER) was similarly conducted with the DNeasy Blood and Tissue kit (QIAGEN) with the following modifications. (i) The eggs were manually cracked using a pipette tip or a toothpick. (ii) Samples were incubated in proteinase K solution overnight for at least 12 h. (iii) The elution step was done with either 30 µl or 50 µl Buffer AE, which was pre-warmed to 56 °C.

We chose cytochrome c oxidase subunit I [41] as a marker for barcoding, as it represents the most commonly used locus with the most extensive database available. To allow for a cost-effective protocol, individuals were multiplexed during library preparation and sequencing. To be able to obtain individual-based sequences, PCRs were done separately for each mosquito, using primers with individual marker sequences (hereafter tags). We used a dual-indexing approach after Srivathsan et al. [33] for tagging forward and reverse primers that allowed for marking individuals with unique tag combinations.

The PCR reaction contained 5 µl GoTaq G2 Colorless Mastermix (Promega, Mannheim, Germany), 0.3 µl tagged forward and reverse primers (10 pmol/µl), respectively, 2.4 µl nuclease free water and 2 µl isolated DNA. PCR conditions were as follows: initial denaturation for 5 min at 94 °C, followed by 35 cycles of denaturation at 94 °C for 30 s, annealing for 60 s at 45 °C and extension for 60 s at 72 °C, followed by a final extension step for 5 min at 72 °C. PCRs were additionally tested with the Bento Lab DNA workstation to assess usefulness, especially regarding field condition.

All sequencing runs were conducted with the MinION Mk1B sequencer (Oxford Nanopore) using R9 flow cells. We used the Ligation Sequencing Kit (SQK-LSK109) according to the corresponding protocol. However, we omitted DNA fragmentation and adjusted the magnetic bead-washing step so that the volume of added beads always matched the volume of the DNA solution (1:1) to avoid size selection against short reads. The PCR products were end-repaired by using the NEBNext Ultra II End-Repair/dA-tailing Module (New England Biolabs, Ipswich, MA, USA) and incubated for 5 min at 20 °C and for 5 min at 65 °C. This was followed by a clean-up step with AMPure XP magnetic beads (Beckman Coulter, Brea, CA, USA). Adapter ligation was conducted using NEBNext Quick Ligation Module (New England Biolabs) and the AMX adapter mix included in the Ligation Sequencing Kit. For the last AMPure bead clean-up, the volume of used beads was adjusted to 100 µl to match the reaction mix.

The library was loaded onto the R9 flow cell, and sequencing was conducted and monitored using the MinKNOW software (Oxford Nanopore). Basecalling was conducted in parallel using the integrated MinKNOW basecaller with the fast option.

### Bioinformatic pipeline

For the analysis of the data resulting from Oxford Nanopore sequencing (ONS), we used the *miniBarcoder* pipeline by Srivathsan et al. [33, 34]. Briefly, sequences were curated using *minibarcoder.py*, a script that encompasses the identification of primers, demultiplexing of sequences, alignment of sequences and subsequent majority consensus building. The demultiplexing step identifies matching sequences by their combination of forward and reverse tags. Following this, consensus sequences were aligned back to the original read set and error corrected using graphmap [42] and racon [43] with the script *racon_consensus.sh*. Resulting barcodes were further treated by an amino acid correction that specifically targets frame shifts, using the script *aacorrection.py*. The resulting consensus barcodes were used for species identification.

**Fig. 2:**
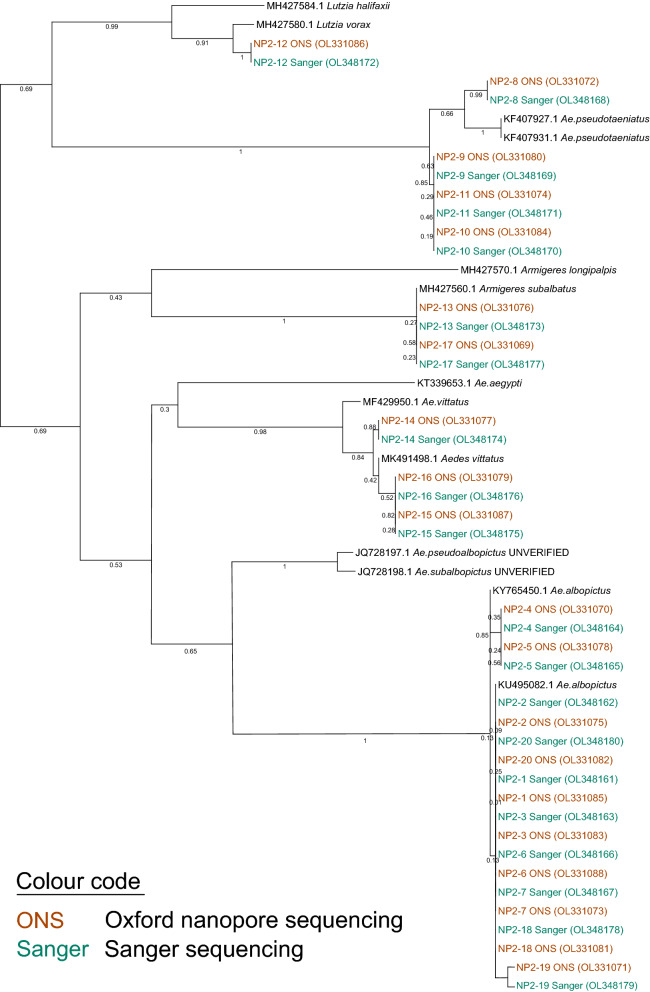
PhyML phylogeny (100 bootstraps) of NP2 samples with both Sanger (shown in blue) and Oxford Nanopore (shown in brown) sequences of the same individuals, showing perfect congruence between the two datasets. GenBank accession numbers are given in brackets. Sequences depicted in black are reference sequences that were morphologically verified (with the exception of JQ728197.1 and JQ728198.1)

We used two different approaches to identify mosquito species: a first step was to compare sequences to the GenBank database with BLAST or, when we suspected mismatched entries (i.e. when different species matched our sequences equally well), against the BOLD database. As an alternative identification approach, sequences (preferably those that were verified by morphological identification [44–49]) of species, that are common to the region from which the samples originated were downloaded from the NCBI database (for accession numbers, refer to Fig. 2, Additional file 1: Fig. S1) and aligned to the sequences obtained by ONS.

Libraries from NP2 and GER samples were consecutively run on the same flow cell. Even though we followed the recommended washing protocol in between runs, significant carryover of the NP2 library into the GER run took place. As we partly used the same identifying tags in both runs, the resulting demultiplexed datasets for those samples of the GER run contained two different species and no reliable consensus could be built with the pipeline described above as the samples contained too much variability. We thus used the output from the demultiplexing step of the pipeline (*minibarcoder.py*), i.e. a set of sequences that contain sequences from one NP2 sample and one GER sample that were marked with the same combination of identifying tags, to build alignments for each of the identified samples. Alignments were visually assessed and split into multiple alignments based on sequence similarity. Of those separated alignments, consensus sequences were used to determine which sequences belonged to the NP2 run, which was performed first following the above described bioinformatic pipeline. The remaining sequences then had to belong to the GER sample, and the consensus sequence was used to determine species identity via BLAST and for a combined phylogeny with Sanger sequences to verify the results.

### Verification of accuracy of mosquito barcoding

The accuracy of obtained sequences of the samples NP2 and GER was verified with the more accurate Sanger sequencing technique and compared to the sequences generated by ONS. Phylogenies with both resulting sequencing types were used to analyze the congruence of respective sequences. For NP2 samples, three legs of all individuals that were already sequenced with ONS were used for DNA isolations for Sanger sequencing. The PCR was conducted with untagged primers, and the reaction contained 5 µl GoTaq G2 Colorless Mastermix (Promega, Mannheim, Germany), 0.4 µl of each primer, 3.2 µl nuclease free water and 1 µl DNA. Cycler conditions were the same as for the PCR used for ONS. Sanger sequencing was conducted by BaseClear (Leiden, The Netherlands). Resulting forward and reverse sequences were aligned, and their consensus was aligned to the Oxford Nanopore sequences using Geneious (v. 10.1.3; Biomatters, New Zealand) with the default MUSCLE alignment algorithm. Reference sequences from common Nepalese mosquito species were added to the alignment, and a phylogenetic tree was built using the PhyML online tool with default settings and 100 bootstraps. The resulting tree was visualized using iTOL [50].

For GER samples, the same DNA extracts were used for Sanger sequencing as for ONS. The PCR reaction mix consisted of 1 µl 10 × reaction buffer (Projodis, Butzbach, Germany), 1 µl MgCl2, 1 µl dNTP mix (20 µm of each; Projodis), 0.1 µl MOLPol DNA Polymerase (Projodis), 0.2 µl of each primer, 5.5 µl ddH_2_O and 1 µl DNA. PCR conditions were 94 °C for 2 min followed by 35 cycles of 95 °C for 30 s, 48 °C for 1 min, 72 °C for 1.5 min and a final elongation at 72 °C for 110 min. The sequencing reaction conditions were 95 °C for 1 min, followed by 30 cycles consisting of 96 °C for 10 s, 50 °C for 10 s and 60 °C for 2 min. Capillary sequencing was performed on a 3730xl DNA Analyzer (Applied Biosystems, Waltham, MA, USA) at the SBiK-F laboratory centre. Resulting sequences were aligned to their respective Oxford nanopore sequences using Geneious Prime alignment with standard settings, and a phylogenetic tree was built, as described for samples NP2. Low-quality Sanger sequences (NP-A1, NP-A2, NP-A4, NP-C4, NP-D4, NP-E3, NP-G2,) were excluded from the alignment before the construction of the phylogenetic tree.

### Research capacity building for mosquito barcoding

The objective of the transfer of knowledge was to equip the participants with the methodology to perform molecular surveys of mosquitoes for species identification in field and low resource settings (see Fig. 1). Due to the COVID-19 pandemic, previously planned in-person training was adapted to an online course format with an accompanying handbook (Additional file 2). Six Nepalese specialists from different health research-related fields (microbiology, molecular medicine, health sciences, molecular parasitology) participated in the webinar. All the participants had prior experience of the required laboratory techniques. However, none of them had experience in working with the MinION nanopore sequencer or the Unix command line.

Four webinar sessions were conducted. The first session covered laboratory techniques from DNA isolation to library preparation and included an exercise on tagged primer design. The second session covered the theory behind the bioinformatics pipeline. After the second session the participants were provided with an installation manual of the bioinformatical programs (Additional file 3) to be installed in their computers. During the third session, questions about the software installation and general Unix commands were discussed. In the second part of the third session and the first part of the fourth session, the participants were able to try out the pipeline with a mock dataset. The second part of the fourth session was again used to discuss questions regarding the complete pipeline (Additional file 4).

A successful transfer of knowledge to the Nepalese participants of the Webinar was assessed by a questionnaire (for detailed questions see Additional file 1: Material S1). Specifically, the participants were asked to rate how well they were able to follow and participate in the different parts of the course. We furthermore asked the participants to rate how confidently they could apply what they learned with or without additional help. Lastly, they were asked to rate the helpfulness of the learned methodology to increase entomological knowledge and to support the vector-borne disease control efforts.

## Results

### Sequencing output

Each of the sequencing runs was stopped after enough data (amounting to a mean 20 × coverage per sample) had been produced to ensure reliable species identification (after 4–5 h). The obtained coverage varies between samples and sequencing runs (see Table 2). The samples from NP1 show the lowest coverage, which is in line with an observed low yield after DNA isolation and PCR (not shown). However, even from those samples, enough coverage was obtained for species identification. Furthermore, dry stored adults proved to yield enough DNA for analyses, similar to the samples stored in ethanol at room temperature for several years.

**Table 2:** Sequencing statistics for each sample type. Calculation of generated data and coverage was not possible for sample GER because of significant carry-over from a previous run on the same flow cell

Sample	Run time (h)	Reads generated	Data retrieved after basecall (.fastq)	Coverage after demultiplexing per sample	Variance in coverage between single samples
NP2	4:00	2.57 M	4.0 GB	37,578 ×	4151–135,874 ×
NP1	3:45	1.53 M	2.5 GB	3965 ×	6–13,307 ×
BEL	3:45			34,302 ×	416–97,623 ×
GER	5:00	NA	NA	NA	NA

Samples from NP1 and BEL were pooled for sequencing

### Accuracy of species identification

The species identification based on the Oxford Nanopore sequences was highly reliable. The accuracy of Oxford Nanopore sequences from the NP2 (Fig. 2) samples proved to be 100% in line with the less error-prone Sanger sequences. Regarding the GER egg pools, our adapted Oxford Nanopore barcoding pipeline mostly yielded the same results as the Sanger sequencing approach. A notable exception is the sample GER-G2, which was identified as *Ae. japonicus* (Theobald, 1901) with Sanger sequencing, while the Oxford Nanopore barcoding pipeline yielded two distinct alignments, which were identified as *Ae. japonicus* and *Ae. geniculatus* (Olivier, 1791; Additional file 1: Fig. S1). Morphological inspection of eggs prior to sequencing identified some of the eggs as *Ae. geniculatus*. The accuracy testing of species identification showed contrasting results for the samples NP1 and BEL, which were morphologically identified prior to ONS. For BEL samples, we found that the species identification based on sequencing and a subsequent BLAST step perfectly matched the morphology-based results (Additional file 1: Table S1). For NP1 samples, the congruence was much lower: of 15 sequenced samples, only 6 matched the morphologically identified species (40%; Table 3). Furthermore, the exact species identity of the samples NP1-2 and NP1-14 could not be resolved conclusively, as the entries in GenBank and BOLD are ambiguous (very high matches for both *An. subpictus* Grassi, 1899, and *An. jamesii* Theobald, 1901); however, neither was identified as their originally assigned species (see Table 3).

**Table 3:** Overview of identified species (NP1) using either classical morphological identification or ONS followed by a BLAST against the GenBank database or by a BOLD search

Sample	Species (morphological identification)	Species (ONS sequence)	Percent ident. (BLAST)	Matching of results	Accession number (ONS)	Reference for identification of sequencing result
NP1-1	*Ae. albopictus*	*Ae. albopictus*	98.6	Y	OL352190	Batovska et al. [49]
NP1-2	*An. splendidus*	*An. subpictus/jamesii*	98.5/98.3	N	OL352191	
NP1-3	*An. annularis*	*An. annularis*	98.8	Y	OL352192	Ashfaq et al. [45]
NP1-4	*An. barbirostris*	*An. barbirostris*	99.9	Y	OL352193	Saeung et al. [47]
NP1-5	*An. culicifacies*	*An. culicifacies*	98.8	Y	OL352194	Ashfaq et al. [45]
NP1-6	*An. maculatus*	*An. dravidicus*	99.4	N	OL352195	Ashfaq et al. [45]
NP1-7	*An. nivipes*	*An. annularis*	98.9	N	OL352196	Ashfaq et al. [45]
NP1-8	*An. sinensis*	*An. nivipes*	99.5	N	OL352197	
NP1-9	*An. athakani*	*An. lindesayi*	99.5	N	OL352198	Namgay et al. [46]
NP1-10	*An. culicifacies*	*An. culicifacies*	98.9	Y	OL352199	Ashfaq et al. [45]
NP1-11	*An. dravidicus*	*An. maculatus*	93.1	N	OL352200	
NP1-12	*An. fluviatilis*	*An. aconitus*	97.4	N	OL352201	Wilkerson et al. [48]
NP1-13	*An. nigerrimus*	*An. peditaeniatus*	99.7	N	OL352202	Ashfaq et al. [45]
NP1-14	*An. splendidus*	*An. subpictus/jamesii*	99.1/99.5	N	OL352203	
NP1-15	*An. tessellatus*	*An. tessellatus*	97.7	Y	OL352204	Bourke et al. [44]

The percentage of matching bases from the BLAST is given for the first shown result. Matching success of the two methods for species identification is 40%

### Transfer of knowledge for mosquito barcoding in Nepal

All the participants stated they were able to follow the lecture on DNA isolation, PCR protocols and the sequencing part of the pipeline. In the bioinformatics analysis, two third of the participants opted they were able to follow almost everything and one third that they were able to follow most parts. The question on whether participants were able to participate in the exercises was rated similarly. None of them had trouble with contents of the webinar or trouble to participate. The most time-consuming part of the webinar was the exercise on the analysis pipeline. Here, two thirds of the participants opted that they were able to comprehend everything, while one third said they were able to comprehend most parts.

One third of the participants were confident about applying the methods they learned without any additional help, and two thirds were somewhat confident. One third was again confident about applying what they learned only with the help of the provided handbook, while two thirds were mostly confident. When the participants could rely on help from the other participants, two thirds were confident that they could apply what they learned, while one third was somewhat confident. All the participants stated that the methodology would be helpful to increase entomological knowledge and support vector-borne disease control.

In general, the feedback on the parts of the webinar concerning laboratory techniques was more positive compared to the feedback on the bioinformatics part. Personal feedback from participants showed that this was due to their previous experience with laboratory techniques and little to no experience with bioinformatic analyses.

## Discussion

Portable field sequencing has been shown by other studies to be reliable for the identification of species [32, 35, 36]. Here we show that this technique is suited to identify mosquitoes at different stages of their life cycle and from different storage techniques. It is promising that even the pooled egg samples yielded enough DNA for reliable identification, which is useful especially when oviposition traps are used for monitoring. Our main aim was to aid in building entomological capacity in a country with several endemic vector-borne diseases and some on the rise. By hosting a webinar on the sequencing technique, hands-on protocols and ensuing bioinformatic analysis for Nepalese specialists with backgrounds in medical and biological sciences, we succeeded in the first important step to establish a field pipeline on next-generation barcoding in this country.

### Species identification

The accuracy of Oxford Nanopore based barcoding can be seen from both the correct identification and a high congruence compared to Sanger sequencing on a sequence level (Fig. 2). Indeed, given the higher rate of sequencing failures for the egg samples with the Sanger technique, the Oxford Nanopore approach might prove more robust, despite labor-intensive post-processing steps. Regarding the ambiguous results for the sample GER-G2, we assume that this egg pool consisted of a mixture of *Ae. japonicus* and *Ae. geniculatus* eggs. Since Sanger sequencing only results in a single output sequence, it is not possible to identify multiple species within a single sample. With Oxford nanopore sequencing, on the other hand, the output reflects the amplicons within the library and thus allows for the identification of mixed samples. This was however not possible using the pipeline described by Srivathsan et al. [33], which would result in a single sequence output. Instead, we aligned a subset of sequences, visually split the alignment based on sequence similarity and thus were able to identify two major subgroups of sequences that were used to identify both species. While this was not within the scope of this study, this example shows the potential of using next-generation sequencing for non-targeted species identification, e.g. for the identification of endosymbionts as also exemplified in Sonet et al. [51]. Especially in a potential VBD outbreak setting it would be highly advantageous to be able to not only identify mosquito species but also simultaneously detect a range of potentially harmful pathogens. Similar pipelines exist already to identify host, their ectoparasites and pathogen [52] or to identify different host species from the blood meals of mosquitoes [53] and triatomine bugs [54], but those need to be adapted to the specific vectors, pathogens and sequencing technique.

Due to our experiences with substantial contaminations from one sequencing run into the next, despite using the recommended flow cell washing steps, we advise against reusing a flow cell with different samples that are tagged with the same identifier sequences. In those cases, the described pipeline will yield empty results, as there will be too much sequence variability for the consensus calling step to work. However, since the pipeline worked without problems for samples that were tagged with unique identifiers not present during the first run, we do not see an issue with reusing a flow cell, given that there is no overlap in identifier combinations. One, however, needs to account for the reduced sequencing output for the second set of samples, since sequences from the first run that are still present on the membrane will compete for available nanopores.

Given the high accuracy and correct identification of other sequences that were identified with the Oxford Nanopore pipeline and the fact that we compared results to verified barcodes (Table 3), we assume that the individuals of the NP1 samples were not correctly identified by morphological assessment prior to homogenization. This again shows how genetic barcoding can aid in the correct identification of vector species. Especially in regions with high biodiversity, such as Nepal [55], the correct morphological identification of species can be difficult and needs extensive training. Most of the mismatches that occurred are known to be notoriously hard to discriminate morphologically because they belong to the same complex or group [56–59]. Morphological identification of similar specimens is even more challenging when samples and their discriminating features are damaged during trapping or transport. We were largely able to rely on morphologically verified entries of the barcode of life project [31] or GenBank to identify the sequencing results. However, it needs to be stressed that reliable reference databases are crucial to identify species correctly in the same way that trained and experienced entomologists are necessary to identify species morphologically [29] (Table 4).

**Table 4:** Overview of the advantages and disadvantages of next-generation sequencing barcodes compared to morphological identification

	NGS barcodes	Morphological identification
Costs	-Cheap when many individuals are multiplexed -Relatively high costs when only analyzing few samples	-Only costs are manpower given adequate equipment
Time	-Time per sample drastically reduced when using large-scale multiplexing	-Fast when only few individuals need to be identified
Training	-Few days of training needed for beginners	-Extensive training needed
Reliability	-Highly specific given adequate database	-Highly specific given adequate training, the existence of identification keys, and adequate morphologically discriminating characteristics
	-Adaptable to large range of species	-Different experts needed when analyzing different groups of species
	-Reliable for cryptic species	-Unreliable for cryptic species

### Application of barcoding pipeline for mosquitoes in field settings

The barcoding pipeline provides an opportunity to sequence large amounts of arthropods on a single flow cell of the Oxford Nanopore MinION sequencer [33, 34]. We optimized the barcoding pipeline for mosquito species and were able to show that high quality sequences can be obtained from different life stages of mosquito species and differently stored samples.

Given the very limited need for equipment, we are confident that this pipeline can be conducted in low-resource settings, provided access to electricity, as has been shown by projects that sequenced in remote rain forests [32, 35, 60], the desert [61] or even the International Space Station [37]. Especially when using adult samples and following the DNA extraction protocol by Lucigen, portable laboratory equipment such as Bentolab (Bento Bioworks Ltd., London, UK) can be used to supplement standard laboratory equipment (see also [60, 62]). Moreover, the relative simplicity of the pipeline provides an opportunity for easy and quick access to new users who have never previously worked with sequencers, as demonstrated by Watsa et al. [62].

### Research capacity building for entomological surveillance

The aim of this study is to enhance research and surveillance capacity in the framework of VBDs in the biodiverse and dengue-endemic country Nepal. However, a barcoding pipeline for mosquito surveillance can only be sustainably applied if in-country research capacity meets the basic requirements. The current development of scientific infrastructure (increase of R&D budget, implementation of high-tech equipment) and expert knowledge in Nepal is encouraging [38]. However, with the present resources, especially in light of the additional burden of the ongoing pandemic, it remains a challenge to adequately tackle rapidly expanding VBDs such as dengue [63]. In addition, entomological expertise, which is urgently needed for vector control programs, is lacking in Nepal [64]. These challenges are augmented by Nepal’s topography, with remote and poorly accessible regions [39]. All of this calls for easy to establish, cost-effective and mobile solutions to enable scientists to collect data onsite. NGS barcoding is currently the best solution for this, as it is able to handle large sample sizes [33], while being mobile and applicable in even the remotest locations [32, 35, 37, 60, 61], and provides comparably cheap sequencing costs of < 0.57 USD per sample for DNA isolation, PCR, library preparation and sequencing, when pooling ~ 3500 samples per flow cell [33]. The only challenge when pooling this number of samples is the labor-intensive PCR step, which leads to a trade-off between field-applicability and upscaling ability. After pooling the PCR products, the barcoding pipeline will yield results within a few hours, allowing for rapid identification, for example during outbreaks.

## Conclusion

While the identification of mosquito species is a crucial part in assessing the risk of outbreaks of several VBDs and quality control of interventions, the implementation of the barcoding pipeline has the potential for more large-scale and sustainable impact and capacity building. There is an enormous potential for upscaling of the barcoding pipeline and simultaneous sequencing of 4000 individuals, as shown by Srivathsan et al. [33]. The barcoding pipeline thus provides a cost-effective solution to aid classical morphological species identification and can be applied on-site. The training of medical professionals and researchers from different fields provides an opportunity for a long-term implementation of portable sequencing techniques in Nepal and for the application of sequencing techniques in several related research fields outside of the scope of this study.

## Supplementary Information

**Additional file 1: Material S1.** Questionnaire, **Figure S1.** Phylogeny of GER Samples, **Table S1**. Comparison of Oxford nanopore sequencing and morphological identification results of the BEL samples.

**Additional file 2: **Handbook accompanying the webinar on molecular field techniques of vector identification.

**Additional file 3: **Installation guideline for programs needed during the webinar.

**Additional file 4: **Teaching material of the webinar.

## Data Availability

All sequences generated in this study can be found under the herein specified accession numbers in the GenBank repository. The education materials for the webinar are available in Additional files 2, 3, 4.
