# Supplementary material for: A barcoding pipeline for mosquito surveillance in Nepal, a biodiverse dengue-endemic country
Source: Parasit Vectors. 2022 Apr 24;15:145. doi: 10.1186/s13071-022-05255-1 (PMC9035287; doi:10.1186/s13071-022-05255-1)
Supplement: Supplementary file 1 — Additional file 1: Material S1. Questionnaire, Figure S1. Phylogeny of GER Samples, Table S1. Comparison of Oxford nanopore sequencing and morphological identification results of the BEL samples. [file 13071_2022_5255_MOESM1_ESM.docx]

Supplement:

**Material S1) Questionnaire**

**Question 1:**


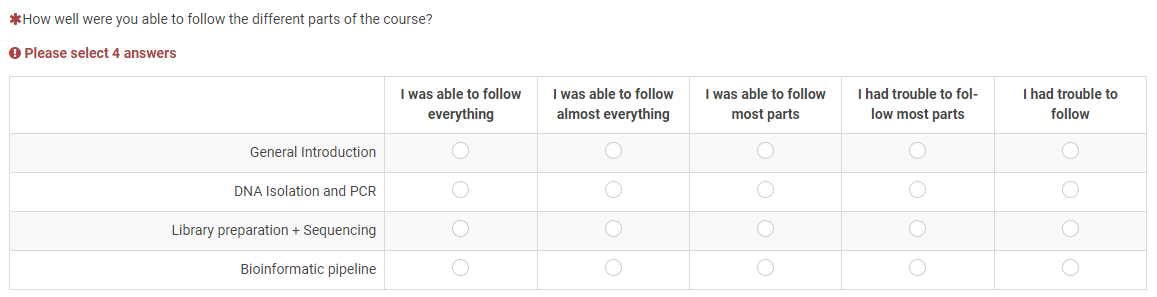


**Question 2:**


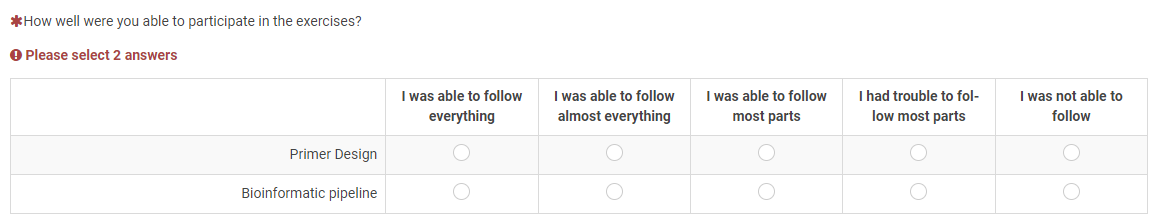


**Question 3:**


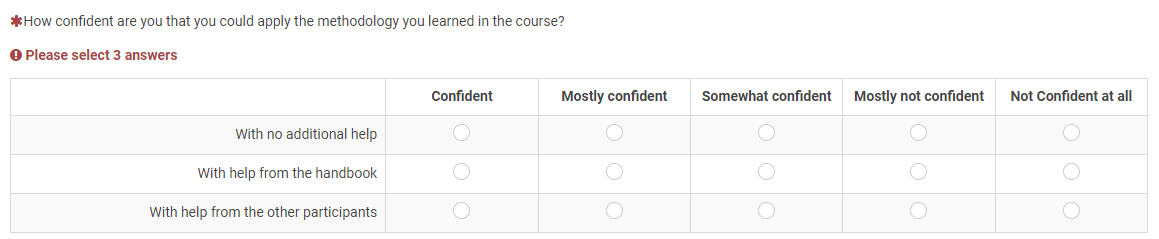


**Question 4:**


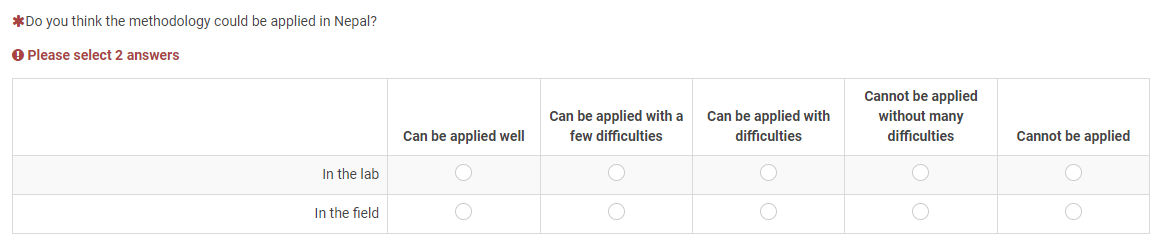


**Question 5:**


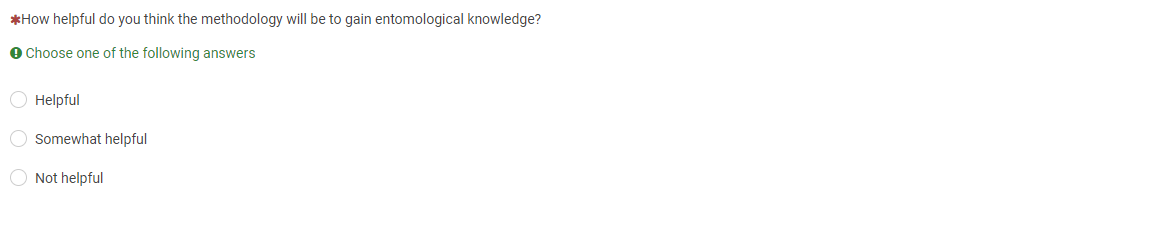


**Question 6:**


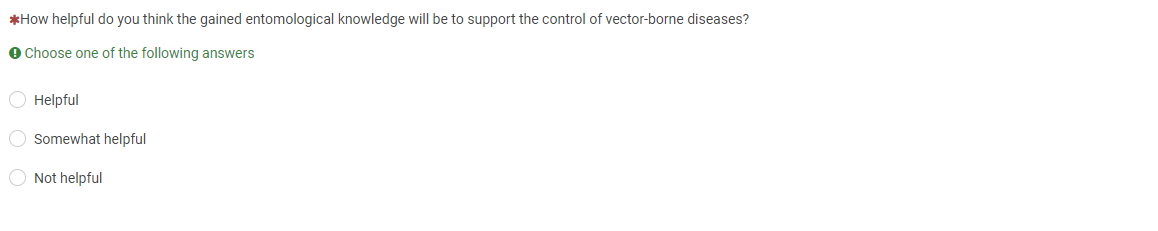


**Figure S1) Phylogeny of GER Samples: Comparison of Oxford Nanopore sequencing and Sanger sequencing**


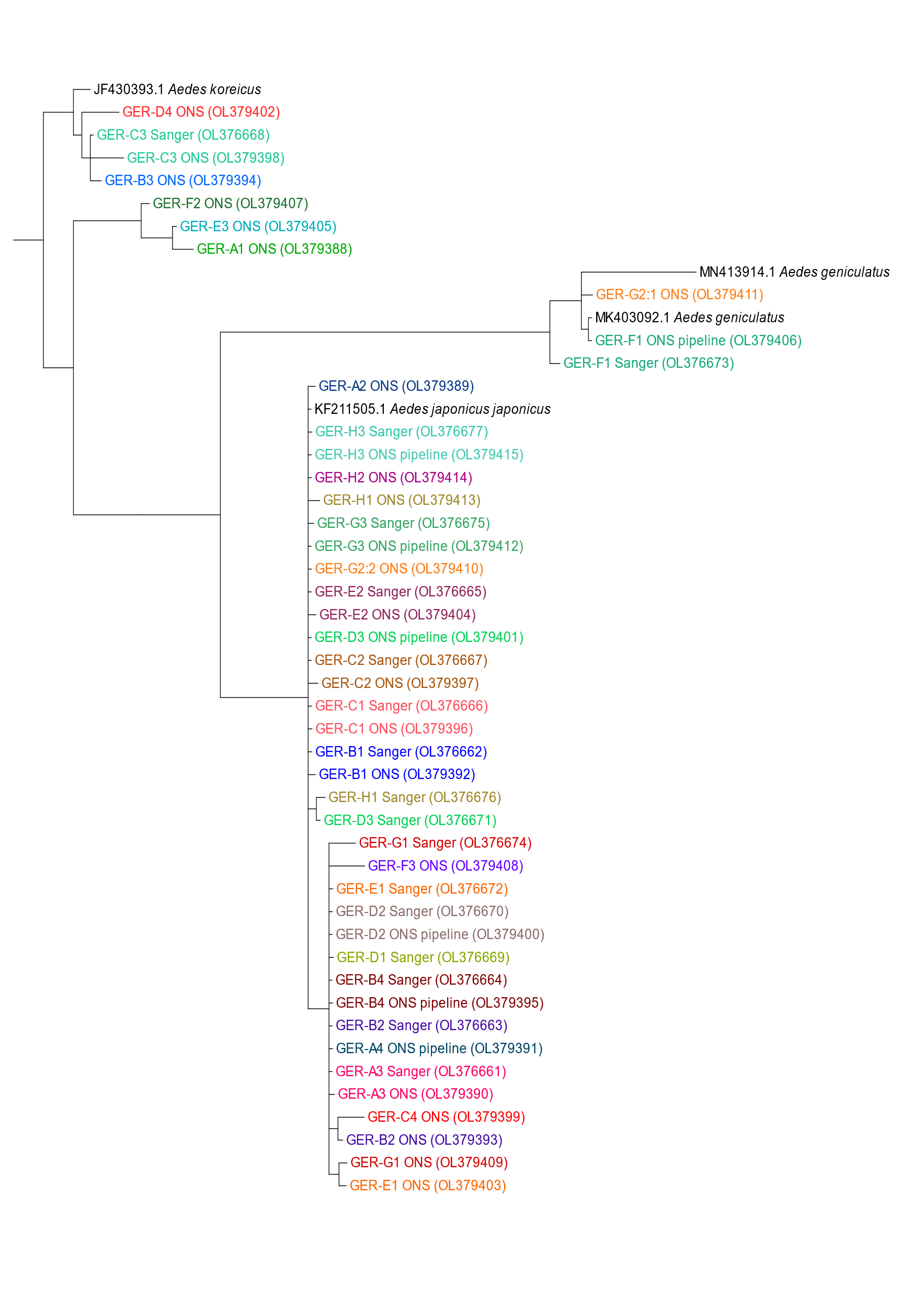


**Supplement B:** Phylogenetic comparison of sequences obtained by Sanger or Oxford Nanopore sequencing (ONS). Matching colors refer to the same individual. Sequence names depicted in black color refer to reference sequences that were added for species identification. GenBank accession numbers of the produced sequences are included in brackets.

**Table S1) Comparison of Oxford Nanopore sequencing and morphological identification of the BEL samples**

**Supplement C**: Results of morphological identification and Oxford Nanopore barcoding pipeline. Sequences were BLASTed against the GenBank database. Accession numbers are given for Oxford Nanopore sequences (ONS).

| Sample | Species (morphological identification) | Species (ONS sequence) | Percent ident. (BLAST) | Matching of results | Accession number (ONS) |
| --- | --- | --- | --- | --- | --- |
| BEL-S1 | *Aedes sticticus* | *Ae. sticticus* | 99.39 | Y | OL348195 |
| BEL-S2 | *Aedes caspius* | *Ae. caspius* | 99.85 | Y | OL348196 |
| BEL-S3 | *Aedes sticticus* | *Ae. sticticus* | 99.39 | Y | OL348197 |
| BEL-S4 | *Aedes vexans* | *Ae. vexans* | 99.70 | Y | OL348198 |
| BEL-S5 | *Aedes rusticus* | *Ae. rusticus* | 99.54 | Y | OL348199 |
| BEL-S6 | *Aedes geniculatus* | *Ae. geniculatus* | 98.78 | Y | OL348200 |
| BEL-S7 | *Aedes koreicus* | *Ae. koreicus* | 99.54 | Y | OL348201 |
| BEL-S8 | *Aedes japonicus* | *Ae. japonicus* | 99.70 | Y | OL348202 |
