# Supplementary material for: A barcoding pipeline for mosquito surveillance in Nepal, a biodiverse dengue-endemic country
Source: Parasit Vectors. 2022 Apr 24;15:145. doi: 10.1186/s13071-022-05255-1 (PMC9035287; doi:10.1186/s13071-022-05255-1)
Supplement: Supplementary file 2 — Additional file 2: Handbook accompanying the webinar on molecular field techniques of vector identification. [file 13071_2022_5255_MOESM2_ESM.pdf]

Handbook on molecular field techniques of vector identification

Dr. Juliane Hartke

Institute of Tropical Medicine, Antwerp

Unit of Entomology

Vector identification under field conditions

Sequencing in the field can be a challenge, as protocols that work well in a laboratory environment need to be adapted to be applicable in the field and should be designed in a way that ensure minimal handling steps and resource requirements. Additionally, a few tools have been released over the past years that can be helpful in setting up a working environment in the field. Classically, most pipelines from DNA extraction to sequencing preparation are dependent on standard laboratory equipment, such as centrifuges, vortex mixers, microfuges, and thermal cyclers. Some of these components are available as a smaller version that can be taken into field settings and that only require electricity. There is miniPCR, a thermal cycler that encompasses up to 16 wells and can be programmed with the help of a mobile phone. And there is Bentolab, a portable mini laboratory that has the size of a laptop and encompasses a thermal cycler with 32 wells, a microfuge, and a gel electrophoresis. Sequencing is now also possible under field conditions with the Oxford nanopore MinION sequencer.

Principle of vector identification with the Oxford nanopore MinION sequencer

While this particular sequencing technique comes with several advantages (i.e. sequencing in the field becomes possible), one big disadvantage is the high error rate, which complicates the reliable identification of species. Differences between recently diverged species are often minimal and sequence divergence of only 2% can be indicative of distinct species. When using the MinION sequencer, the question is: Are the differences we observe between sequences caused by the error rate, or because they belong to different species?

Before deciding whether sequences belong to different species, error correction should be done to avoid this problem. To error-correct sequencing *reads*[1], reads that belong to the same individual need to be *aligned*[2] and the consensus sequence formed. By comparing different information for the same nucleotide position across different reads, errors can be corrected by using the majority rule. To be able to group reads of the same individual, sequences need to be marked with distinct identifiers that can be sorted bioinformatically after sequencing.

ATGCAGTAAGGCATTAACGG**C**AATTCAT
ATGCAGTAAGGCA**A**TAACGGGAATTCAT
ATGCAGTAAGGCATTAACGGGAATTCAT
AT**T**CAGTAAGGCATTAACGGGAATTCAT
ATGCAGT**C**AGGCATTAACGGGAATTCAT
ATGCAGTAAGGCATTAACGG**C**AATTCAT
ATGCAGTAAGGCA**A**TAACGGGAATTCAT
⬇
ATGCAGTAAGGCAATAACGGGAATTCAT

[1]Read = nucleotide sequence that corresponds to a DNA fragment that has been sequenced
[2]Aligning = arranging sequences in a way that the same positions on the genome are written below each other

Principle of tagged barcoding

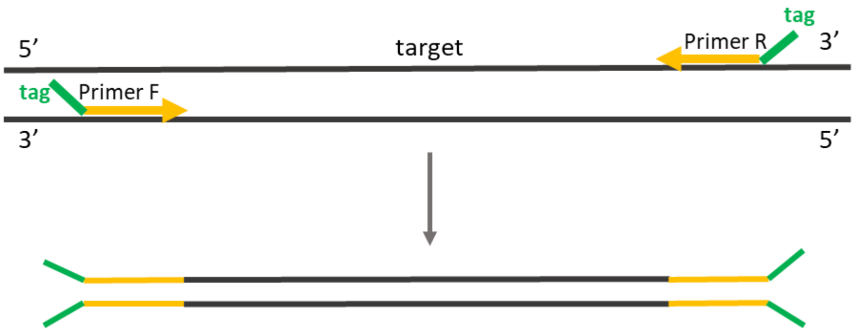

During PCR we amplify a target sequence by choosing appropriate primers. In this protocol, we want to additionally mark the sequences with individual identifier sequences (hereafter "*tags*"). During ordering of our normal barcoding primers, we can simply add a unique sequence of 13 base pairs in front of our forward and reverse primers. The resulting PCR products are marked with unique marker sequences.

We use *combinatorial tags*. This means, that for each individual the identifiers are only unique by combination. The advantage to unique barcodes for each individual is that less material is needed, and costs are kept low. With a combination of 4 forward and 4 reverse tagged barcodes 16 individuals can be uniquely tagged and sequenced. Upscaling is possible and has already be done for 96x96 barcodes, which yield 9.216 unique combinations.

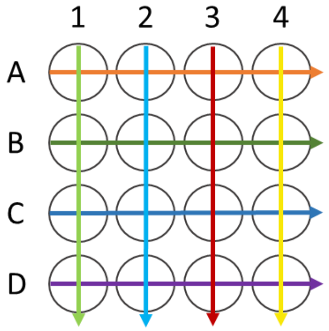

ATCCGGTCGGAGA GGTCAACAAATCATAAAGATATTGG

tag Primer

There are a few rules that need to be followed for choosing appropriate tag sequences:

1) Tags should not include homopolymeric stretches of sequence (= single nucleotide repeats of more than 2 bp).
2) Tags should not end in 2-base repeats if the primer starts with the same 2 bases
3) Different tags cannot share > 6 bp sequence stretches. These tags could not be combined in the same PCR reaction due to possible primer-primer interactions.
4) Tags need to be different enough from each other to account for MinION error rate. Calculate with 3 bp errors of any kind and combination
5) Length of tag is a trade-off between demultiplexing rate and PCR success. ~13 bp is a good compromise.

For already curated tag sequences that match these requirements, refer to *Srivathsan et al. 2019, BMC*.

DNA Isolation Protocol:

Material:

- P20 or P1000 pipette with tips
- Precise forceps
- Petridish or Eppendorf Tube
- 0.2 µl PCR tubes for each individual
- QuickExtract solution from Lucigen
- Purified water
- Heatblock or other equipment that can reach 98°C

Protocol:

- Take 2 legs of each mosquito. Try to sever the legs directly at the body
- Wash the legs in purified water to get rid of potential debris
- Place legs in 0.2 µl thin-walled PCR tubes with 20 µl QuickExtract solution. Make sure the legs are covered.
- Incubate at 65 °C for 15 minutes and then 98 °C for 2 minutes

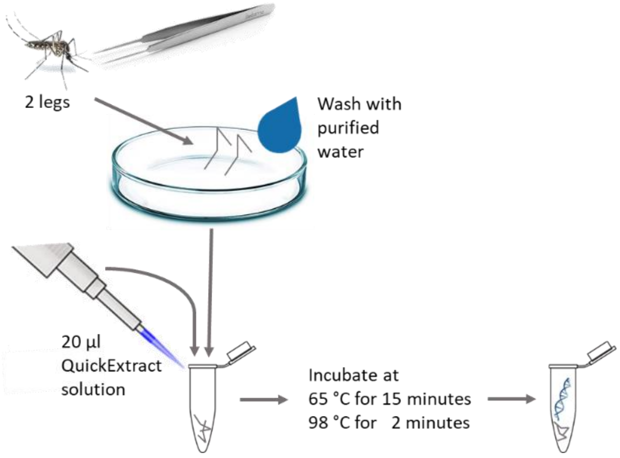

PCR Protocol:

Material:

- P5 pipette and higher (depending on number of samples) with tips
- 0.2 µl PCR tubes or PCR plates (depending on number of samples)
- Nuclease free water
- Primer
- PCR Mix
- DNA from DNA Isolation step

Protocol:

PCR Reaction:

- 5.0 µl GoTaq G2 Colorless Mastermix (GoTaq G2 DNA Polymerase, dNTP, MgCl2, reaction buffer; Promega, Mannheim, Germany)
- 3.6 µl H2O
- 0.2 µl tagged Forward Primer
- 0.2 µl tagged Reverse Primer
- 1.0 µl DNA

Cycler settings:

94 °C	5 min	
94 °C	30 sec	35x
45 °C	60 sec	35x
72 °C	60 sec	35x
72 °C	5 min	

Since the Primer combinations have to be unique for each individual and each reaction, a bulk reaction mix including the primers is not possible. This also makes a good plan paramount, since after sequencing, the sequences need to be assigned back to individuals. It is therefore necessary to know the correct tag combinations for each individual.

Gel electrophoresis:

When establishing the PCR protocol, a gel electrophoresis step of all samples is necessary to check the successful amplifications of the target sequence. Wanted results are: all bands are visible, no double bands, no excessive primer clouds, products are around 800 bp long.

Sequencing technique:

Oxford nanopore sequencing is a relatively new technique. The sequencing takes place in the so-called flow cell. The flow cell contains a membrane with 2048 nanopores that are under a constant electrical current. During sequencing, a DNA strand can pass through a nanopore and will elicit a change in electrical current depending on the base that passes through the pore. This signal can be translated back to the nucleotide sequence during a process that is called basecall.

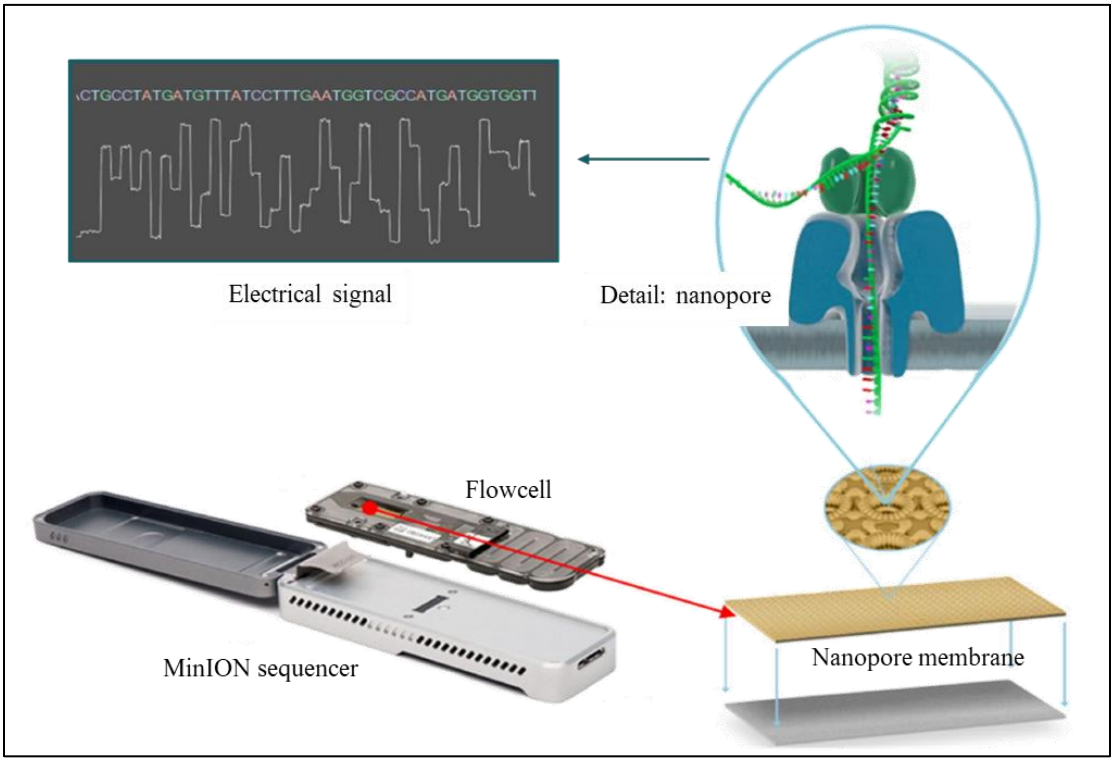

There are several advantages and disadvantages of this technique. The biggest advantages are that the technique is relatively easy to apply even in small laboratories and is comparably cheap. Due to the size of the sequencer, sequencing can be conducted under field conditions and in remote places that normally do not have access to sequencing. Furthermore, unlike other sequencing techniques, here, there is no limit in sequence length. The length distribution of the sequencing output reflects the length distribution of the DNA input.
The biggest disadvantage is the high error rate compared to other sequencing techniques. Users should generally calculate with an error rate of 5%, but this number depends on the type of molecules and library preparation.

Library Preparation Protocol

What does library preparation mean?
The process of library preparation prepares DNA for sequencing according to a protocol that is specific to the sequencer that is being used. Independent of the sequencing technique, every library preparation has the goal to attach sequencing adapters to the DNA. In the case of Oxford nanopore sequencing with the MinION sequencer, these sequencing adapters include a motor protein that unwinds the double stranded DNA and slows down the speed with which the DNA strand passes through the nanopore. A second component is the so-called tether that improves the DNA's sensitivity to the nanopore.
There are different library preparation protocols available, depending on the starting material and application. This protocol focuses on the Ligation sequencing kit (SQK-LSK109) and the library preparation workflow "genomic DNA by ligation".
During the planning phase of a sequencing experiment, it is advisable to consult the Oxford nanopore homepage. Parts of the homepage and resources are only accessible upon registration. Afterwards, training videos and in-depth explanations can be accessed. Protocols for library preparation are available in several different versions. The "getting-started-guide" versions are for sequencing beginners and encompass more detailed explanations for each step and even contain videos. The long version of the protocol is advisable to use if more guidance is required, and the checklist version should only be used by experienced users.
It is also advisable, before every sequencing run, to check for updated protocols and IT requirements, as well as software updates.

Material for the genomic DNA by ligation protocol:

- MinION sequencer
- MinION flow cell
- Computer that fulfills MinION standards
- Ligation sequencing kit (SQK-LSK109)
- Magnetic rack or strong magnet
- Microfuge
- Vortex Mixer
- Thermal cycler
- P2, P10, P20, P100, P200 and P1000 Pipettes and tips
- 0.2 µl PCR tubes

- 1.5 ml Eppendorf LoBind tubes or similar
- AMPure XP beads
- NEBNext FFPE Repair Mix
- NEBNext Ultra II End repair/dA-tailing Module
- NEBNext Quick Ligation Module
- Nuclease free water
- 70% Ethanol (in nuclease free water)
- Ice

Before starting with the library preparation, read the protocol carefully, make sure all reagents and equipment are available, and prepare the reagents according to the protocol.

LNB	LNB: Ligation Buffer	Thaw at room temperature, spin down, place on ice
SFB	SFB: Short Fragment Buffer	Thaw at room temperature, mix by vortexing, spin down, place on ice
EB	EB: Elution Buffer	Thaw at room temperature, mix by vortexing, spin down, place on ice
DCS	DCS: DNA control strand	Thaw at room temperature, mix by pipetting, spin down, place on ice
FRB	FRB: NEB FFPE DNA Repair Buffer	Prepare according to manufacturers instructions, place on ice
FRM	FRM: NEB FFPE DNA Repair Mix	Prepare according to manufacturers instructions, place on ice
URB	URB: Ultra End-prep Reaction Buffer	Prepare according to manufacturers instructions, place on ice
UEM	UEM: Ultra End-prep Enzyme Mix	Prepare according to manufacturers instructions, place on ice
T4	T4: NEBNext Quick T4 DNA Ligase	Spin down, place on ice
	AMPure XP beads	Place at room temperature

The first step is to pool all PCR products that should be sequenced. Refer to the Genomic DNA by Ligation sequencing protocol for the required amount of DNA input. Pool the PCR products in such a way that they meet the requirements. DNA quantification with Qubit and Nanodrop is recommended at this step to ensure the success of the sequencing run. Oxford nanopore specifies the recommended amount in fmol. There are several online conversion tools that help to calculate the quantity in this unit of measurement (e.g. bioline.com).

The general steps of Oxford nanopore library preparation with the Ligation sequencing kit are the following:

1) **DNA repair, End-prep, dA tailing** (repair of DNA damage and nicks, generate blunt ends, generates 3’ adenine and 5’ phosphate overhangs)

2) **Cleanup** (remove unwanted material from the reaction volume with magnetic beads)
3) **Adapter** ligation (adapters with T-tail overhangs are ligated to the 3’ adenine overhangs)
4) **Cleanup** (remove unwanted material from the reaction volume with magnetic beads)

1) DNA repair, End-prep, and dA tailing

During the first step of library preparation, the aim is to prepare the DNA for the ligation of the sequencing adapters. To this end, nicks and damages in the DNA will be repaired and overhanging ends will be filled to blunt ends. Afterwards, to the 3’ ends adenine overhangs will be ligated, and to the 5’ ends phosphate overhangs will be ligated. These serve as anker points for the sequencing adapters.

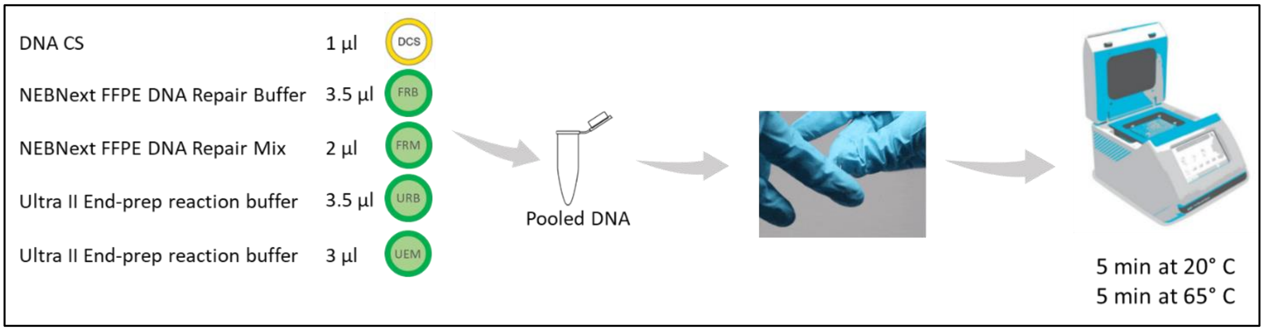

The required reagents will be added to the DNA sample, mixed by flicking the tube thoroughly, followed by an incubation step for 5 minutes at 20 °C and 5 min at 65 °C.

2) Cleanup

Before the ligation of adapters, a DNA clean-up step has to be performed. The DNA-mixture at this moment, still contains leftover reaction reagents from DNA isolation, PCR, and end-prep. To ensure that adapter ligation and in the end also sequencing, works without problems, these unwanted components should be removed at this step.

Nanopore recommends a magnetic bead cleanup. Depending on the conditions, the polar phosphate backbone of the DNA molecules can bind to the negatively charged carboxyl coating of the beads. By letting the DNA bind to the magnetic beads and by aggregating the beads to one side of the tube, all unwanted material can subsequently be removed from the solution.

a) Adding the beads

For this step, make sure the beads are properly mixed and have been warmed up to room temperature. By choosing a particular ratio of bead-solution:DNA-solution, size selection is possible. A ratio of <1:1 bead:DNA will select for longer DNA fragments, a ratio of >1:1 bead:DNA will select for shorter fragments. As the PCR step already ensured a uniform size distribution, size selection is unnecessary. The beads will thus be added with a ratio of 1:1 bead:DNA, which will only select against very short DNA fragments of <100 bp (primer dimers, free adapters etc).

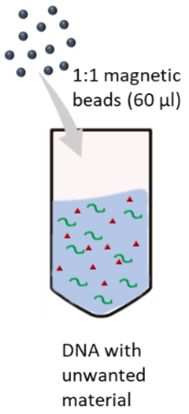

The solution is then incubated at room temperature for 5 minutes and should be moved constantly by slowly turning the tube across all axes.

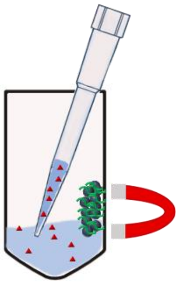

b) Formation of pellet

The DNA that is bound to the magnetic beads can now be easily separated from the unwanted material in the solution by binding the beads to the side of the tube by using a magnetic rack, or a strong magnet. Only continue with pipetting off the supernatant once it is visibly clear and all beads have moved into a pellet. Place the pipette tip on the side opposite the pellet and make sure that during pipetting the pellet is not disturbed.

c) Washing the pellet

The next step is to wash the pellet with 70% ethanol twice to remove any remaining unwanted material. For this, the tube has to remain in the magnetic rack, or alternatively, if a separate magnet is used, the magnet has to remain at the side of the tube. Again, care must be taken to not disturb the pellet. Therefore, do not pipette the ethanol directly on the pellet, but again, pipette slowly at the opposite side. Pipette the ethanol off again and repeat. Residual ethanol will inhibit the ligation of adapters. Therefore, all remaining ethanol needs to be removed. For this, shortly spin the tube in a microfuge, re-pellet the magnetic beads again and pipette off any residual ethanol. Let the pellet dry for 30 seconds (but not longer) to allow remaining ethanol to evaporate.

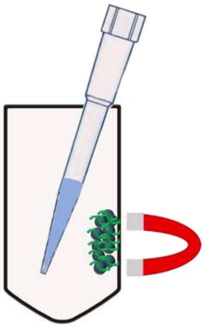

d) Resuspension

Resuspend the pellet in nuclease free water by continuously flicking the tube. It may take some time until the pellet is properly resuspended. Incubate at room temperature for 2 minutes. During resuspension, the DNA fragments will detach from the magnetic beads, resulting in a solution of nuclease free water, DNA fragments and magnetic beads.

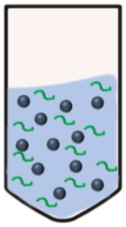

e) Transfer DNA to new tube

Now, all that remains is to pellet the beads again on a magnet. This time, the DNA is not attached to them and will remain in the nuclease free water. After the pellet has formed, the supernatant can be transferred into a new tube.

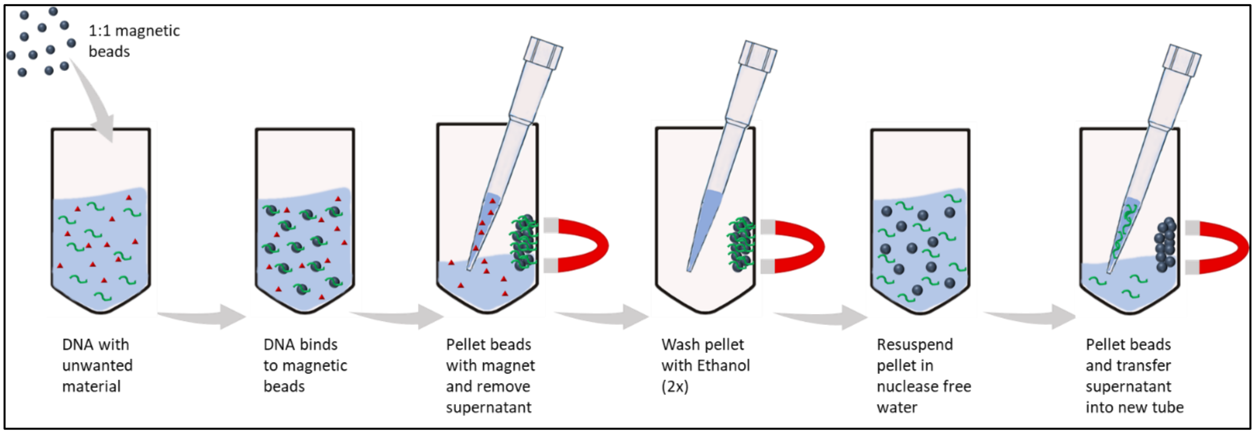

3) Adapter ligation

The next step of the library preparation is the adapter ligation step. Simply add all required reagents to the DNA from the previous cleanup step. Make sure to mix the ligation buffer by pipetting – due to its viscosity it will not mix by flicking or vortexing the tube. Afterwards, the solution is incubated at room temperature for 10 minutes.

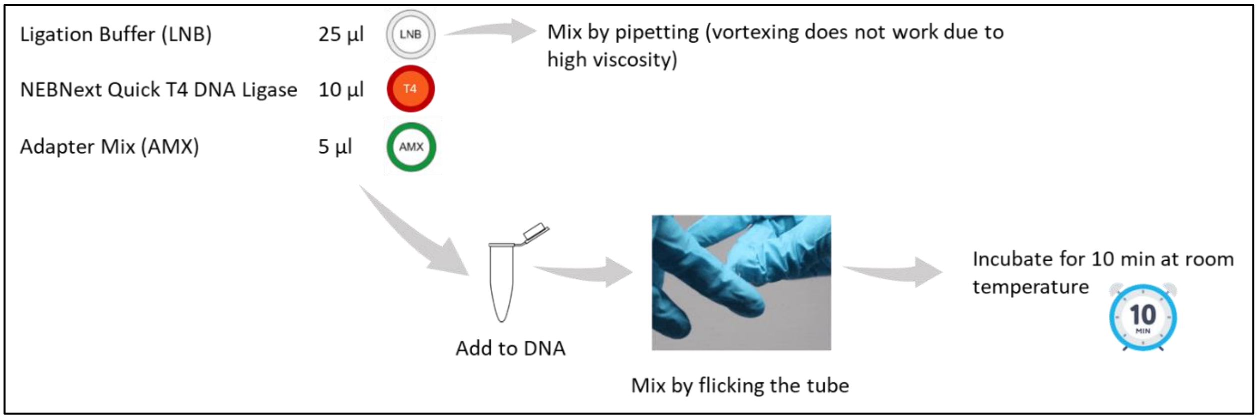

4) Cleanup

The resulting solution has to be cleaned again to prevent the reagents from the adapter ligation step from interfering with the sequencing process. Essentially, the cleanup follows the protocol from step 2), although this time the pellet is washed with Short fragment buffer and the DNA is resuspended in Elution buffer. Furthermore, the protocol at this step recommends a bead:DNA ratio of 0.4:1, however, this would result in selecting against short fragments. Therefore, the used ratio will again be 1:1.

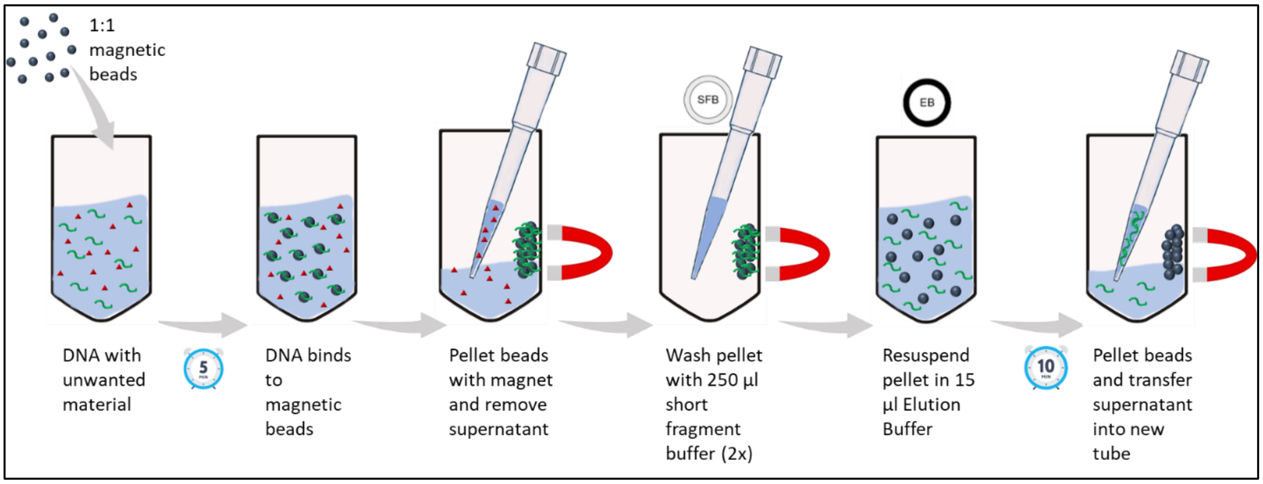

After this final cleanup step, the DNA library is finished. The DNA at this step has been repaired and end-prepped and possesses sequencing adapters.

Preparation of the flow cell

The Oxford nanopore flow cells are delivered containing a storage buffer, which allows for shipment and storage of the flow cells. Before sequencing, however, the buffer has to be exchanged for a sequencing buffer that will provide fuel for the sequencing reaction.

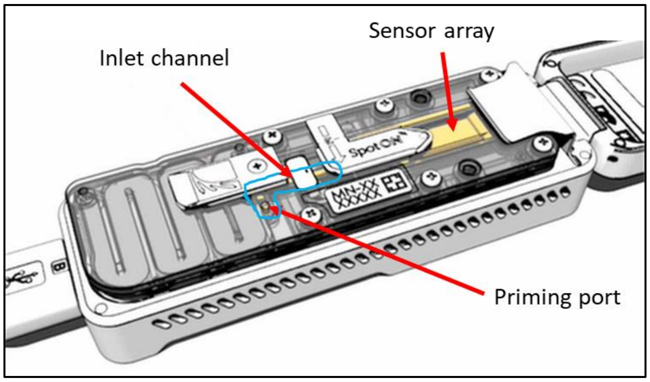

The flow cell consists of a *sensor array* with nanopores, where the actual sequencing will take place. The sensor array is connected to the *Priming port* by the *Inlet channel*. It is possible that an air bubble is introduced directly under the priming port. Before we load the flow cell with the sequencing buffer, the air bubble needs to be removed. Otherwise the sequencing buffer would push the air bubble to the sensor array, where it could irreversibly damage the nanopores.

To remove the air bubble, open the priming port cover by sliding it clockwise. Set a P1000 pipette to 200 µl, insert the tip into the priming port and slowly adjust the volume dial until a small volume of buffer enters the pipette tip. It is advisable to refer to the instructional video on the Oxford nanopore homepage for this step.

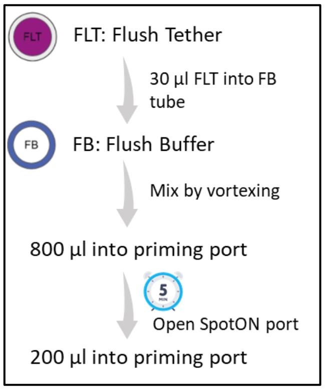

For the preparation of the sequencing buffer mix flush tether and flush buffer according the protocol specifications. Pipette the first load of sequencing buffer through the priming port into the flow cell. Make sure to pipette slowly and leave a tiny bit of buffer in the pipette tip to avoid the introduction of new air bubbles. Wait 5 minutes. Before pipetting the second load, open the SpotON port and then pipette the remaining sequencing buffer into the priming port. By opening the SpotON port, the sequencing buffer on the sensor array will rise a bit through the SpotON port and will expel any potential air bubbles that are on the sensor array.

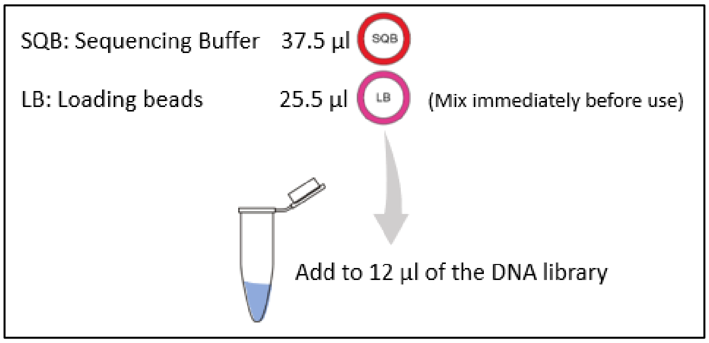

During the 5 minutes of waiting, prepare the library for loading according to the protocol. Mix the resulting library directly before loading by pipetting to make sure the loading beads are suspended properly. Add the library to the flow cell through the SpotON port in a drop wise fashion. Take special care that the pipette tip never touches the SpotON port, as this will damage the sensor array, and that each drop is gone before adding the next one.

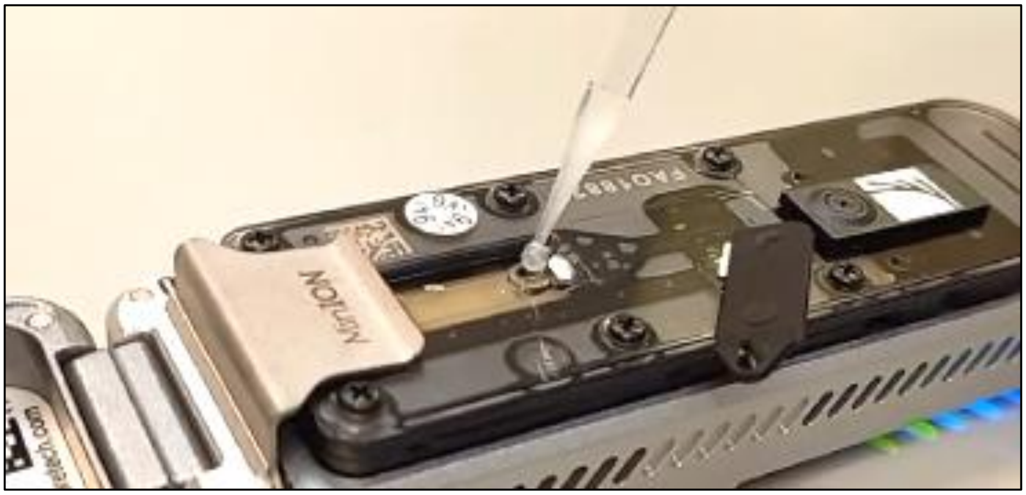

Close the SpotON port and the Priming port cover. The flow cell is now ready for sequencing.

Starting the sequencing run

General information:
The sequencing run is initiated from a computer that is connected to the sequencer. This computer has to fulfill the requirements from Oxford nanopore, which can change over time. It is thus advisable to check the computer requirements before conducting sequencing runs. The software that controls the sequencer is called MinKNOW. Before starting the sequencing run it is advisable to check for available updates.

An internet connection is required for initiating the sequencing run, when the standard version of MinKNOW is being used, however, it can be disconnected during the run. When sequencing in the field, an internet connection is not always possible. In this case, an offline version of MinKNOW can be requested from Oxford nanopore that is able to run without an internet connection.

The raw data that is created during the sequencing run is the change in electrical current that occurs when DNA strands pass through the nanopores. This raw data has to be translated back to the actual nucleotide sequence – a process that is called *basecalling*. Basecalling can already be conducted in parallel to the sequencing run via the MinKNOW software if the computer is connected to the internet. If there is no internet connection, basecalling can be conducted afterwards with another software, e.g. Guppy. For details refer to the nanopore homepage.

Step-by-step guide:

1) Check if the MinION is connected to the computer via the USB cable and if the flow cell in inside the sequencer
2) Open MinKNOW and install software updates if necessary (ideally, this should be done beforehand)
3) Login and choose the option “Start sequencing”
4) Choose a name for the sequencing run, select the type of flow cell that is being used and chose a sample ID

5) On the next page, choose the correct sequencing kit (in this example: SQL-LSK109). Here, also extension kits can be chosen if used, but in this example, only the Ligation sequencing kit was used.
6) On the next page, choose the run options. It is possible to limit the time of sequencing. If the default setting is chosen, the sequencing will run for 72 hours. However, it is possible to stop the sequencing at any time point during sequencing, e.g. when the desired amount of sequences has been reached. The voltage should be left at default settings. Voltage can be adjusted when reusing the flow cell, although opinions differ about best-practice.
7) On the next page, choose the basecalling options, if basecalling should be conducted with MinKNOW during the sequencing run. It is advisable to choose the fast basecalling option, as the slow option will most likely run for several days.
8) Lastly, select a location on your computer, where the sequencing data should be stored. Sequencing data can be saved in FAST5 and FASTQ format. It is recommendable to save data as both.

Afterwards, the sequencing run can be started.

Monitoring the sequencing run

<u>General information:</u>
The sequencing takes place on the sensor array inside the flow cell. The sensor array contains 2048 nanopores that are each located in a single well. Groups of four wells are connected to a nanopore sensor, or channel. Every 1.5 hours, the sequencing is paused, and the status of wells is assessed. This is called a mux scan. The first mux scan takes place right before the sequencing starts and each of the 512 channels is scanned and the status of all four nanopores that are connected to a channel are scanned. For each channel, the most promising nanopore is selected. This means that during sequencing, always 512 nanopores are active. Every 1.5 hours nanopores are checked and pores that became inactive are exchanged for a new active pore.
After starting the sequencing run, the sequencer will try to reach its optimum temperature for sequencing. This may take a few minutes. Afterwards, the pores of the flow cell will be checked and the quarter of pores that are most promising are chosen to sequence first.

<u>Monitoring:</u>

1) Overview of the status of single pores:

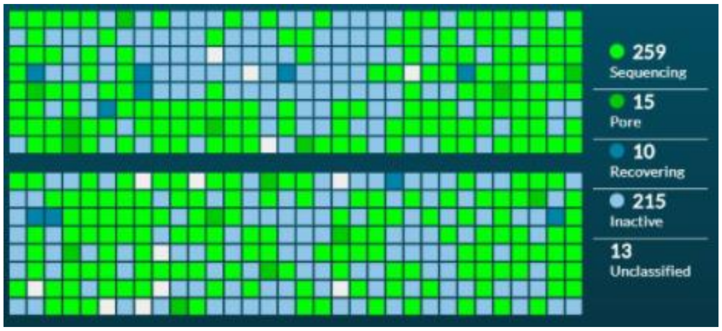

The most prominent and informative graph is the overview panel on the status of single pores. This overview depicts all currently active 512 pores. It is optimal, when most of the pores are bright green. The darker green means that a pore is currently active and waiting for DNA. The dark blue shows a pore that is not active but recovering, and the light blue shows an inactive pore that will not be available for sequencing anymore. A cluster of blue pores might be indicative of a bubble on the sensor array.

2) Cumulative output:

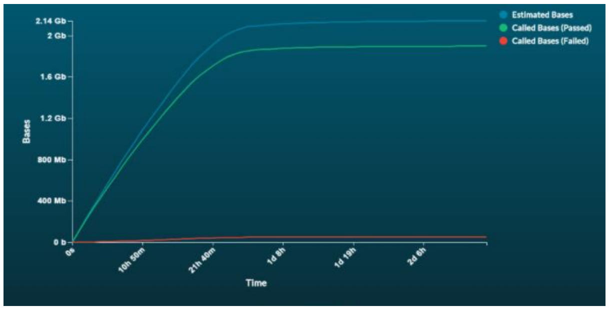

This graph will show the progression of the sequencing run and depict the cumulative output, either as the total number of bases or as the total number of reads that have been sequenced so far. The three different lines are a) the total bases or reads that have been sequenced, b) of those the number that have been basecalled and that passed the quality threshold, and c) the ones that did not pass the quality threshold. Ideally, this graph should not show much saturation, as this would mean that at some point no new sequencing output was generated.

3) Duty time

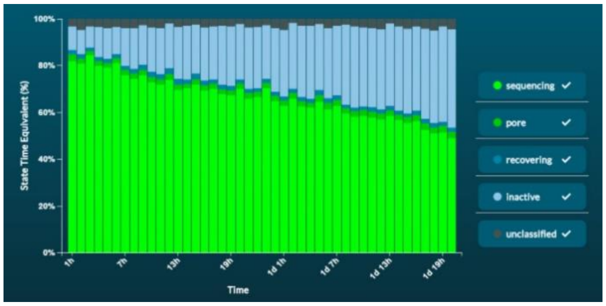

This graph essentially shows the same as the status of single pores graph, although here, the statuses of pores are summarized over time. A general decline of active and sequencing pores and an increase in inactive pores is to be expected during a sequencing run, as a flow cell only has a limited run time of about 72 hours. Most of the pores should be active and sequencing, and only a small proportion should have a darker green color. If many pores are waiting for a DNA strand, this could indicate that too little starting material has been used. If pores are unoccupied for too long, this will lead to a more rapid decline in active pores.

4) Temperature curve:

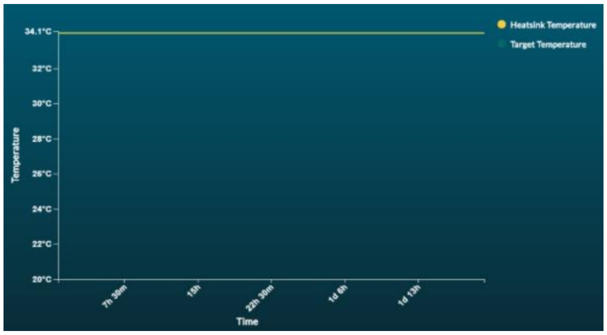

This graph shows the fluctuations in the temperature of the sequencer over time. The target temperature is indicated and any deviations from this line could indicate fluctuations of the ambient temperature. When deviations occur, check whether any factors could influence the temperature of the sequencer, e.g. if the sequencer is placed next to the air vents of the computer. Temperature deviations can sustainably influence sequencing performance.

5) Translocation speed:

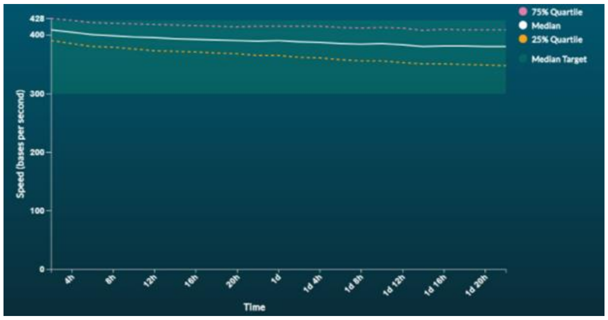

The translocation speed shows the performance of the flow cell. Ideally, the curve should stay within the green target range, however, the performance may decrease over time. A rapid drop in translocation speed may indicate that too much DNA has been loaded into the flow cell. When such a drop occurs, the flow cell can be refueled to increase the translocation speed. For this, refer to the Oxford nanopore homepage and the available protocols. When translocation speed is higher than the target, this might indicate that the temperature of the sequencer is too high. Again, check the surroundings for potential heat sources.

Bioinformatic analysis

General information:

For a detailed description on how to install the required programs, please refer to the separate manual that has been provided.

This protocol has been designed for the use in Ubuntu. Ubuntu is one of the forms of the operating system Unix that is widely used for the analysis of sequencing data and large datasets in general. Within this operating system, it is possible to work with and manipulate large datasets (e.g. reformat tables, modify the contents of datasets…) and to manipulate multiple files at once. To work within Ubuntu, it is necessary to learn a few commands, since in this working environment, commands are entered as text via the keyboard. A few of the most important commands are listed below, but for a more comprehensive introduction into working with the command line, it is advisable to work through one of the following tutorials:

http://www.ee.surrey.ac.uk/Teaching/Unix/unix1.html
https://ubuntu.com/tutorials/command-line-for-beginners#1-overview

Overview of commands:

Command	Meaning	Example
**cd**	Change to home directory	cd
**cd ..**	Change to parent directory	cd ..
**cd directory**	Change into specific directory	cd directory | cd ~/path/to/directory
**ls / ll**	Lists files and directories in current location	ls | ls directory | ls ~/path/to/directory
**cp**	Copies a file	cp file ../file |cp file ~/path/to/directory/.
**mv**	Moves or renames a file	mv oldfilename newfilename
**mkdir**	Make a directory	mkdir directoryname
**head**	Show the first 10 lines of a file	head file | head -n 20 file
**tail**	Show the last 10 lines of a file	tail file | tail -n 20 file
**less**	Shows content of a file	less file (press Q to exit)
**wc**	Word count. Counts the number of lines	wc file
**cat**	Concatenate files	cat file1 file2 file3 > newfile
**sed**	Stream editor. Parses and transforms text	sed 's/pattern/newpattern/' file > newfile
**rm**	Remove file	rm file
**rm -r**	Remove directory	rm -r directory

- For most commands, a help manual can be accessed by adding *-h* to the command
- The path to a location can be given as an absolute path starting in the home directory (~/path/to/directory) or as a path relative to the currently location (*../path/to/directory* or *path/to/directory*). For more details refer to the tutorials linked above.
- To redirect the output of a command from the standard output (display in command line) to a file, add > *filename* to your commands. For more details refer to the tutorials linked above.
- To interrupt a running command press [Strg] + [C]
- Files and directories that are deleted via the command line are irretrievably deleted.

Introduction into data types:

During sequencing, data is being saved as the change in electrical signal. This data is saved in FAST5 files, an Oxford nanopore specific data format. To work with this data, it has to be translated back into the actual nucleotide sequence. This step is called basecalling and the output is saved in FASTQ files.

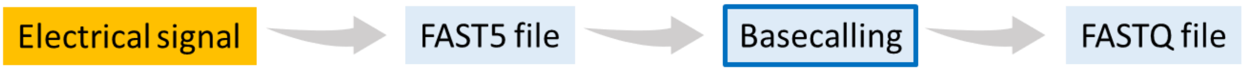

The FAST5 data files are not humanly readable. Instead we are dependent on the FASTQ data format.

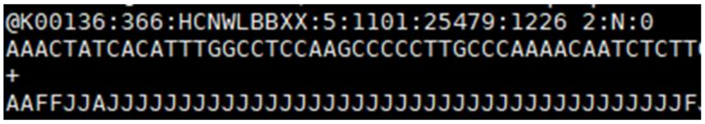

1) read identifier/header (always starts with @)
2) The nucleotide sequence
3) + (optional: again the ID)
4) base quality per nucleotide

FASTQ files all possess the same structure. The first line contains the so-called header, an identifier that contains information that are specific to the sequencer, with which the read was generated. This line always begins with "@". The second line contains the nucleotide sequence. The third line is in most cases uninformative and contains a "+" or a repetition of the header line. The fourth line contains the base quality information for each nucleotide. The algorithm, with which the base quality is measured is, like the header line, specific to each sequencing technology, but in general gives an estimation of the likelihood that the given base is accurate.

FASTA format:

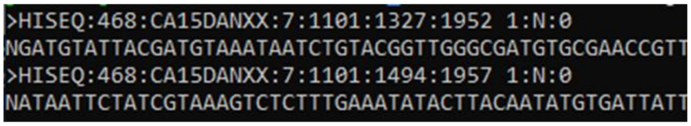

1) read identifier/header (always starts with >)
2) The nucleotide sequence
1) read identifier/header (always starts with >)
2) The nucleotide sequence

A second file type that will become important during this protocol is the FASTA format. FASTA files contain similar information as the FASTQ files, but here, the base quality information is left out and only the first two lines are given. The header in this case, starts with ">".

When using simultaneous basecalling with MinKNOW, files can be saved as both FAST5 and FASTQ files in the directory that has been chosen in the sequencing settings. During sequencing and basecalling, reads are sorted into different categories and folders.

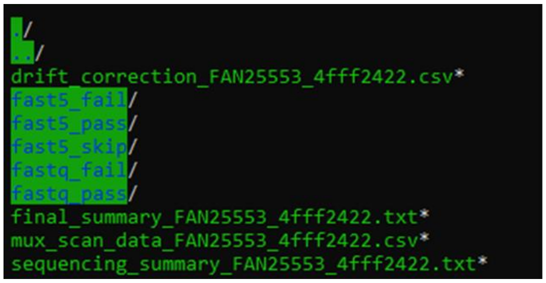

1) fast5 fail
2) fast5 pass
3) fast5 skip
4) fastq fail
5) fastq pass

Failed reads are reads that do not meet the specific nanopore quality criterions. Passed reads are reads that do meet those criteria. A third category, only present for FAST5 files is skip. This means reads have not been basecalled yet.

When using standard settings, reads will be split up into different files with 4000 reads per file.

Bioinformatic pipeline:

The aim of this bioinformatic pipeline is to identify the species identity of the individual mosquitoes that were sequenced. For this the following steps have to be taken:

- The sequence data has to be merged into a single file and additionally saved in FASTA format
- The read set has to be split up into the original individuals with the help of the individual identifiers
- The reads have to be error corrected. This is done in 2 different ways to ensure high accuracy.
- The resulting barcodes have to be BLASTed against a database to the identify the species status

The first step is to locate the folder, in which the sequence data have been saved. This is of course dependent on the individual file system and the sequencing settings that have been chosen.

It is however useful to know how to navigate from the Ubuntu home directory into the "normal" directory structure on your computer.

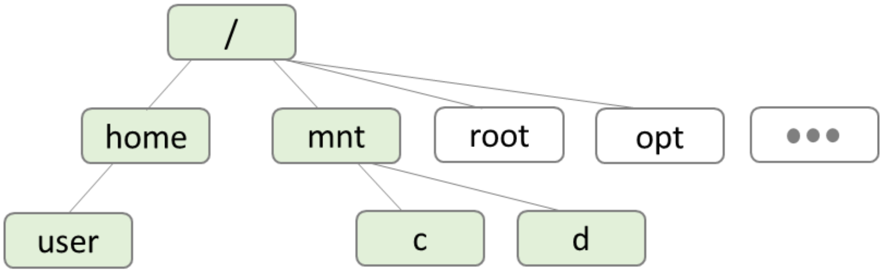

The starting directory of Ubuntu is the user directory, that is named after your individual username. When navigating from this directory to the standard Windows hard drives (e.g. c or d), the first step is to navigate two directories upwards, from there into the directory mnt, and then the hard drives and the normal file system is accessible.
The command to navigate from the starting directory into hard drive c would for example be:

Use the command: `cd ../../mnt/c` take care that between cd and .. is a space.

Merge sequence data into single file:
The reads that the bioinformatic analysis will be done with are the passed reads that are saved as FASTQ files. As a first step, all of the FASTQ files inside the fastq_pass folder need to be merged into a single file. The command that can be used for this is *cat*. For a usage example, refer to the command table. Normally, for this command, all files that are supposed to be concatenated need to be listed. Since during sequencing, a lot of files can be created, this would be too much work. We will thus tell the command, that we simply want to concatenate all FASTQ files inside the folder.

Use the command: `cat *.fastq > examplename.fastq`

The asterisk is a so-called wild card. In the case above, the command can be read as "concatenate all files that end with '.fastq' and save them as a new file that we name examplename.fastq".

Reformat FASTQ file to FASTA file:
For the bioinformatic analysis, the data are also required in FASTA format. As a next step, the FASTQ file will be reformatted to FASTA format. The following command should be written without line breaks.

Use the command: `sed -n ‘1~4s/^@/>/p;2~4p’ examplename.fastq > examplename.fasta`

Explanation of command:

```
sed ‘s/pattern/newpattern/’ file > newfile

1~4   ——→ split file into 4-line blocks, the following statement is true for line 1
s///  ——→ substitute the following pattern for a new pattern
^@/>  ——→ look for @ at the beginning of a line, exchange with >
p     ——→ now print this line with the modifications
2~4p  ——→ print the second line of the 4-line blocks
```

It is advisable to copy the files into a new folder where the analysis can be done safely, without the possibility of accidentally deleting the sequencing datasets. Refer to the commands table and the online tutorials if you need help with that.

<u>*Demultiplexing file:*</u>

To disentangle the read set and sort it into individual sequences (= *demultiplexing)*, a file is needed that contains information on how to identify the individuals. Specifically, this file includes sample IDs, the tag sequences, and the primer sequences. Below is an overview of the structure of the file:

SampleID	Tag F	Tag R	Primer F	Primer R
S1_COI	ATCCGGTCGGAGA	TTGCGTCTCACGC	GGTCAACAAATCATAAAGATATTGG	TAAACTTCAGGGTGACCAAAAAATCA
S2_COI	ATCCGGTCGGAGA	GCCGGTCCAAGTG	GGTCAACAAATCATAAAGATATTGG	TAAACTTCAGGGTGACCAAAAAATCA
S3_COI	ATCCGGTCGGAGA	CTGTCGAGGCGAC	GGTCAACAAATCATAAAGATATTGG	TAAACTTCAGGGTGACCAAAAAATCA
S4_COI	ATCCGGTCGGAGA	TCTACTGTTGTGC	GGTCAACAAATCATAAAGATATTGG	TAAACTTCAGGGTGACCAAAAAATCA
S5_COI	ATCCGGTCGGAGA	TGTATATTCAGCG	GGTCAACAAATCATAAAGATATTGG	TAAACTTCAGGGTGACCAAAAAATCA

This demultiplexing file, however, must be saved without column names and the different fields should be delimited with commas instead of tabs in order to be readable by the program that will be used.

S1_COI,ATCCGGTCGGAGA,TTGCGTCTCACGC,GGTCAACAAATCATAAAGATATTGG,TAAACTTCAGGGTGACCAAAAAATCA
S2_COI,ATCCGGTCGGAGA,GCCGGTCCAAGTG,GGTCAACAAATCATAAAGATATTGG,TAAACTTCAGGGTGACCAAAAAATCA
S3_COI,ATCCGGTCGGAGA,CTGTCGAGGCGAC,GGTCAACAAATCATAAAGATATTGG,TAAACTTCAGGGTGACCAAAAAATCA
S4_COI,ATCCGGTCGGAGA,TCTACTGTTGTGC,GGTCAACAAATCATAAAGATATTGG,TAAACTTCAGGGTGACCAAAAAATCA
S5_COI,ATCCGGTCGGAGA,TGTATATTCAGCG,GGTCAACAAATCATAAAGATATTGG,TAAACTTCAGGGTGACCAAAAAATCA

The demultiplex file can be created in Excel or any text editor and should be saved inside the analysis folder as a .csv file.

BLAST database:

For an error-correction step during the bioinformatic pipeline, barcodes will be searched in a database by using BLAST to correct for frameshift (see pipeline below for details). To create a BLAST database, it is possible to download FASTA sequences from the NCBI nucleotide database. Since species identification in this protocol concentrates on mosquitoes, the database can be reduced to this group of species.

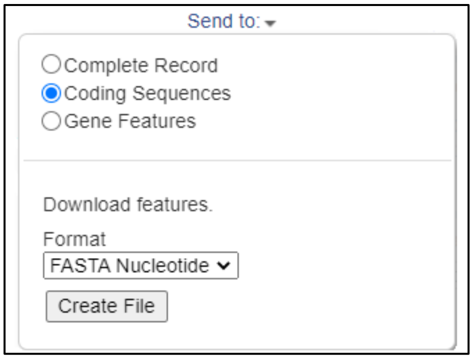

To search for nucleotide sequences, go to: https://www.ncbi.nlm.nih.gov/nuccore/ and search for: ((COI) AND "mosquitos"[porgn:__txid7157]). This will limit sequences to COI sequences of mosquitoes.

Download the search results as a FASTA file by choosing “Send to”, “Coding sequences”, and “FASTA nucleotide” as format and download the file by choosing “Create File”. The download may take a few minutes. Move the file into the analysis folder and create the BLAST database. The following command should be written without line breaks:

Use the command:

```
makeblastdb -in downloaded_sequences.fasta -dbtype
nucl -parse_seqids -blastdb_version 5 -title
mosquito_database -out mosquito_DB
```

This command will create several files that all start with “mosquito_DB”.

Overview of the pipeline:

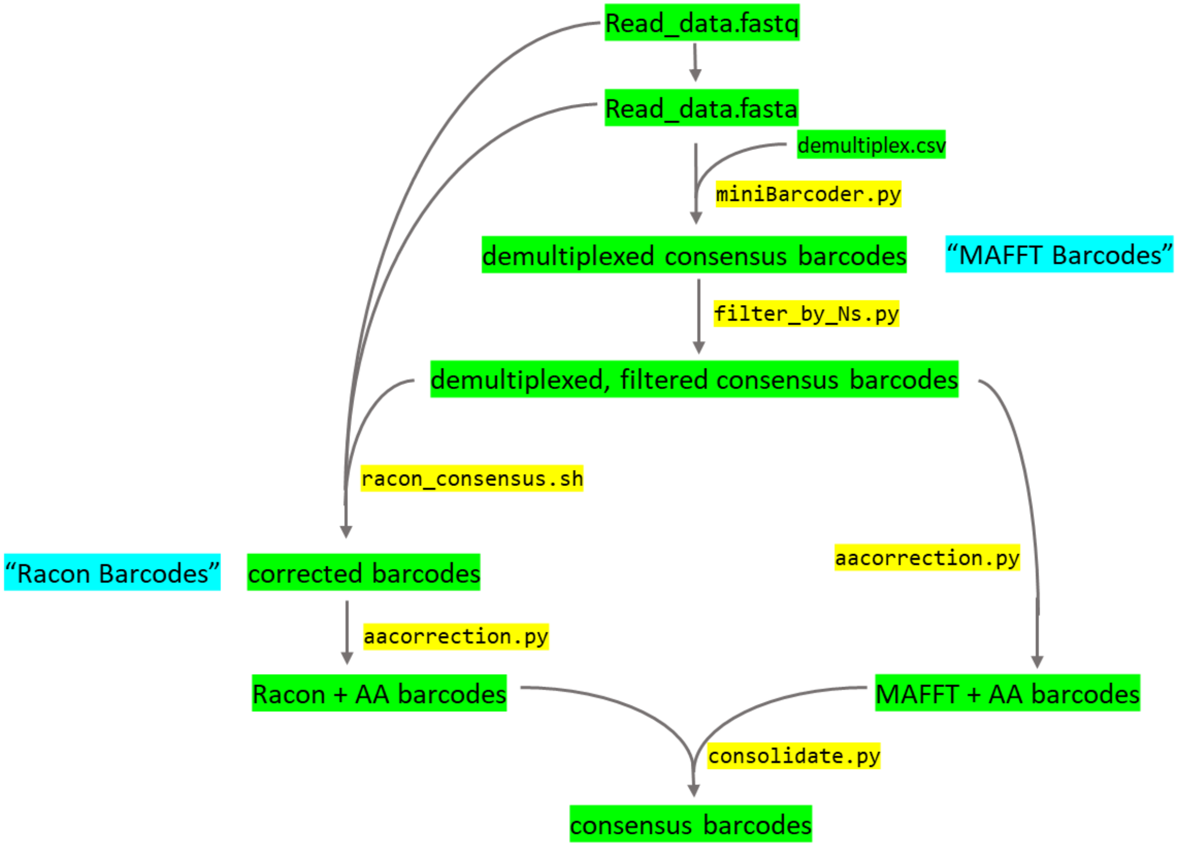

This overview shows the different steps of the pipeline. All programs and scripts that are being used are depicted in yellow, and the resulting data files are depicted in green.

The first step – the reformatting of the FASTQ file into a FASTA file – has already been done at this point. The next step is to demultiplex the read set with the help of the demultiplexing table and a script that is called miniBarcoder.py.
Afterwards, the individual reads will be filtered for ambiguous bases and the resulting data will be used for 2 different error correction steps.
The first error correction will be conducted with racon, a program widely used to assemble and correct sequencing reads. Afterwards, a second error correction step, the so-called amino-acid-correction (or aa-correction) will be conducted on the racon-corrected reads and on the read set of the previous step. Afterwards, both read sets will be merged into a single consensus barcode for each individual, which can then be used to identify the species.

Demultiplexing the read set:
At this step, it is necessary to activate the conda environment that has been installed previously (refer to the installation guideline in a different document).

Use the command: `conda activate mbconda`

To demultiplex the read set, the program miniBarcoder will be used. To receive information on the different parameters that can be adjusted, look at the help manual for this tool.

Use the command: `miniBarcoder -h`

For COI barcodes it should work fine to use the standard settings, and simply give a minimum length with the -l option. The following command should be written without the line break.

Use the command:
```
miniBarcoder -f exampledata.fasta -d demultiplex.csv -o
miniBarcoder_out -l 650
```

This command created a new directory inside the analysis folder called miniBarcoder_out. This folder contains several files, of which most are intermediate files that were created during the command, and that will not be used again. The most important file is *all_barcodes.fa* that contains the demultiplexed barcodes for each individual.

To make the remaining steps of the analysis easier, this file will be copied into the analysis folder and renamed to a more suitable name.

Use the command: `mv miniBarcoder_out/all_barcodes.fa MAFFT_barcodes.fa`

Filter reads for ambiguous sites:
Now, the newly created read set will be scanned for reads that include a lot of ambiguous positions (nucleotides that are not clearly identifiable). These positions are coded as N. The COI barcodes after removing the primer sequences, should be approximately 650 bases long. A good rule of thumb is to allow for 1% ambiguous sites in the sequence and filter out everything with more than 6 Ns.

Use the command: `filter_by_ns.py -i MAFFT_barcodes.fa -n 6`

This command automatically saved the output in a new file that is called MAFFT_barcodes_Nfilter.fa. These barcodes will now be used for the two different correction steps with Racon and the AA-correction script.

Racon error-correction:

As input for the error correction with Racon, several files are used as input (refer to overview of the pipeline). Racon will use the original read datasets (FASTA and FASTQ) and will compare those to the current barcodes and then use these to correct for any errors that are still present. Thus, the information that Racon needs are the FASTA and FASTQ read sets, the output folder of miniBarcoder and the current MAFFT-barcodes. Again, the following command should be written without line breaks.

Use the command: `racon_consensus.sh exampledata.fasta exampledata.fastq miniBarcoder_out MAFFT_barcodes.fa RACON_barcodes`

This command results in two types of output. The first one is a new file inside the analysis folder called *RACON_barcodes.fa* and a new folder with intermediate results called *RACON_barcodes*.

Amino-acid-correction:

The second correction step will be performed on the newly created Racon-corrected-barcodes and on the previous, uncorrected set of barcodes. The amino-acid-correction is aimed at targeting frame shifts, which are a common cause of error in MinION sequencing.

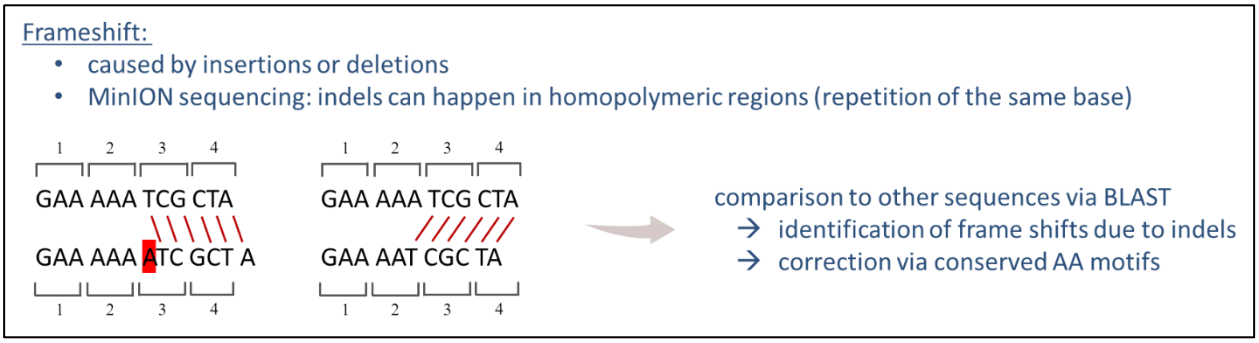

A known problem that causes a large proportion of the errors during MinION sequencing, are correctly calling the number of bases in homopolymeric regions (stretches of single nucleotide repeats). When a nucleotide passes through the nanopore, a change in electrical current is registered by the sequencer. However, when repeats of a single base pass through the pore, the sequencer has trouble in identifying the correct number of this base, as the signal stays constant over a certain period of time. This can then result in frameshifts in the resulting sequence.

The amino-acid-correction uses the fact that COI sequences, as mitochondrial sequences, are rather conserved, and frameshifts only happen very rarely. It is thus possible to compare the created barcodes to available sequences of closely related individuals to identify and correct any frameshifts. For this step, the BLAST database that was created on page 18 will be used. The following command should be used without line breaks.

Use the command: `aacorrection.py -b RACON_barcodes.fa -d mosquito_DB -o RACON_aacorr_barcodes_Nfilter.fa`

The same command will be used again for the dataset that has not been corrected with Racon.

Use the command: `aacorrection.py -b MAFFT_barcodes_Nfilter.fa -d mosquito_DB -o MAFFT_aacorr_barcodes_Nfilter.fa`

Merge the two different barcodes:

The last step of the pipeline is to consolidate the two different barcode sets. One of the sets has only been corrected with the Amino-acid-correction, and the other set has been corrected with Racon and with the Amino-acid-correction. The combination of both methods should have resulted in a high level of accuracy and the resulting barcodes should be highly reliable.

Use the command: `consolidate.py -m MAFFT_barcodes_Nfilter.fa -r RACON_aacorr_barcodes_Nfilter.fa -o consolidated_barcodes.fa -t temp_consol`

The resulting barcodes can now be used to identify the species that were found in the field. This is possible either via a local BLAST against a local database, or online on the NCBI BLAST homepage.

The manuals on how to perform a local BLAST search can be found here: https://www.ncbi.nlm.nih.gov/books/NBK279690/

Due to storage and data handling limitations, a database for only the most important group of organisms should be downloaded. NCBI makes several different kinds of databases available for download. One of them is the non-redundant invertebrate database, which is a (still substantial) subset of the overall BLAST database and which can be downloaded here: ftp://ftp.ncbi.nlm.nih.gov/refseq/release/invertebrate/

If the database should be limited to mosquitoes only, then it has to be assembled and curated independent of NCBI.
