# Supplementary material for: A barcoding pipeline for mosquito surveillance in Nepal, a biodiverse dengue-endemic country
Source: Parasit Vectors. 2022 Apr 24;15:145. doi: 10.1186/s13071-022-05255-1 (PMC9035287; doi:10.1186/s13071-022-05255-1)
Supplement: Supplementary file 3 — Additional file 3: Installation guideline for programs needed during the webinar. [file 13071_2022_5255_MOESM3_ESM.pdf]

Installation of Ubuntu and necessary programs

This installation guide will demonstrate you how to install and run a Windows subsystem for Linux, called Ubuntu, and install several programs that allow us to work with sequencing datasets. Linux is one of the different varieties of Unix, an operating system that is almost always used when it comes to working with sequencing datasets. It has its own special language that is called ‘bash’. Differently from Windows, where you can control everything with your mouse, here we only use our keyboard and type in commands. If you have never used bash before, after the installation of Ubuntu, I will list two homepages that explain a bit more and teach you the basic commands.

<u>Installation of Ubuntu</u>

- Go to Settings and klick on “Apps”
- under “Related settings” go to “Programs and Features”

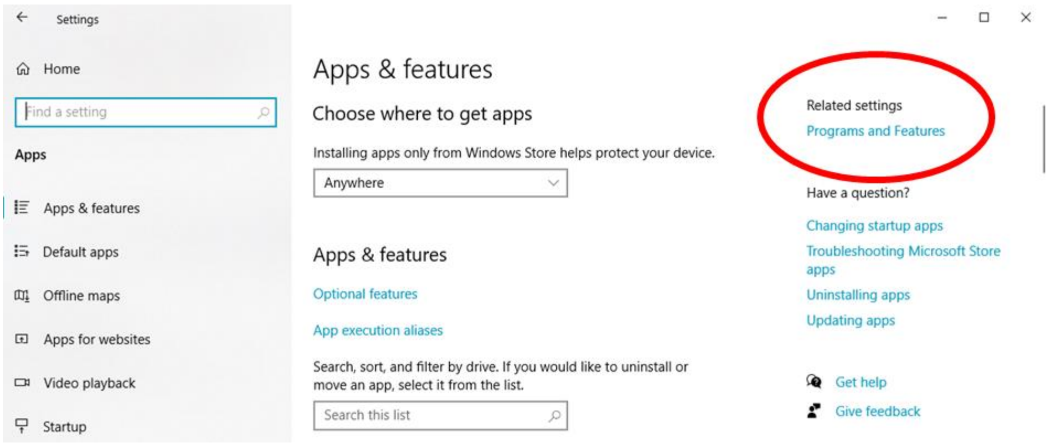

- Klick on “Turn Windows Features on or off”

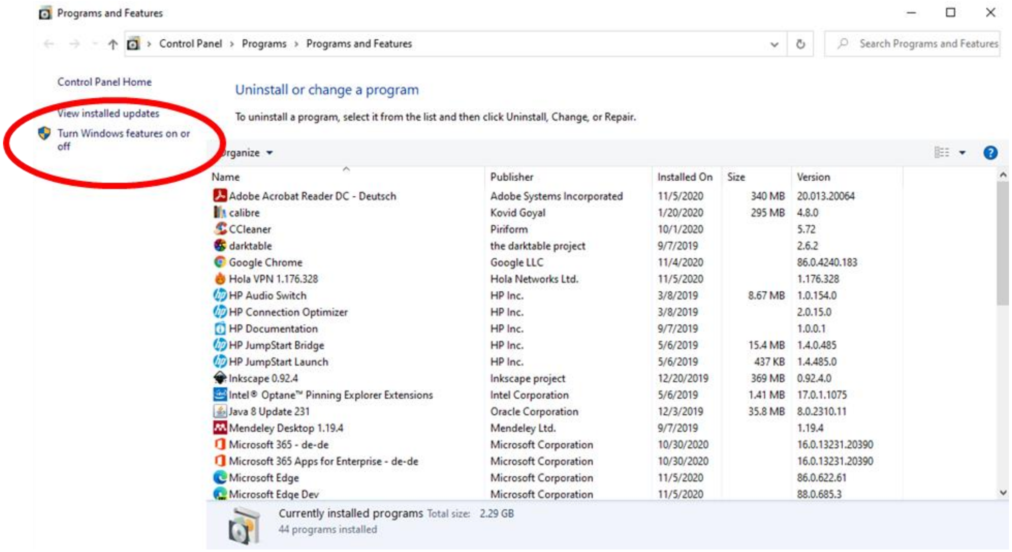

- A new window will open, scroll down the list, and tick the box at “Windows Subsystem for Linux”, after that klick on “OK”

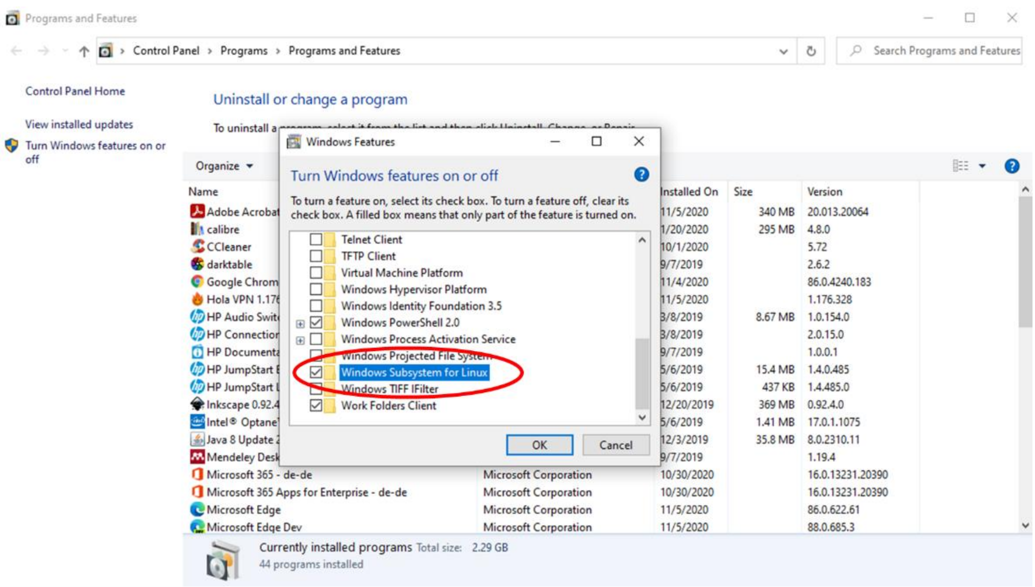

- After that the computer needs to be restarted. For that simply klick on "restart now"
- Next, when the computer has restarted, open the Microsoft Store (Windows Symbol -> either scroll through program list or type "Microsoft store")
- Type "Ubuntu" in the search field and chose the circled version

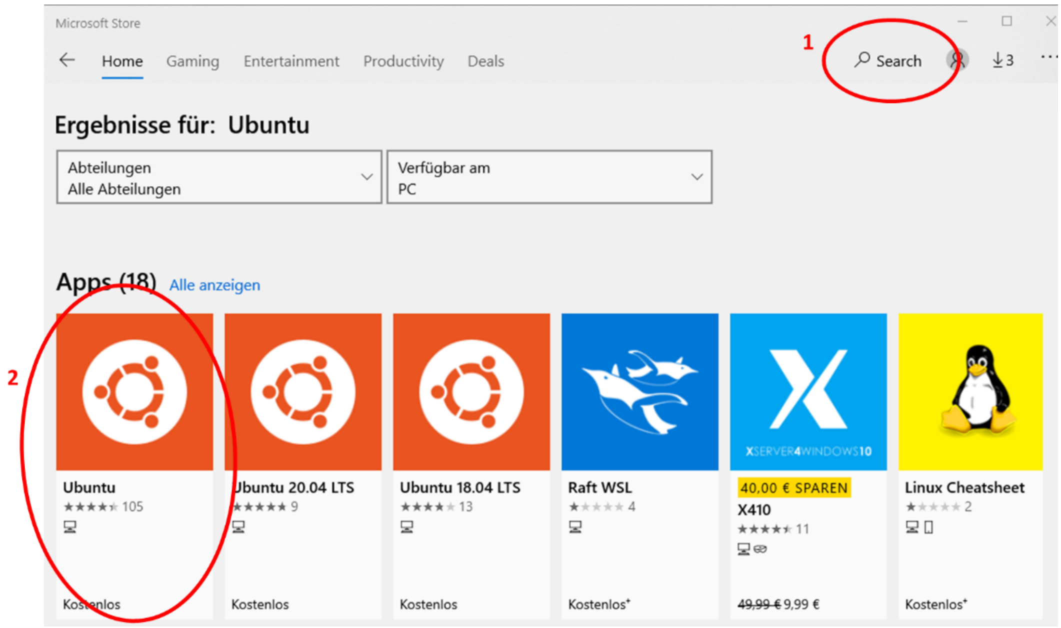

- Klick on "Download"
- Afterwards klick on "launch" to open the program
- The dark window that opens is called the "terminal". This is where we will work from now on.
- First, Ubuntu will finish the installation. Afterwards you can enter a *username* (I would recommend to use your name in small letters, without spaces) that you would like to use, and then set a *password* (this will not be shown in the terminal. Just type it, press Enter ↲, and repeat the step).
- As soon as a line appears with username@Computername in green, we can start working. In the terminal this should look like this:

```
jhartke@JANE: ~
  Memory usage:           77%
  Swap usage:             0%
  Processes:              7
  Users logged in:        0
  IPv4 address for wifi0: 192.168.2.112
  IPv6 address for wifi0: 2003:c9:2725:5691:5dc3:a50c:2c86:df66
  IPv6 address for wifi0: 2003:c9:2725:5691:5d8c:244c:22c0:2854

1 update can be installed immediately.
0 of these updates are security updates.
To see these additional updates run: apt list --upgradable


The list of available updates is more than a week old.
To check for new updates run: sudo apt update


This message is shown once once a day. To disable it please create the
/home/jhartke/.hushlogin file.
jhartke@JANE:~$
```

At this point, if you have no experience with bash, I strongly recommend going to one of the following homepages that explain a bit more and teach some basic commands that will help you to find your way around this tutorial more easily:
http://www.ee.surrey.ac.uk/Teaching/Unix/unix1.html
https://ubuntu.com/tutorials/command-line-for-beginners#1-overview

Just a few short tips:

- The tab ⭾ key on your keyboard is one of the most important keys when you work with bash. You can use it to complete words while you are writing. If you for example have a very long file name, you can type the first few characters and then press ⭾ and the complete name will appear, when the first part is unique
- Copying something in this Ubuntu version works with a right klick. Mark the part you want to copy with your mouse, klick right, and klick right again to insert it to the bottom line
- Find out where you are on your PC, by looking at the blue part of the bottom line (the command line). If you only see the ~ , it means you are in your starting directory.

Installation preparation:

General advice: This tutorial works best if you do all of the commands one after the other in one sitting. It reduces possible errors and also helps you to not get lost.

- As a first step we will create a folder called "bin", where we will install all programs
- For this type `mkdir bin` and press Enter ↵ (mkdir stands for "make directory", and "bin" is the name we chose)
- We can now test if the directory has been created by typing `ll` + ↵. (These are lower case "L" and not the number "1".) This command lists all subdirectories and files that are in our current directory. The results should look like this:

```
jhartke@JANE:~$ mkdir bin
jhartke@JANE:~$ ll
total 8
drwxr-xr-x 1 jhartke jhartke  512 Nov  5 13:01 ./
drwxr-xr-x 1 root    root     512 Nov  5 12:22 ../
-rw-r--r-- 1 jhartke jhartke  220 Nov  5 12:22 .bash_logout
-rw-r--r-- 1 jhartke jhartke 3771 Nov  5 12:22 .bashrc
drwxr-xr-x 1 jhartke jhartke  512 Nov  5 12:23 .landscape/
-rw-r--r-- 1 jhartke jhartke    0 Nov  5 12:23 .motd_shown
-rw-r--r-- 1 jhartke jhartke  807 Nov  5 12:22 .profile
drwxr-xr-x 1 jhartke jhartke  512 Nov  5 13:01 bin/
jhartke@JANE:~$
```

Folders appear in blue writing and files are white. As you can see, we now have a blue folder that is called bin.

- Now, we will change our directory and go into the newly created directory “bin”. For this we simply type `cd bin` + ↵. The command “cd” means “change directory”. You can check if it works by looking at the blue part of your command line that should now say “~/bin”.
- We can take a look around again with `ll` + ↵ and we will see that the directory is empty.
- We will change that now by installing programs in here.

<u>Installation of Miniconda:</u>

- First, we will install Miniconda, a program that includes a number of useful packages that we will need. It also makes the installation of other programs easier.
- The program can be found at: https://docs.conda.io/en/latest/miniconda.html
- Scroll down to “Linux installers” and choose the version with “Python 2.7” and “Miniconda2 Linux 64-bit”. Right klick on the link and then chose “Copy link address”

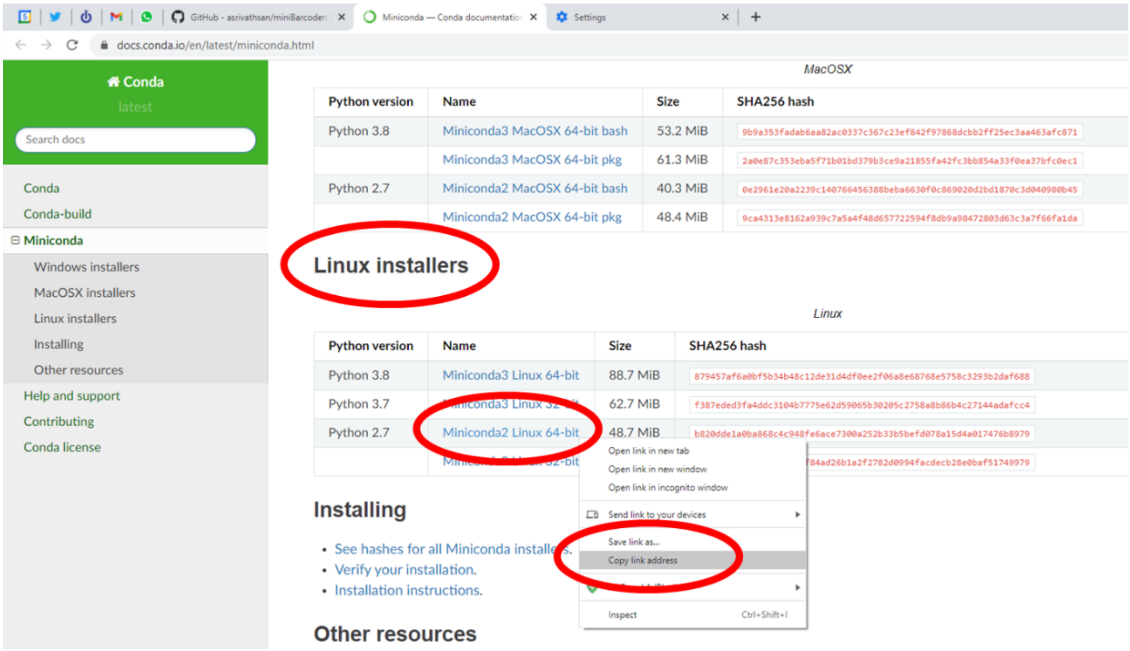

- Go back to the terminal, type **wget** and then do a right klick to copy the link into the terminal (**make sure there is a <u>space</u> between wget and the link**). Press Enter, and

```
jhartke@JANE:~/bin$ wget https://repo.anaconda.com/miniconda/Miniconda2-latest-Linux-x86_64.sh
--2020-11-05 13:57:01--  https://repo.anaconda.com/miniconda/Miniconda2-latest-Linux-x86_64.sh
Resolving repo.anaconda.com (repo.anaconda.com)... 2606:4700::6810:8303, 2606:4700::6810:8203, 104.16.131.3,
...
Connecting to repo.anaconda.com (repo.anaconda.com)|2606:4700::6810:8303|:443... connected.
HTTP request sent, awaiting response... 200 OK
Length: 51093759 (49M) [application/x-sh]
Saving to: 'Miniconda2-latest-Linux-x86_64.sh'

Miniconda2-latest-Linux-x86 100%[=======================================>]  48.73M  6.74MB/s    in 7.9s

2020-11-05 13:57:14 (6.15 MB/s) - 'Miniconda2-latest-Linux-x86_64.sh' saved [51093759/51093759]

jhartke@JANE:~/bin$ ll
total 49920
drwxr-xr-x 1 jhartke jhartke      512 Nov  5 13:57 ./
drwxr-xr-x 1 jhartke jhartke      512 Nov  5 13:01 ../
-rw-r--r-- 1 jhartke jhartke 51093759 Jun 16 22:04 Miniconda2-latest-Linux-x86_64.sh
jhartke@JANE:~/bin$ bash Miniconda2-latest-Linux-x86_64.sh
```

  the program download should start. If you encounter a problem here, try `wget` *`link`* `--no-check-certificate`.
- When it is finished, type in the next command **`bash Miniconda2-latest-Linux-x86_64.sh`** + ↵ The last part is the name of the downloaded file. You can simply copy it from the terminal (e.g. from the download link or after using **`ll`** + ↵) or use the tab key ⭾ to autocomplete, to make sure everything is typed correctly
- Miniconda will then ask you to read and approve the license agreement. For this, press Enter ↵. The agreement will start, and you have to continue to press Enter ↵ to read it. At the end, the program will ask you to accept it by typing **yes** + ↵. Do this and press Enter ↵ again.
- Now, give the <u>correct location,</u> where Miniconda should be installed! We want it to be in the new <u>"bin" folder</u> that we created, so we are not happy with the directory that Miniconda proposes. We will therefore change it! Type **`/home/username/bin/miniconda2`** and press Enter ↵.

```
Miniconda2 will now be installed into this location:
/home/jhartke/miniconda2

  - Press ENTER to confirm the location
  - Press CTRL-C to abort the installation
  - Or specify a different location below

[/home/jhartke/miniconda2] >>> /home/jhartke/bin/miniconda2
```

- When the installation is finished, Miniconda will ask if you want to start Miniconda. We will say **no** + ↵ for now.

<u>Modification of .bashrc:</u>

- Now we will use a text editor to change a text file that controls a bit of our Ubuntu system and its programs. This file is called ".bashrc" and the Ubuntu System is constantly looking at this file for information about the location of programs. We will now give it information about the newly installed program Miniconda. For this, we need to go into our starting directory, meaning we leave the bin directory. We can do this with **`cd ..`** + ↵. This simply means "change directory and go one directory up".

- Type **`ll`** + ↵ to show which directories are present in our starting directory. You should see the following list, including a file named ".bashrc". This is the one we will edit.

```
jhartke@JANE:~/bin$ cd ..
jhartke@JANE:~$ ll
total 8
drwxr-xr-x 1 jhartke jhartke  512 Nov  5 15:11 ./
drwxr-xr-x 1 root    root     512 Nov  5 12:22 ../
-rw-r--r-- 1 jhartke jhartke  220 Nov  5 12:22 .bash_logout
-rw-r--r-- 1 jhartke jhartke 3831 Nov  5 15:11 .bashrc
drwx------ 1 jhartke jhartke  512 Nov  5 15:00 .cache/
drwxr-xr-x 1 jhartke jhartke  512 Nov  5 14:10 .conda/
drwxr-xr-x 1 jhartke jhartke  512 Nov  5 12:23 .landscape/
drwxr-xr-x 1 jhartke jhartke  512 Nov  5 15:05 .local/
-rw-r--r-- 1 jhartke jhartke    0 Nov  5 12:23 .motd_shown
-rw-r--r-- 1 jhartke jhartke  807 Nov  5 12:22 .profile
-rw-r--r-- 1 jhartke jhartke    0 Nov  5 14:29 .sudo_as_admin_successful
drwxr-xr-x 1 jhartke jhartke  512 Nov  5 14:09 bin/
jhartke@JANE:~$ nano .bashrc
```

- We will use the text editor *nano* to modify the .bashrc file. Type **`nano .bashrc`** and press Enter ↵. This editor is a command line editor. This means we have to control it with our keyboard. An overview of the different commands we can use, can be found on the bottom. The "^" symbol in this case stands for the key "Ctrl".
- We will create a shortcut to the location of the Miniconda program. Right now, to use Miniconda, we would always have to enter the complete path to where Miniconda is saved: /home/username/bin/miniconda2/bin/**conda**, which is a lot of typing. We will tell Ubuntu where exactly Miniconda is saved, so we can simply type "conda" and Ubuntu knows where to look for it.
- Under "#for examples" type:
  `#gedit`
  `export PATH=$PATH:/home/username/bin/miniconda2/bin`

```
  GNU nano 4.8                          .bashrc
# ~/.bashrc: executed by bash(1) for non-login shells.
# see /usr/share/doc/bash/examples/startup-files (in the package bash-doc)
# for examples

#gedit
export PATH=$PATH:/home/jhartke/bin/miniconda2/bin/

# If not running interactively, don't do anything
case $- in
    *i*) ;;
      *) return;;

^G Get Help   ^O Write Out  ^W Where Is   ^K Cut Text   ^J Justify    ^C Cur Pos    M-U Undo      M-A Mark Text
^X Exit       ^R Read File  ^\ Replace    ^U Paste Text ^T To Spell   ^_ Go To Line M-E Redo      M-6 Copy Text
```

- Then press "CTRL"+"O" and Enter ↵ to save, and "CTRL"+"X" ↵ to leave the editor.
- Now we have to tell Ubuntu that we want to use the updated version of the .bashrc file. We use the command **`source .bashrc`** + ↵ for this. Done.

Installation of MiniBarcoder:

- We will now start with the installation of the next program: MiniBarcoder
- First, go back into the "bin" directory: **`cd bin`** + ↵
- We now use Miniconda to create a working environment for the new program. This basically means that we create our own bubble inside Ubuntu, with specific programs

and program versions. The advantage of this is that this bubble will always stay the same, so when we enter the bubble, the program will always work.
The command we use for this is **`conda config --add channels bioconda`** + ↵

- Afterwards we use the command **`conda create -n mbconda python=2.7 mafft racon graphmap=0.5.2 blast seqtk git fasta3`** + ↵. This will tell Miniconda which programs we would like to use in our new environment (mafft, racon, blast, seqtk, git and fasta3), that we want to use python version 2.7 and graphmap version 0.5.2, and that we want to call this environment "mbconda".
The installation will start, and at some point, ask you if you would like to proceed with the installation of some programs. Say **y** + ↵ and it will continue.
- When this is done, we can enter the Miniconda environment by typing **`conda activate mbconda`** + ↵. If you get the message that this did not work use **`source activate mbconda`** + ↵ when entering the environment for the first time. The next time you want to activate mbconda, the first command should work as well.
- You can check if you are in the Miniconda environment by looking at the command line. In front of your username "(mbconda)" should appear.

```
jhartke@JANE:~/bin$ conda activate mbconda
(mbconda) jhartke@JANE:~/bin$
```

- We will now install one last program in here: Biopython. **`conda install -c anaconda biopython`** + ↵. When asked if you want to continue say **y** + ↵
- Now, we can install Minibarcoder.
- Make sure that you are still in the "bin" directory by checking the blue part of the command line that should say ~/bin. If so, we first download the program from Github with:
`git clone https://github.com/asrivathsan/miniBarcoder/` + ↵
If you should get an error message like the following, this can be due to your firewall or proxy server

```
(mbconda) jhartke@JANE:~/bin$ git clone https://github.com/asrivathsan/miniBarcoder/
Cloning into 'miniBarcoder'...
fatal: unable to access 'https://github.com/asrivathsan/miniBarcoder/': Failed to connect to github.com port 443: Connec
tion refused
(mbconda) jhartke@JANE:~/bin$
```

If this is the case, we can work around this by following the steps in green. If you did not have the problem and the download worked for you, you can skip the green part.

- Go to the homepage of minibarcoder (https://github.com/asrivathsan/miniBarcoder) and simply download it, like you would normally download a program. Go to the green field "Code" and chose "Download ZIP"

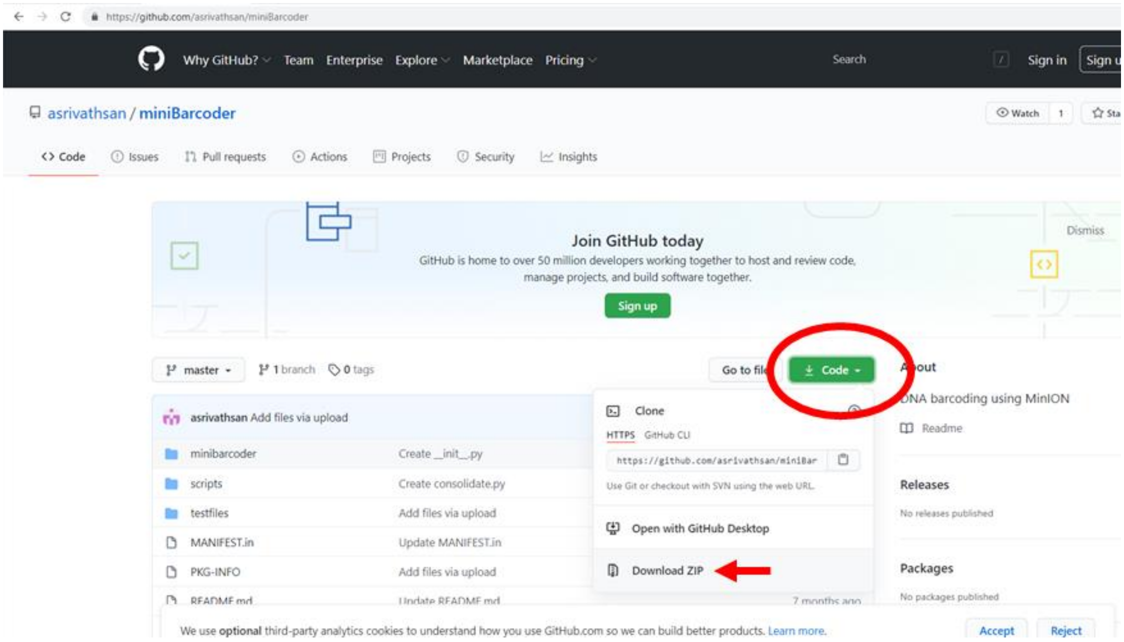

- afterwards, go into your downloads folder and unzip the minibarcoder folder.
- Now we need to copy it into our Ubuntu environment into the bin directory, where we actually want it to be. We do this back in our Ubuntu window. Make sure you are still in the bin folder.

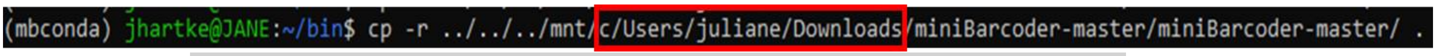

`cp -r ../../../mnt/c/Users/juliane/Downloads/miniBarcoder-master/miniBarcoder-master/ .` + ↲
The part marked in red is the path to your download folder. You will have to adapt it to your own computer. Most of the time, it should be enough to adjust the username from 'juliane' to your own computer username. In some cases also the path can be different (e.g. there is only one "miniBarcoder-master" folder)
The command `cp` stands for copy. The `-r` option tells it to copy all subdirectories within the folder, and the `.` at the end tells the command that we want to copy it to the folder where we currently are.

- Check if the folder is there by typing `ll` + ↲. We will now rename the folder to make it a bit shorter: `mv miniBarcoder-master/ miniBarcoder` + ↲

- Now we should all have a miniBarcoder directory in our bin folder and can continue at the same point.
- At this point, please check if you are still in the miniconda environment that we have created (check whether there is a white part of text with "(mbconda)" in front of your username in the command line). If this is not the case, enter the environment again with `conda activate mbconda` + ↲
- We go into the minibarcoder folder with `cd miniBarcoder` + ↲ and finish the installation with `python setup.py install` + ↲

Modifying .bashrc:

We will now do modify the control textfile again in the same way as before for miniconda.

- To modify the .bashrc file move back into our start directory **cd**. Open the .bashrc file again with nano .bashrc and enter the following lines.

  #Minibarcoder
  export PATH=$PATH:/home/username/bin/miniBarcoder
  alias miniBarcoder='python /home/username/bin/miniBarcoder/miniBarcoder.py'

- Do not forget to use your own username! In my .bashrc, it looks like this:

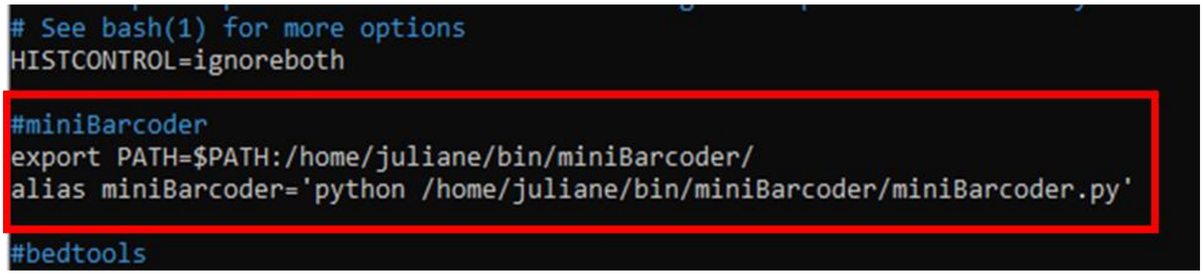

- Then press "CTRL"+"O" and Enter ↲ to save, and "CTRL"+"X" ↲ to leave the editor.
- Now we have to tell Ubuntu again that we want to use the updated version of the .bashrc file with `source .bashrc` + ↲ . Done.

Testing miniBarcoder:

Now we are almost done! We just need to test if the installation worked correctly by trying out a few commands. These commands test the different components of the miniBarcoder program and we will discuss the commands in detail during the webinar. MiniBarcoder luckily provides a few testfiles, which we can use for this.

For this part, again, make sure, that you are in the mbconda environment (check if the "(mbconda)" part is in front of your command line), if not, enter with **`conda activate mbconda`**.

- Go into the miniBarcoder directory and then go into the directory with the testfiles. We can do this in one command by using `cd bin/miniBarcoder/testfiles` + ↲, and then try out the commands below one after the other. The important part here is that there are no error messages when you try out the commands. A few of them might take a few minutes. Only continue with the next command when the first command is done (which is the case when your command line appears again):

```
mb_parallel_demultiplex.py -d demfile_2.txt -l 600 -o testout -f test.fasta

mb_parallel_consensus.py -i testout

mv testout/all_barcodes.fa test_mafft_barcode.fa

filter_by_Ns.py -n 6 -i test_mafft_barcode.fa

racon_consensus.py -i testout -d racon_out -o test_racon.fa -b test_mafft_barcode_Nfilter.fa
```

```
aacorrection.py -b test_mafft_barcode_Nfilter.fa -bo
test_barcodes_megablast.txt -bf test_barcodes_megablast.fasta -o
test_mafft_barcode_aacorr.fa
```

```
aacorrection.py -b test_racon.fa -bo test_barcodes_megablast.txt -bf
test_barcodes_megablast.fasta -o test_racon_barcode_aacorr.fa
```

```
consolidate.py -m test_mafft_barcode_aacorr.fa -r
test_racon_barcode_aacorr.fa -t con_temp -o test_consolidate.fa
```

On some computers, there are problems with the installation of one part of miniBarcoder, which is why some commands might not work. If you experience trouble with the test commands try out the following:

```
cd /home/username/bin
```

```
git clone --recursive https://github.com/isovic/racon.git racon
```

```
cd racon
```

```
mkdir build
```

```
cd build
```

```
cmake -DCMAKE_BUILD_TYPE=Release ..
```

```
make
```

```
cp build/bin/racon ~/miniconda2/envs/mbconda/bin/racon
```

After this, try out the test commands again. Don't forget to navigate back into the "testfile" directory again: `cd ~/bin/miniBarcoder/testfiles`

Now you are done with the preparations for the webinar! To exit Ubuntu, simply close the window.
