# Supplementary material for: A barcoding pipeline for mosquito surveillance in Nepal, a biodiverse dengue-endemic country
Source: Parasit Vectors. 2022 Apr 24;15:145. doi: 10.1186/s13071-022-05255-1 (PMC9035287; doi:10.1186/s13071-022-05255-1)
Supplement: Supplementary file 4 — Additional file 4: Teaching material of the webinar. [file 13071_2022_5255_MOESM4_ESM.pdf]

Molecular techniques of vector identification

Establishment of a field sequencing protocol for species identification

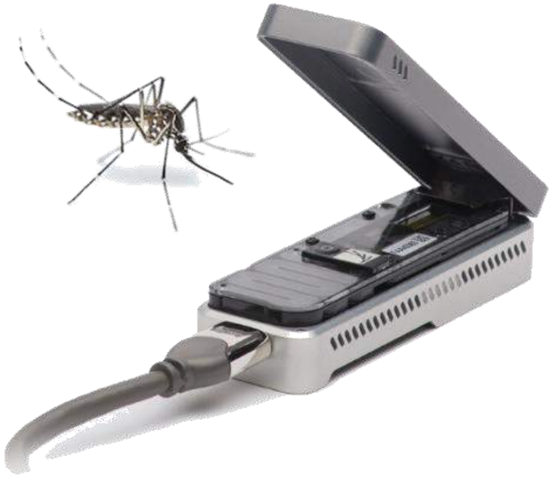

Vector-borne diseases

- Result from an infection that was transmitted to us by a living organism
- Account for 17% of all infectious diseases
- > 700.000 deaths every year
- Largest part due to mosquitoes

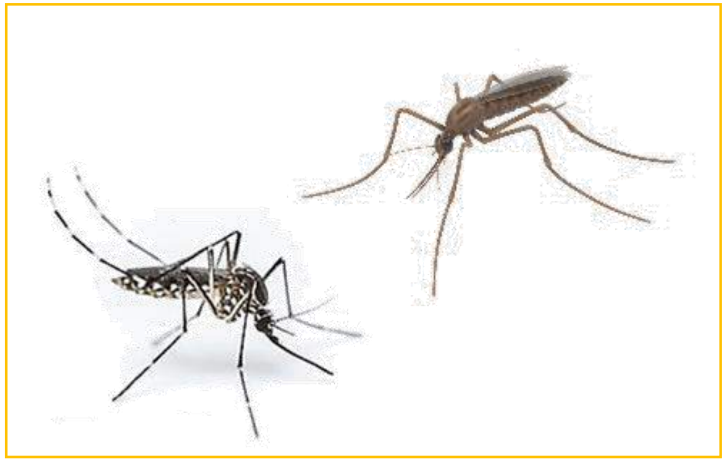

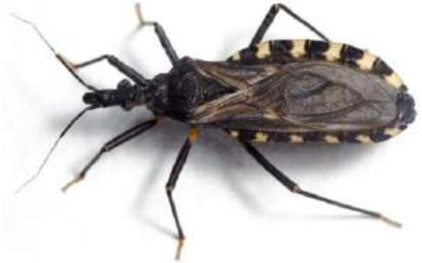

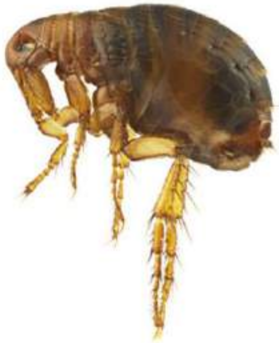

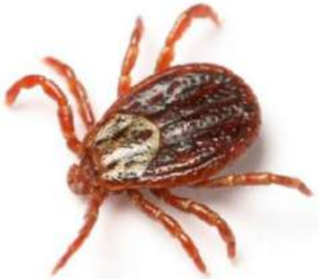

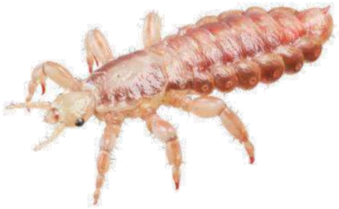

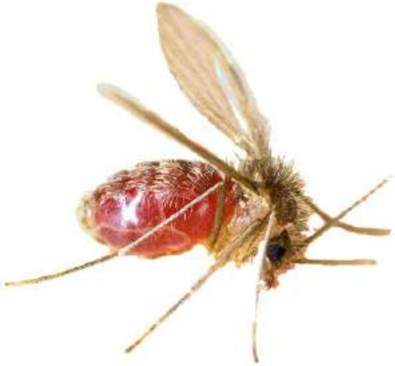

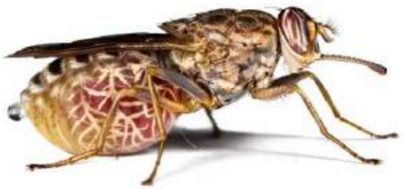

Sources:
WHO

Mosquito-borne diseases

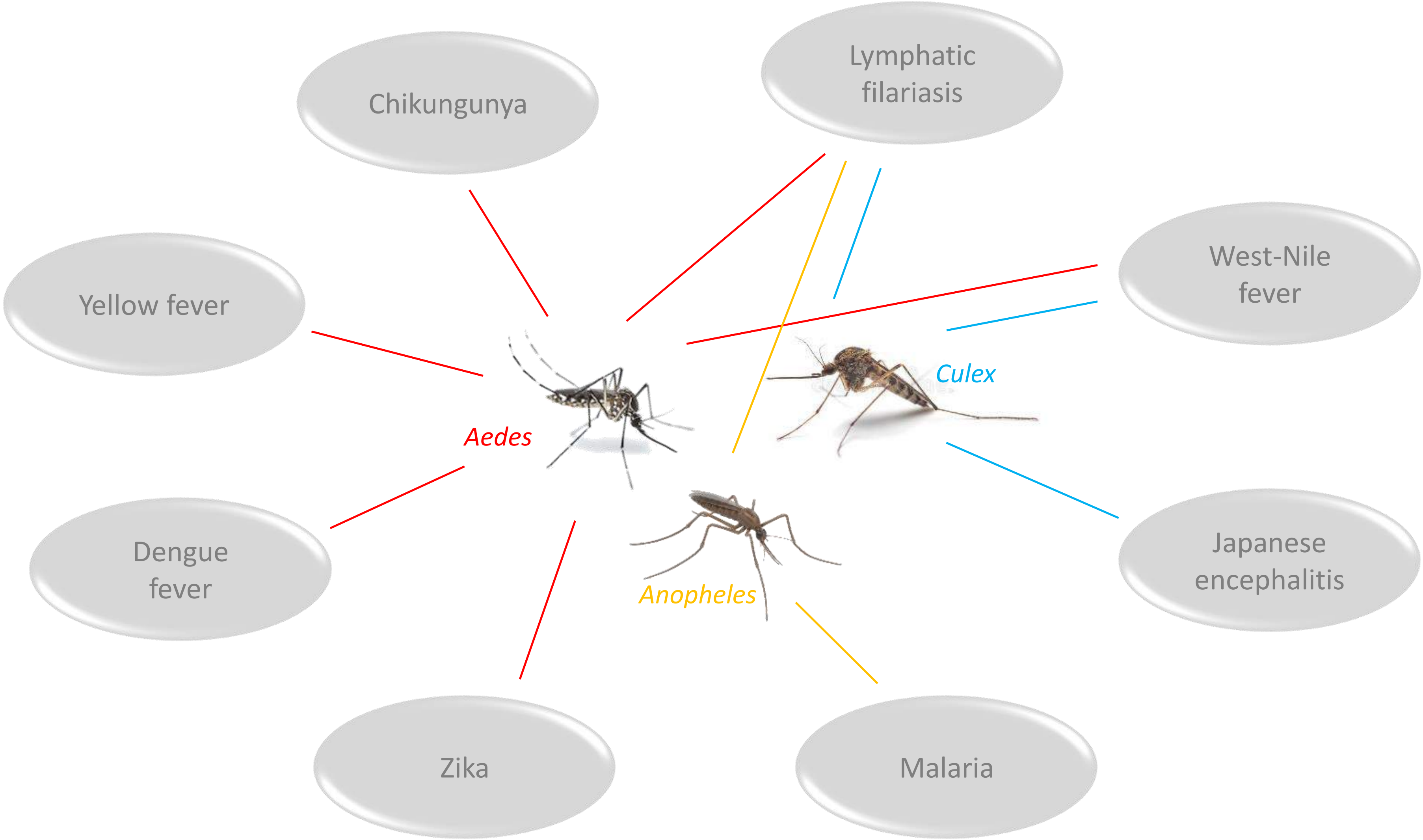

Sources:
Paneru 2019; Journal of Health and Allied Sciences

Mosquito-borne diseases

- **Malaria**: ~ 219.000.000 cases, ~ 400.000 deaths per year
- **Yellow fever**: ~ 200.000 cases, ~ 30.000 deaths each year
- **Chikungunya**: numerous epidemics (e.g. 1.3 million cases in 2015 in the Caribbean)
- **Dengue**
  - 100-400 million infections each year
  - 3.9 billion people at risk of infection
  - Largest number of reported cases was in 2019
  - Spread into non-endemic regions due to globalization and climate change

Sources:
WHO and CDC

Mosquito-borne diseases

Dengue cases 2019 [1]

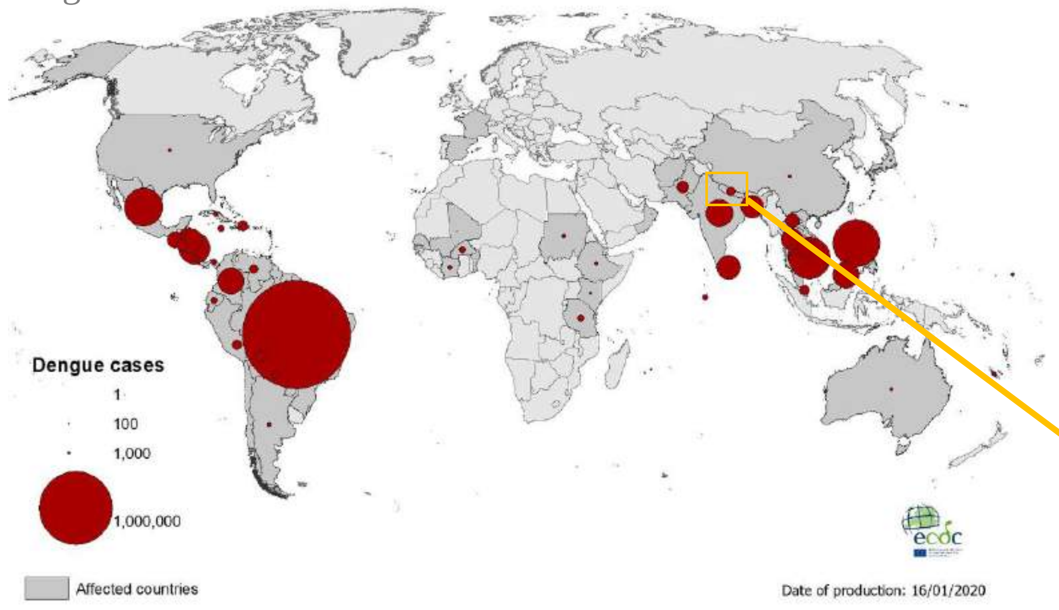

History of Dengue in Nepal

- First case reported in 2004 [1]
- First significant outbreak in 2006 (35 cases) [2]
- Numbers increasing each year
- Major outbreak in 2019 with < 14.000 cases [4]
- Transmission in Kathmandu (1.400 m Elevation) [4]

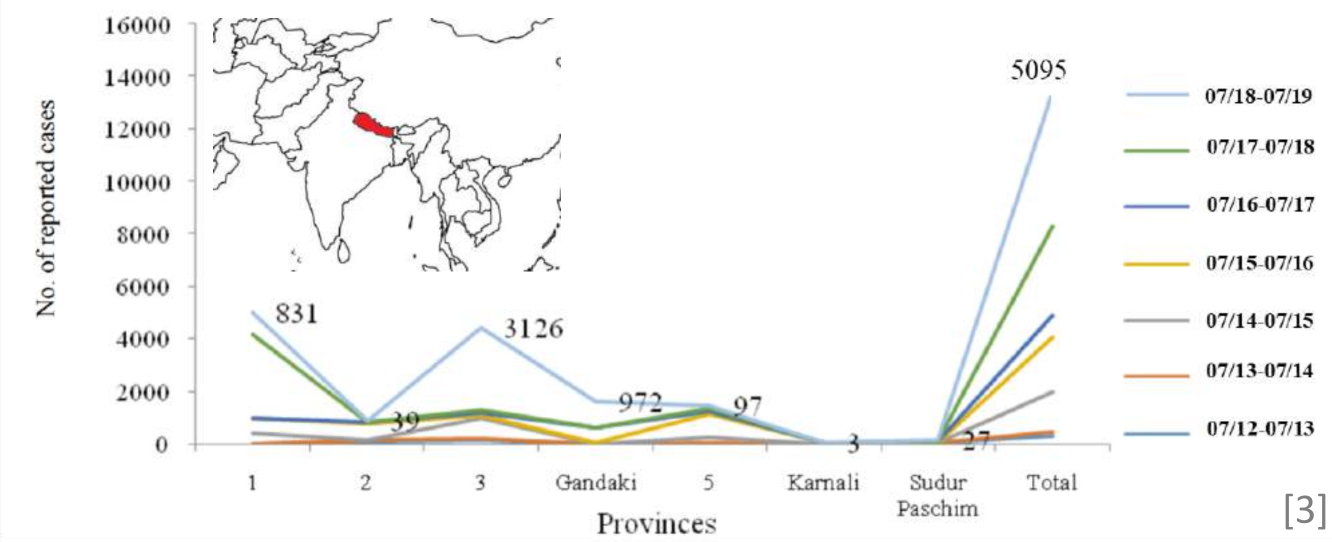

[3]

Sources:
[1] European Centre for Disease Prevention and Control
[2] Khetan et al. *2018*
[3] Paneru 2019; Journal of Health and Allied Sciences
[4] Adhikari & Subedi 2020

EntoCAP project - Overview

- **Aims:**
  - **Entomological training**
    - Build entomological capacity
    - Training of 300 health science students and medical doctors
  - **Training children**
    - Generate community interest in VBD
    - Train children in vector biology and control in Kathmandu
  - **Build entomological reference collection**
    - Natural History Museum in Kathmandu
    - New morphological reference collection
  - **Genetic barcodes as addition to morphological collection**
    - Establish field sequencing
    - Archive of information on the diversity of insects

↓

- Spread awareness of mosquito-borne diseases in Nepal
- Biological information about risk
- Helps physicians to correctly assess symptoms

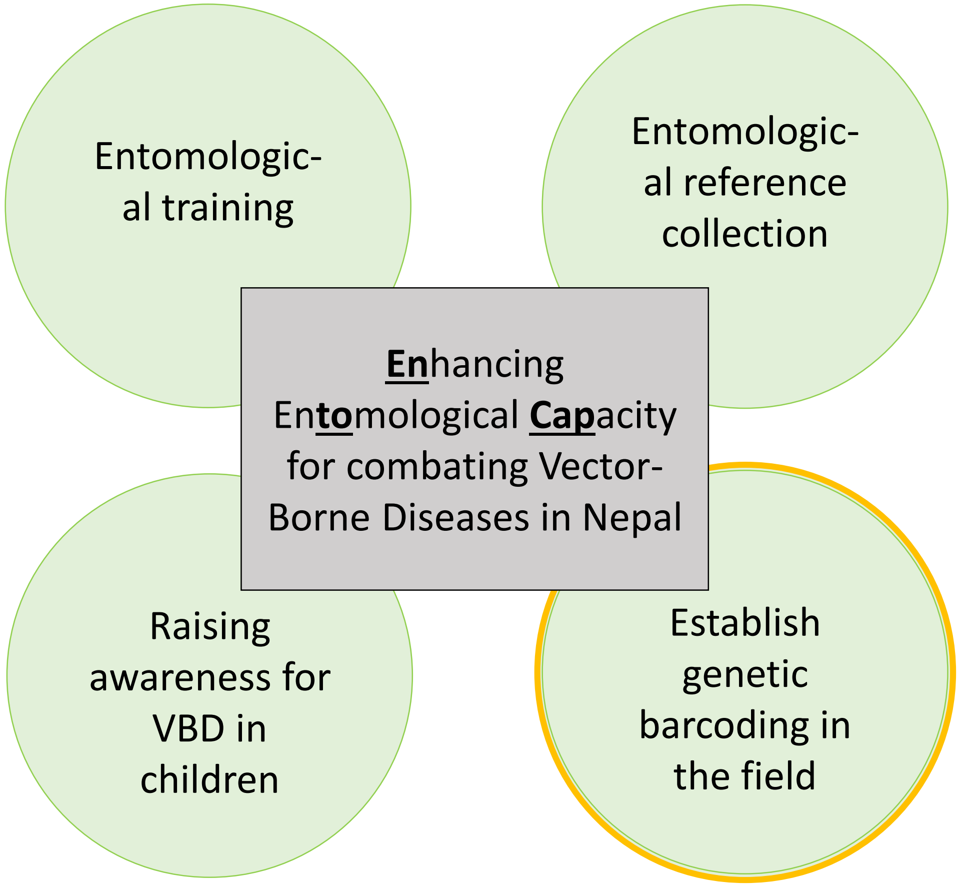

EntoCAP project – MinION: Goal

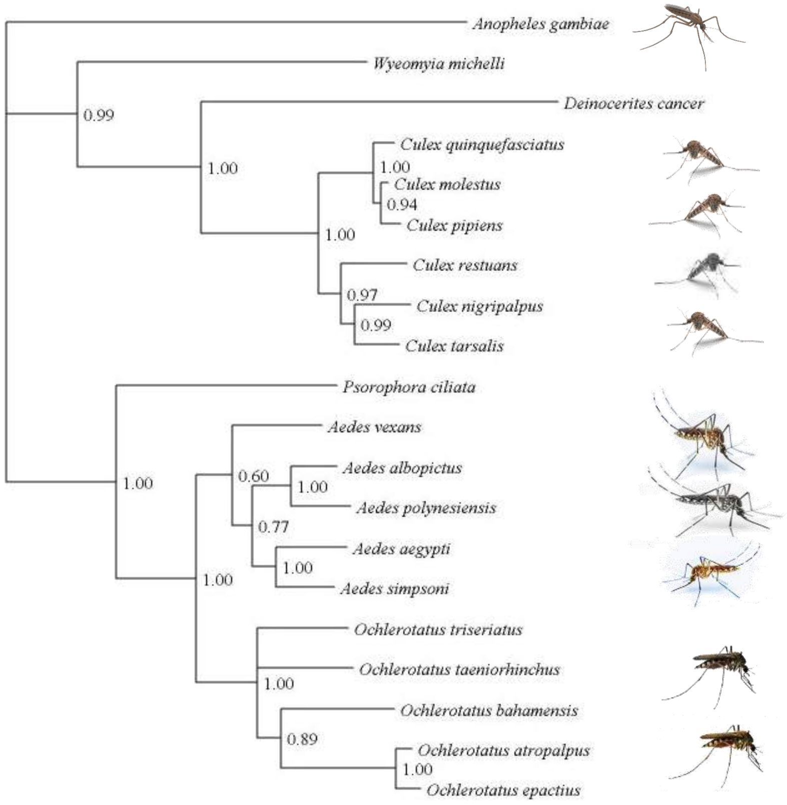

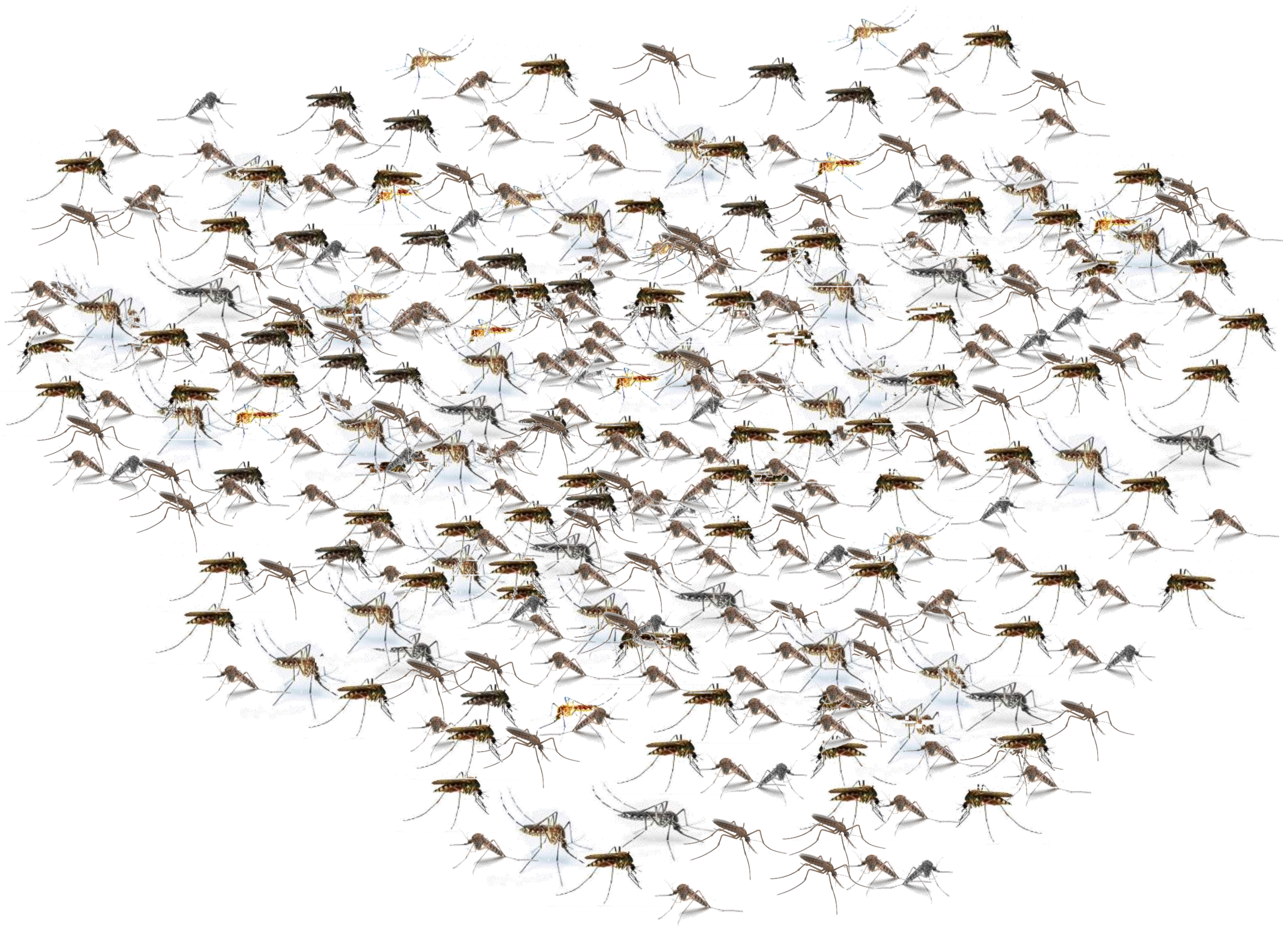

EntoCAP project – MinION: Goal

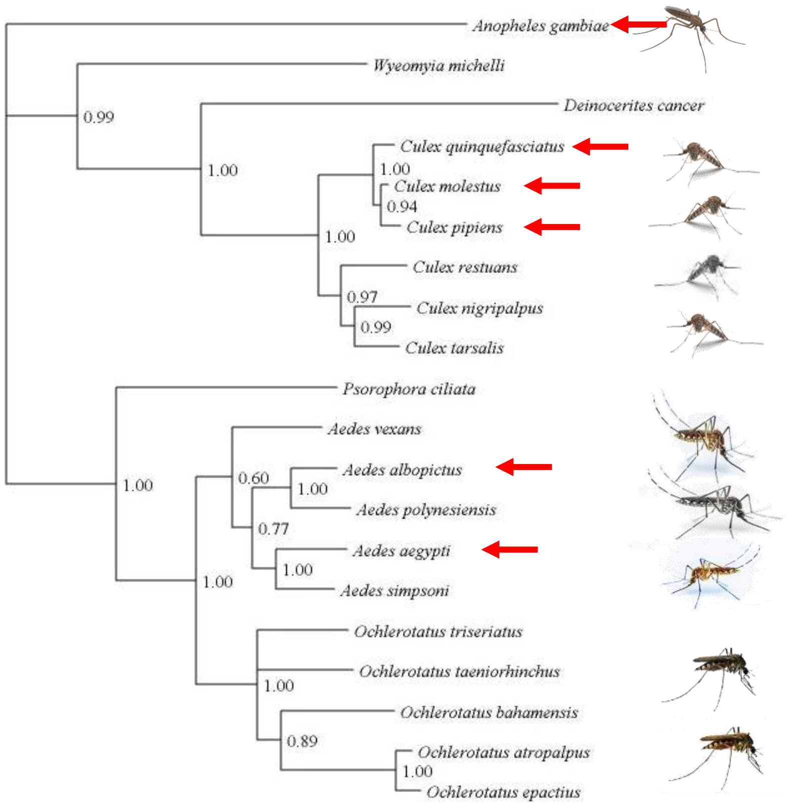

- Which species are occurring in a region?
- Which kind of diseases are they able to transmit?

- Assessing the risk of a disease outbreak
- More time to prepare!
  - Educate hospital staff and doctors
  - Educate population
  - Mosquito control

Genetic barcoding

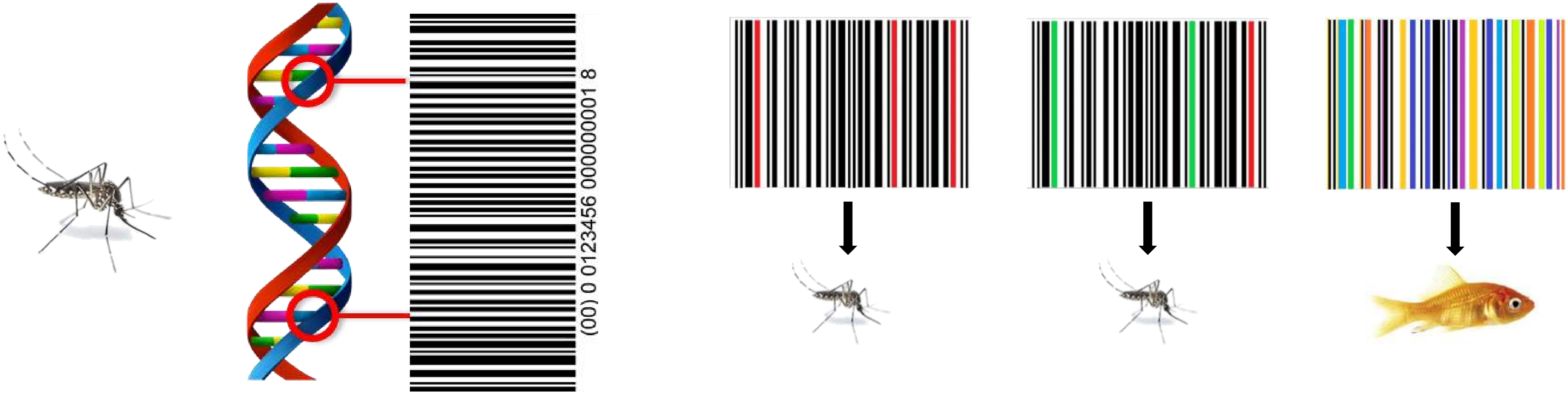

- Targets sequences in DNA that show variability between species
- Identification of species based on reference database

EntoCAP project

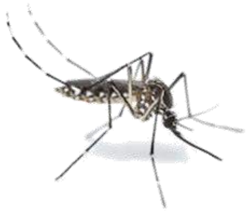

?

How do we get from a mosquito to information about its species?

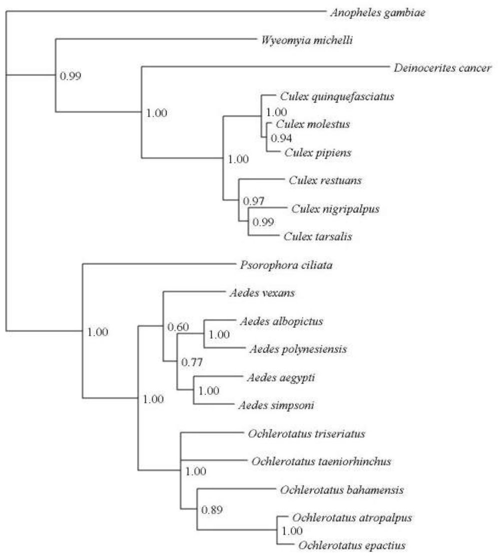

EntoCAP project

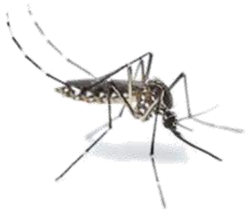

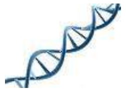

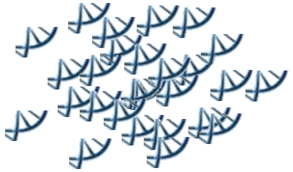

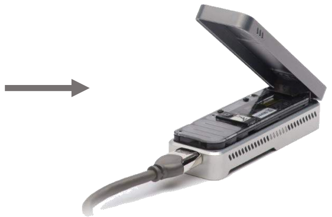

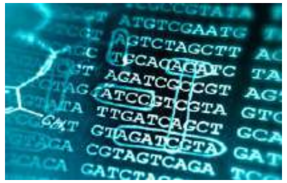

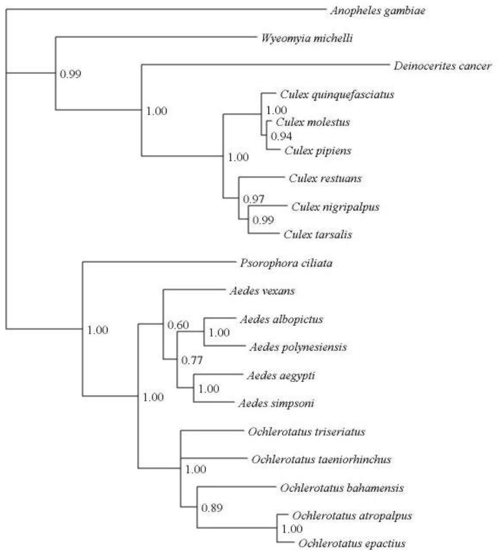

1 DNA Isolation

2 DNA Amplification

3 Sequencing

4 Bioinformatic Analysis

EntoCAP project

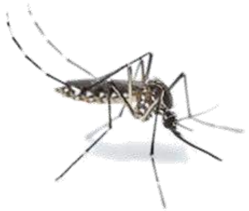

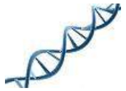

1

DNA Isolati[illegible]

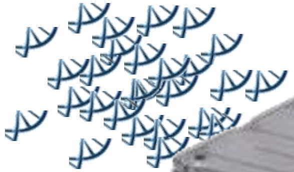

[illegible]

[illegible]plification

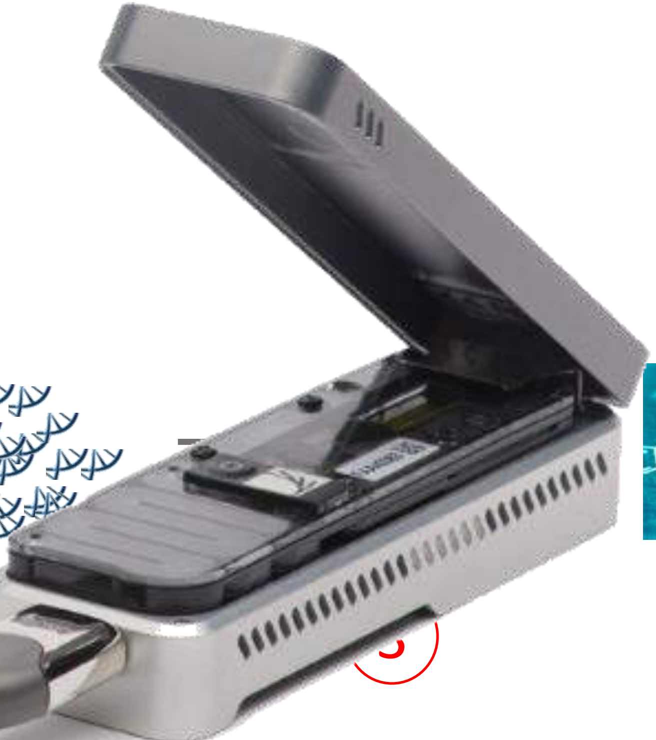

[illegible]

Sequencing

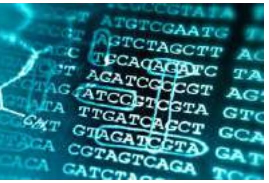

4

Bioinformatic Analysis

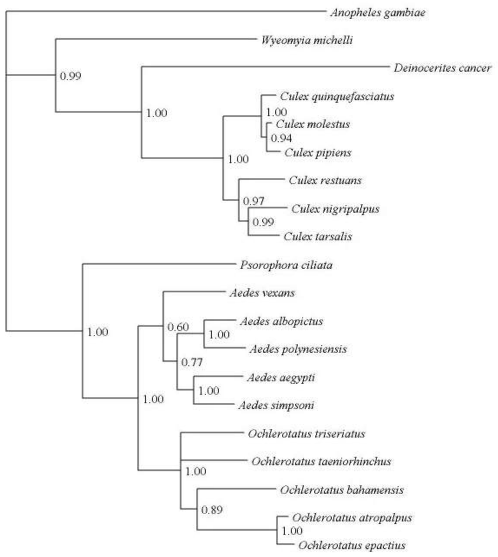

MinION sequencer

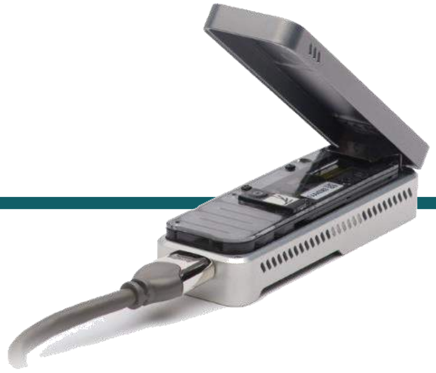

- Relatively easy to use
- Relatively low cost
- Can be used in field settings
- Variety of applications
  - Whole genomes, targeted sequencing, transcriptomes, metagenomics….
  - portable devices to high throughput

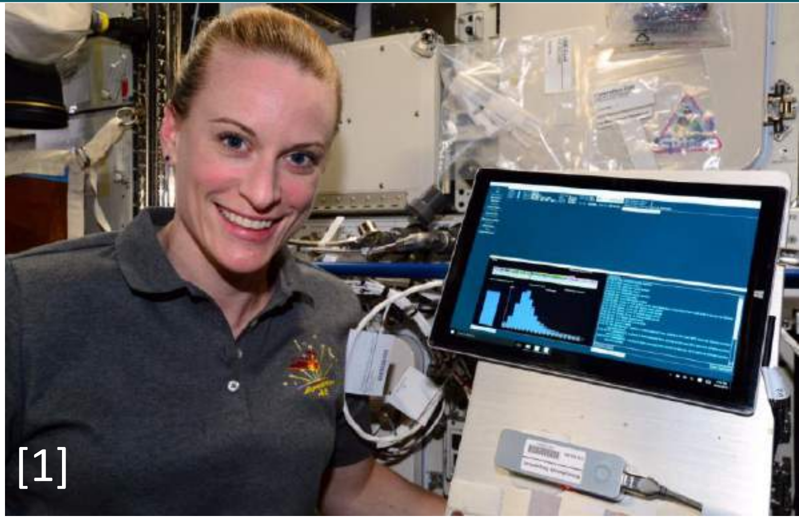
[1]

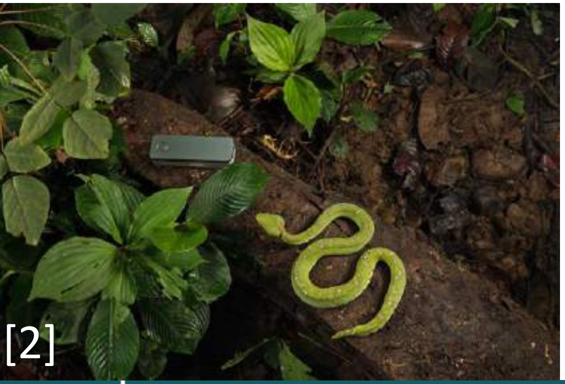
[2]

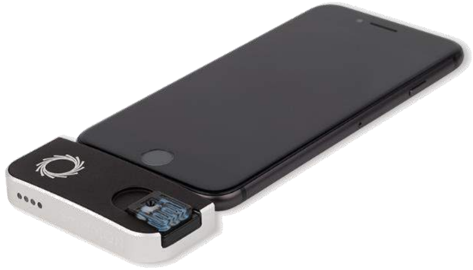

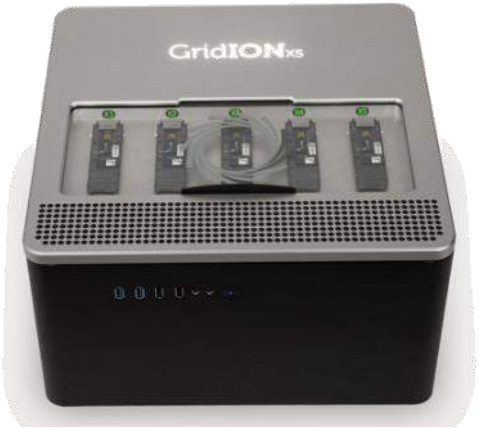

Sources:
[1] nasa.gov
[2] Pomerantz et al. 2018

MinION sequencer

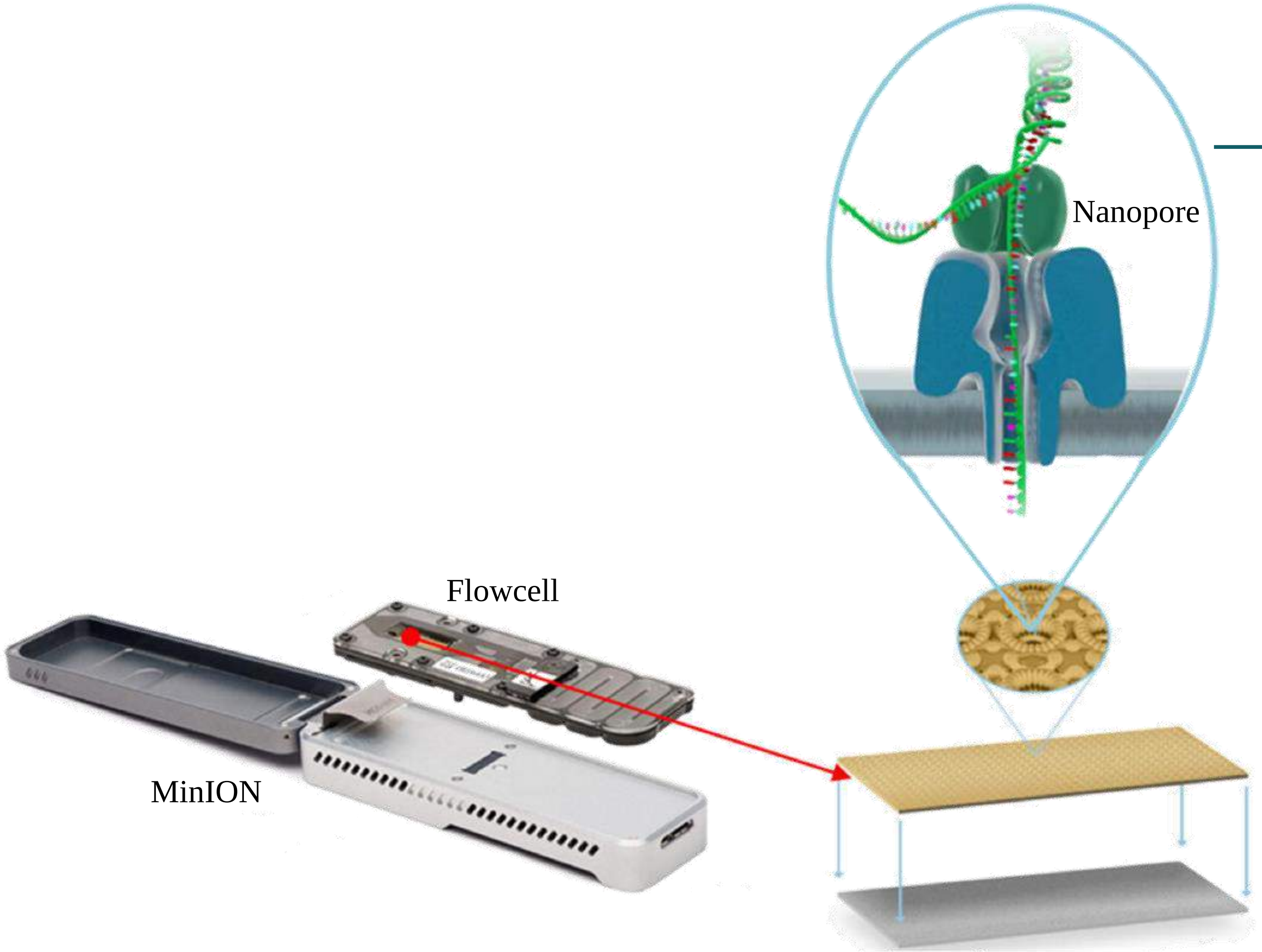

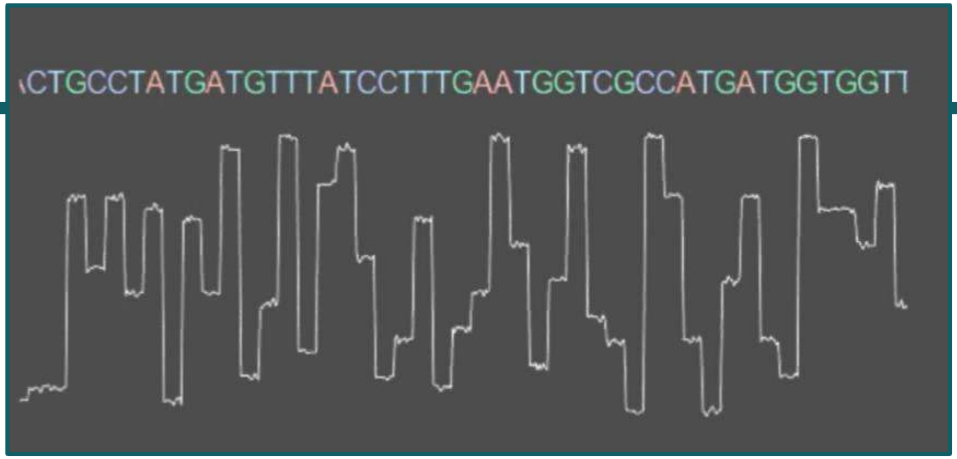

- Flowcell with nanopores
- DNA bases are identified by their change of electrical current
- This technology comes with advantages and disadvantages
  - Pro: length distribution of output reflects length distribution of input DNA
  - Contra: high error rate compared to other sequencing techniques

Sources:
[1] Oxford Nanopore
[2] Lu et al. *2016*

MinION sequencer – Error rate

- Error rate between 5-20% (dependent on types of molecules and library preparation)

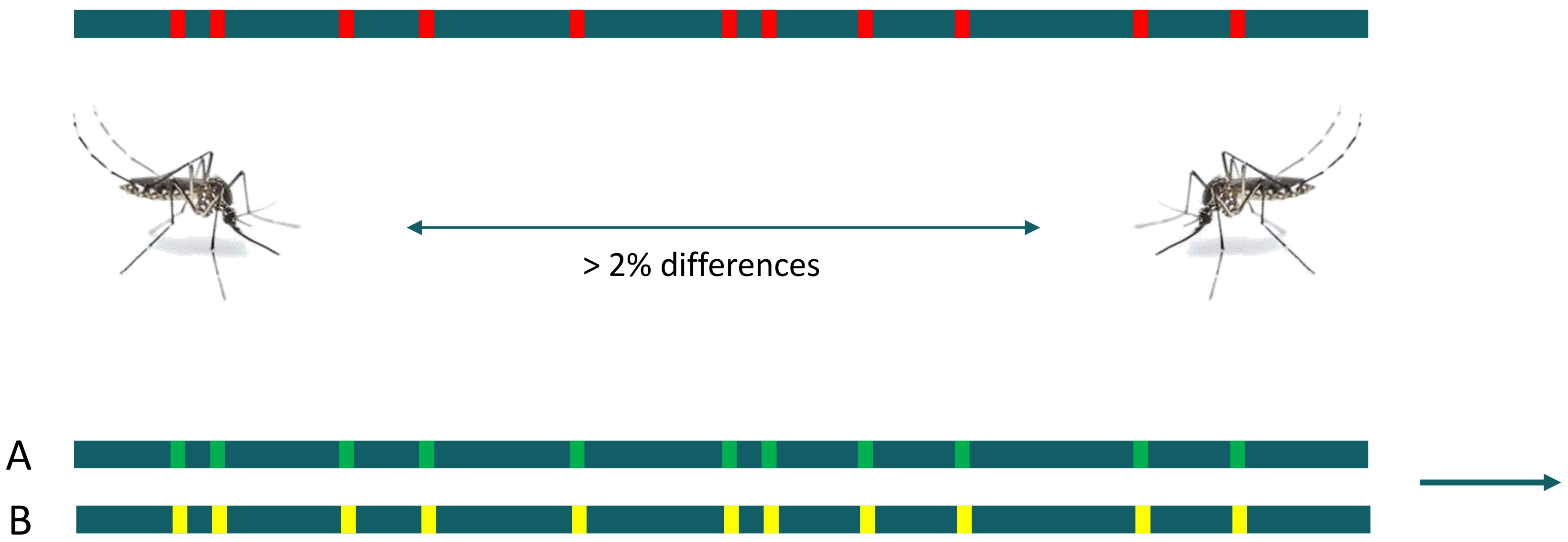

- Different species?
- High error rate?

How can we reliably identify species identity with the MinION?

MinION sequencer – Error rate

- We first need to take sequences from the same individual and group them together
- Then we can look for the "consensus" sequence
- After that we can compare it to other individuals or identify the species

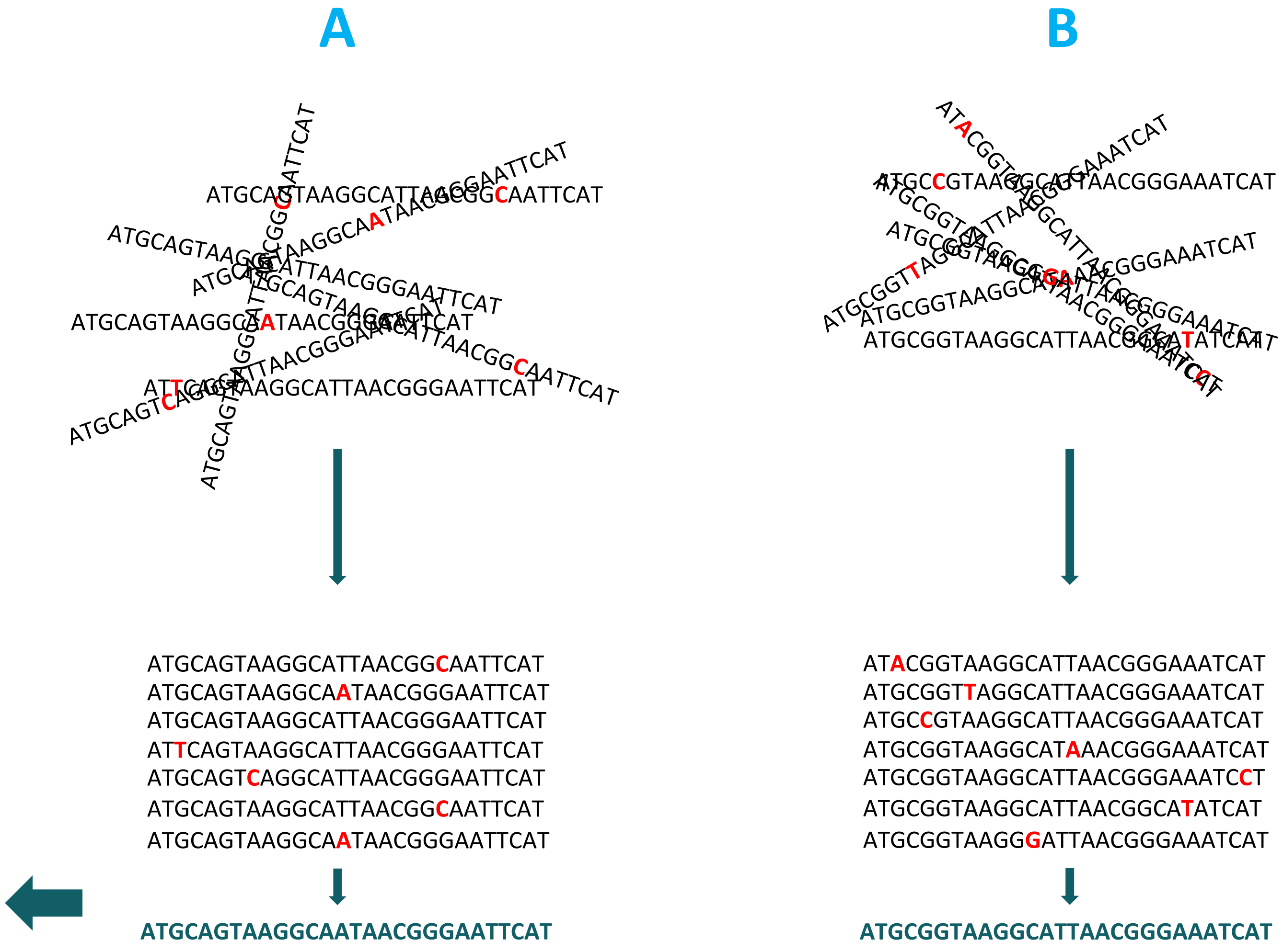

A ATGCAGTAAGGCAATAACGGGAATTCAT

B ATGCGGTAAGGCATTAACGGGAAATCAT

MinION sequencer – Error rate

How do we separate the sequences of different individuals?

A

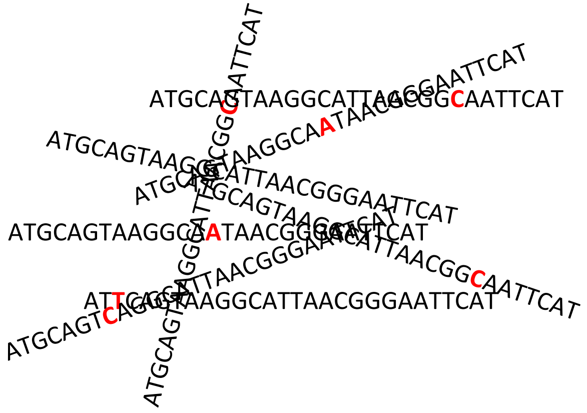

B

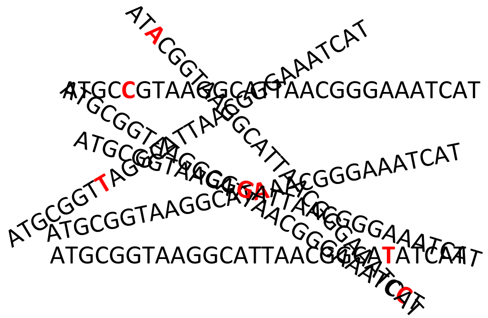

a) We sequence each individual separately

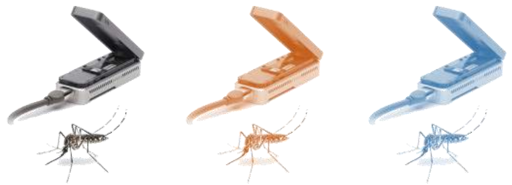

- Lot of work!
- Super expensive

b) We separate them after sequencing

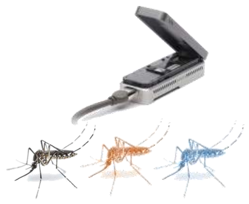

How?

PCR strategy

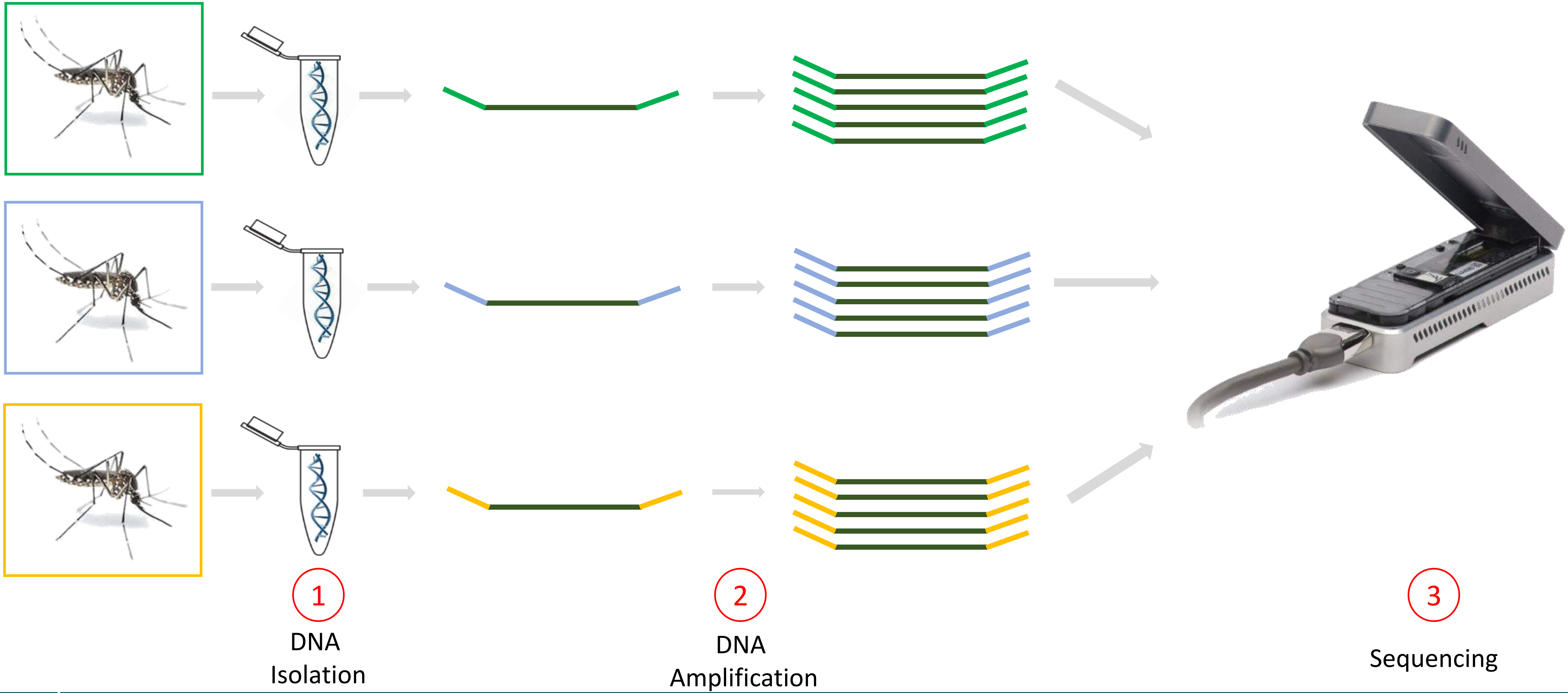

PCR strategy

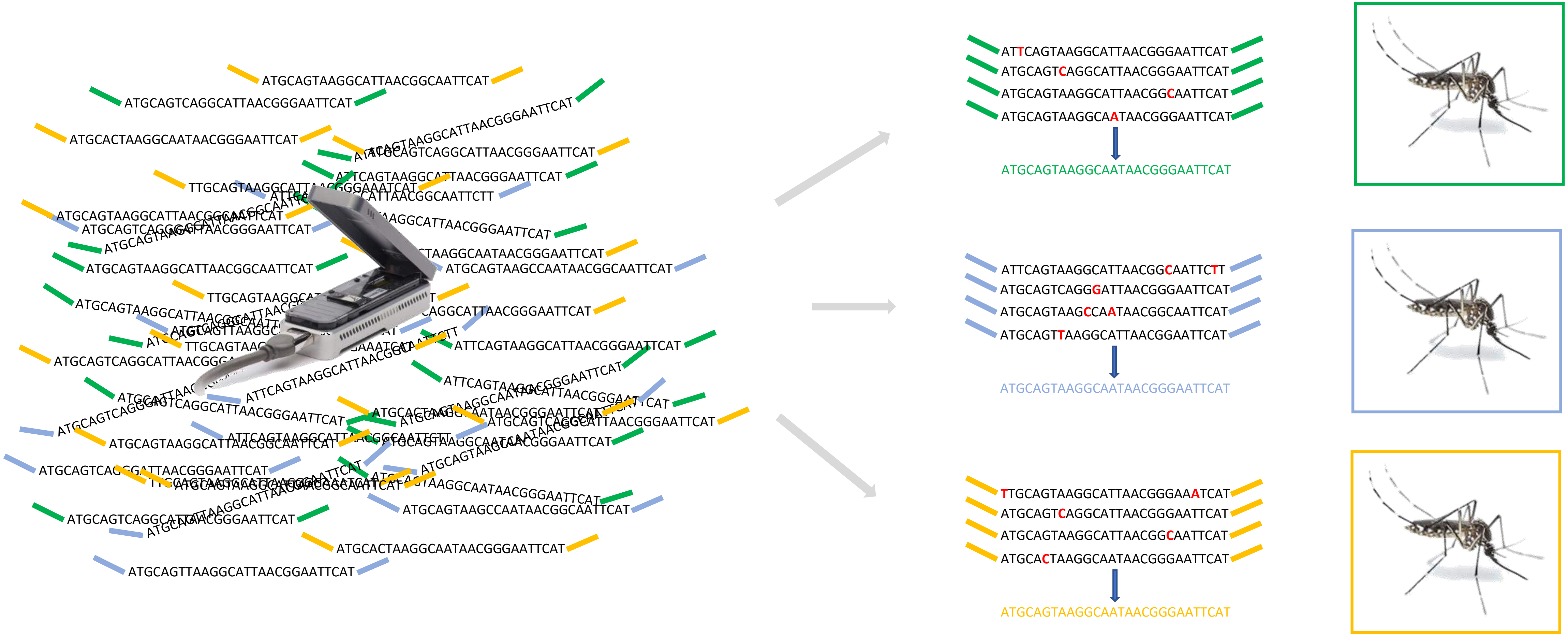

Molecular techniques of vector identification

DNA Isolation and PCR

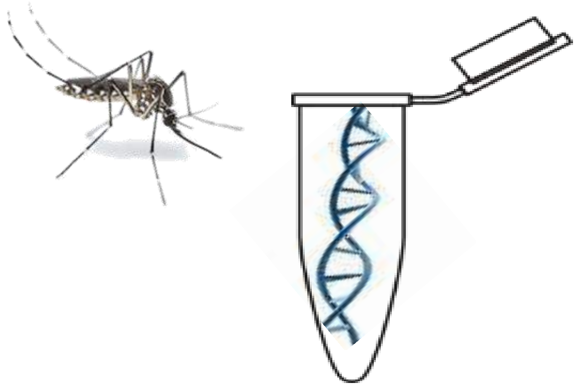

DNA Isolation – general thoughts

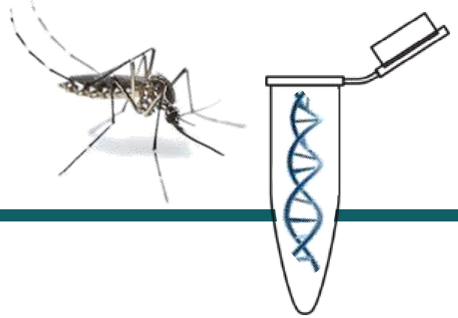

- What are our expectations for the DNA isolation?

  a) Has to work well with little input material (e.g. 1-2 mosquito legs)

  b) We want it to work under field conditions
     → Has to be easy and quick
     → Has to work without a lot of equipment

QuickExtract (Lucigen) Protocol

We only need:

- Pipette + pipette tips
- Purified water
- Forceps
- Petridish/Eppendorff tube to wash mosquito legs
- 0.2 µl tubes
- QuickExtract solution from Lucigen
- Heatblock that can reach 98 °C

DNA Isolation – Protocol

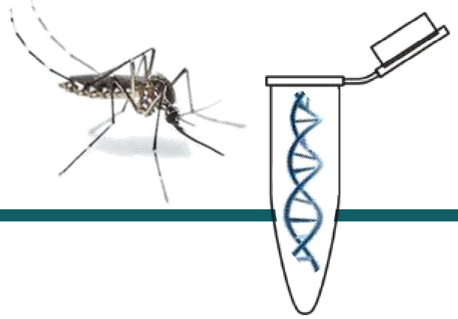

QuickExtract (Lucigen) Protocol

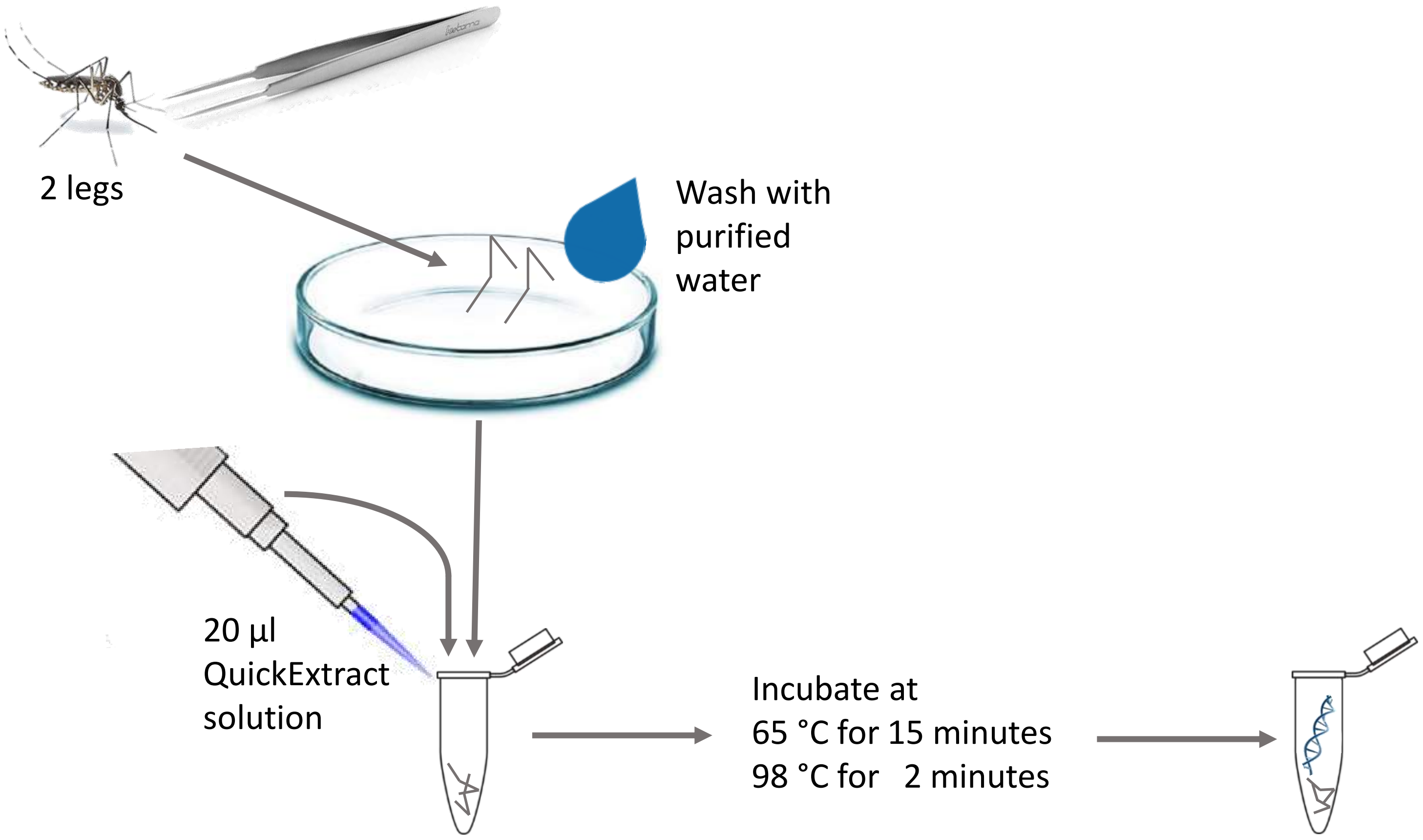

PCR – general thoughts

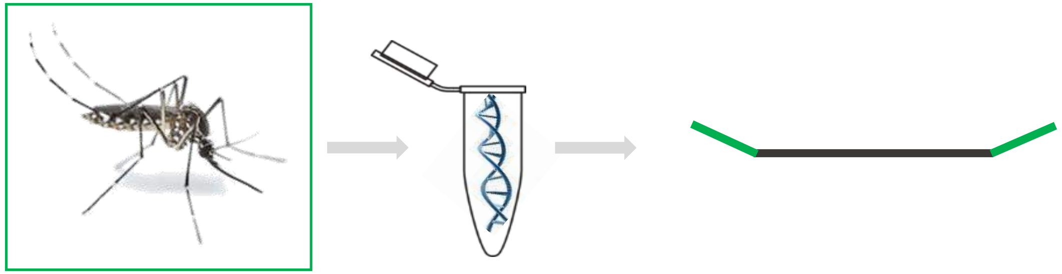

- What are our expectations for the PCR?

a) Our biggest goal is to mark individuals to allow individual-based sequencing and identification

b) Has to work reliably for different species

c) We have to choose a locus for which already enough data exists to confirm species

d) We have to choose "tags" that don't intervene with the PCR

PCR – general thoughts

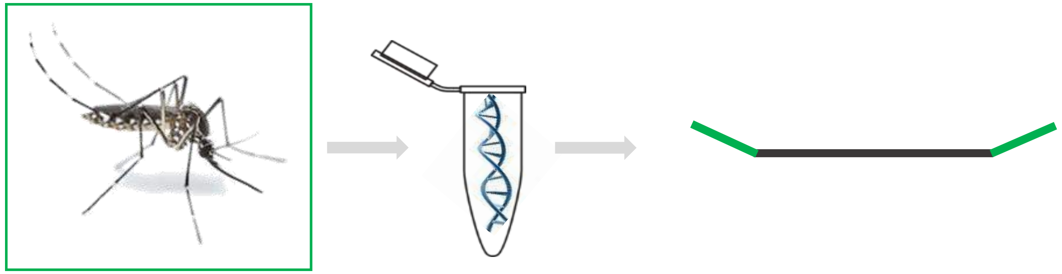

- What are our expectations for the PCR?

  a) Our biggest goal is to mark individuals to allow individual-based sequencing and identification

  → Each individual needs to be marked with a unique "code" that we can find again after sequencing

  b) Has to work reliably for different species

  c) We have to choose a locus for which already enough data exists to confirm species

  d) We have to choose "tags" that don't intervene with the PCR

PCR – tagged barcodes

Each individual needs to be marked with a unique "code" that we can find again after sequencing

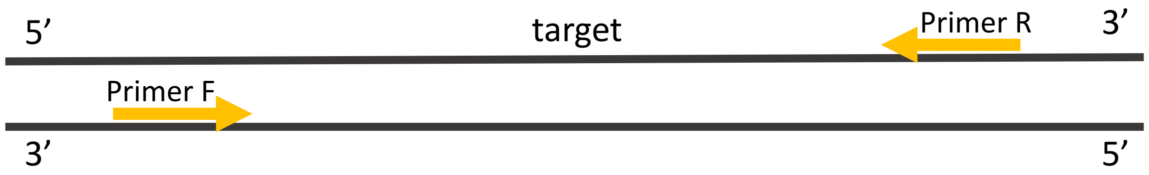

PCR – tagged barcodes

Each individual needs to be marked with a unique "code" that we can find again after sequencing

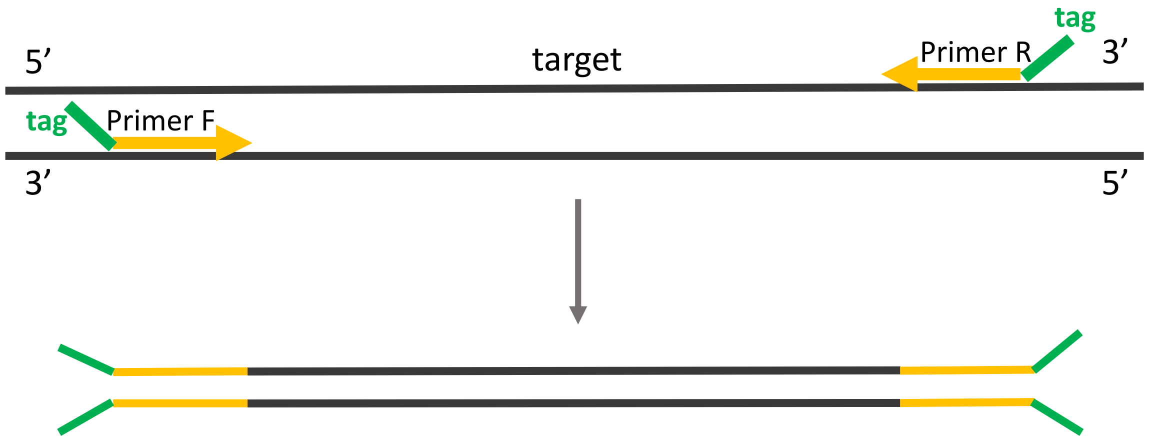

PCR – tagged barcodes

Each individual needs to be marked with a unique “code” that we can find again after sequencing

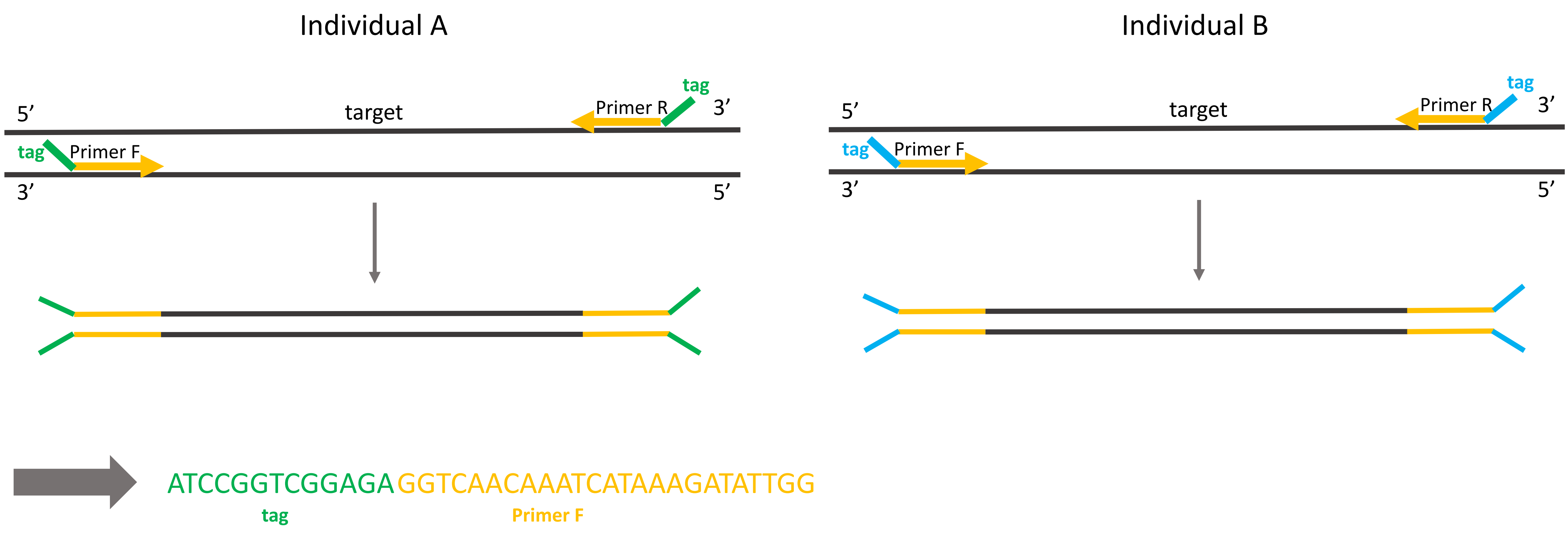

PCR – tagged barcodes: Design

ATCCGGTCGGAGA GGTCAACAAATCATAAAGATATTGG

tag | Primer

- No homopolymers >2 bp (e.g. TTT or AAA)
- Tags cannot share >6 bp sequence stretches
- Account for indels (MinION error rate!) → calculate with 3 bp errors of any kind and combination
- Cannot end in "GG"
- Length of tag is tradeoff between demultiplexing rate and PCR success

- Conserved locus, so we can use it for a variety of mosquito species
- Locus must be variable enough to distinguish closely related species
- Should already be widely used, so we can use existing databases
- Mitochondrial vs. nuclear locus

PCR – tagged barcodes: Use

ATCCGGTCGGAGA GGTCAACAAATCATAAAGATATTGG

tag

Unique or combinatorial?

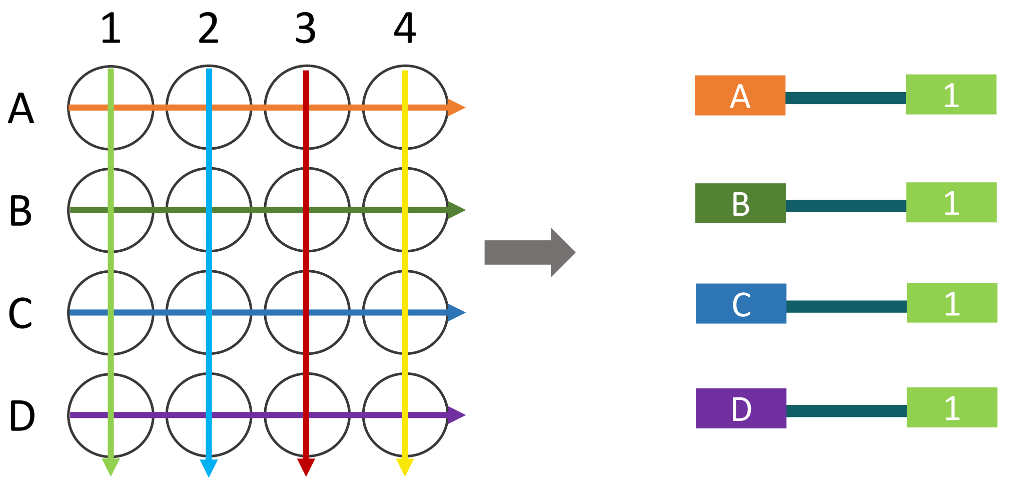

Combinatorial

Tag/index switching:

- Jumping of tag from one sequence to another
- Can happen during library preparation at the end repair step

PCR – tagged barcodes: Use

ATCCGGTCGGAGA GGTCAACAAATCATAAAGATATTGG

tag

Unique or combinatorial?

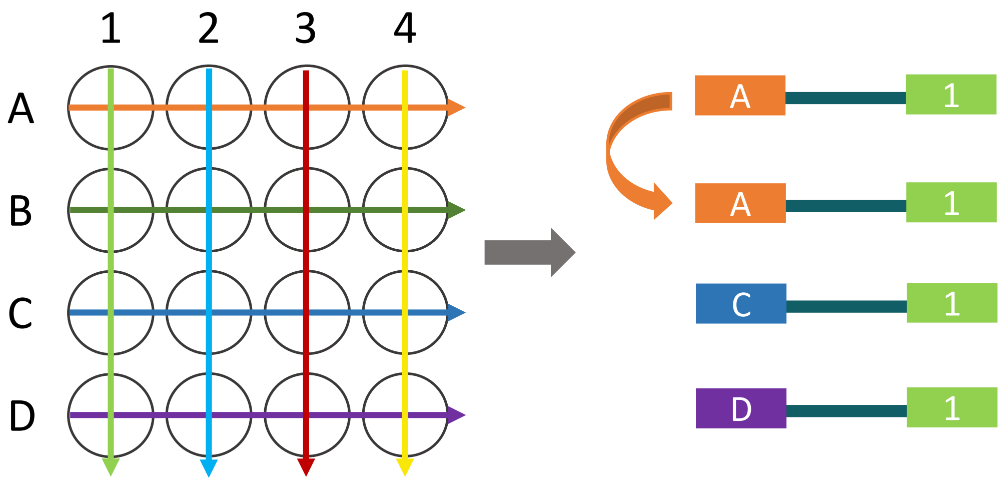

Combinatorial

Tag/index switching:

- Jumping of tag from one sequence to another
- Can happen during library preparation at the end repair step

PCR – tagged barcodes: Use

ATCCGGTCGGAGA GGTCAACAAATCATAAAGATATTGG

tag

Unique or combinatorial?

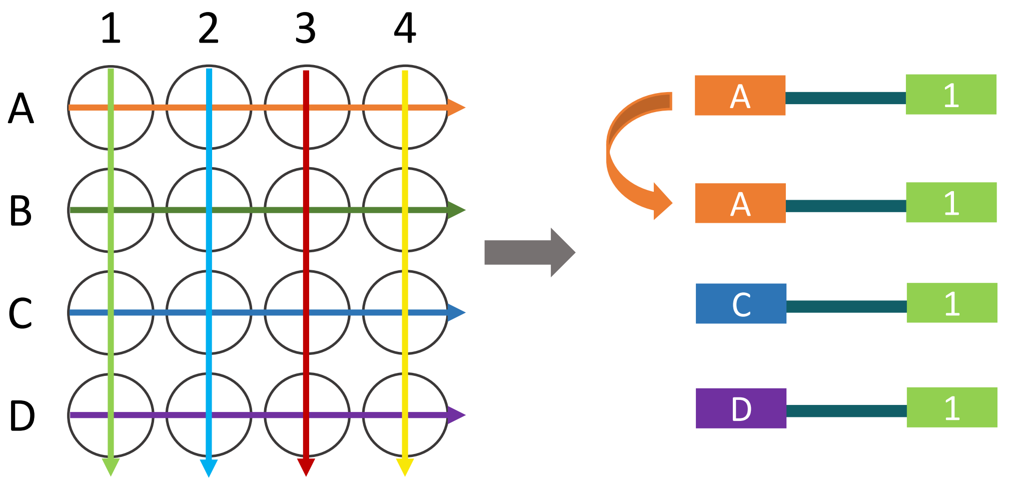

Combinatorial

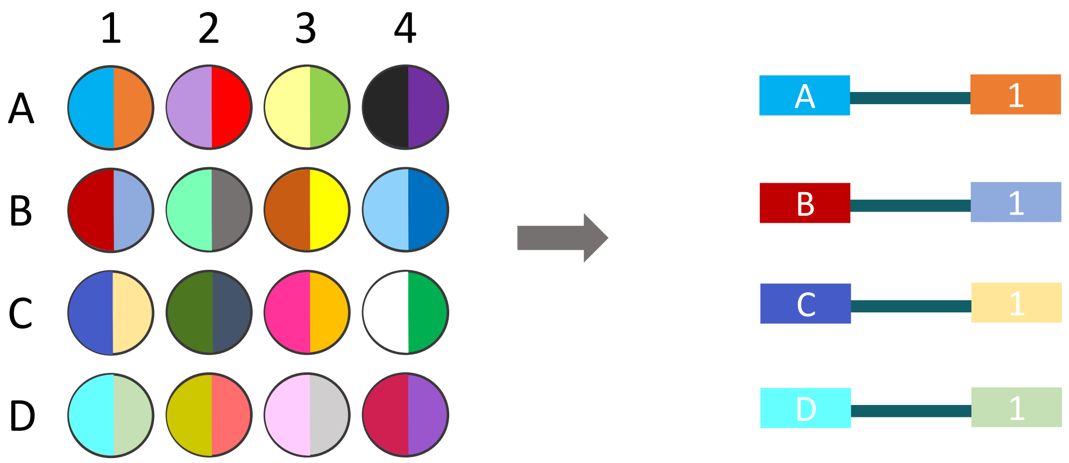

Unique

PCR – tagged barcodes: Use

ATCCGGTCGGAGA GGTCAACAAATCATAAAGATATTGG

tag

Unique or combinatorial?

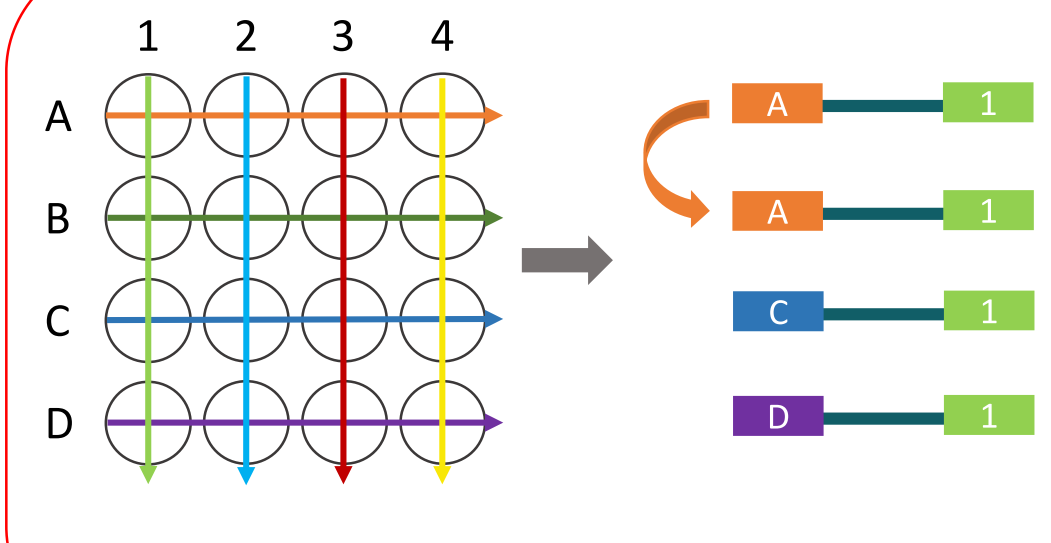

Combinatorial

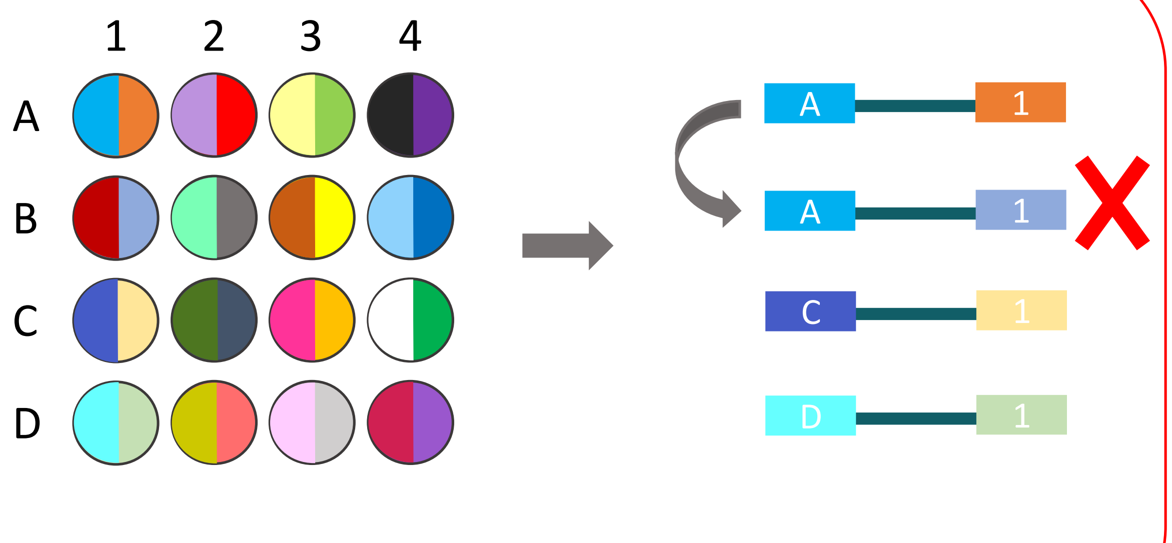

Unique

PCR – tagged barcodes: Use

ATCCGGTCGGAGA GGTCAACAAATCATAAAGATATTGG

tag

Unique or combinatorial?

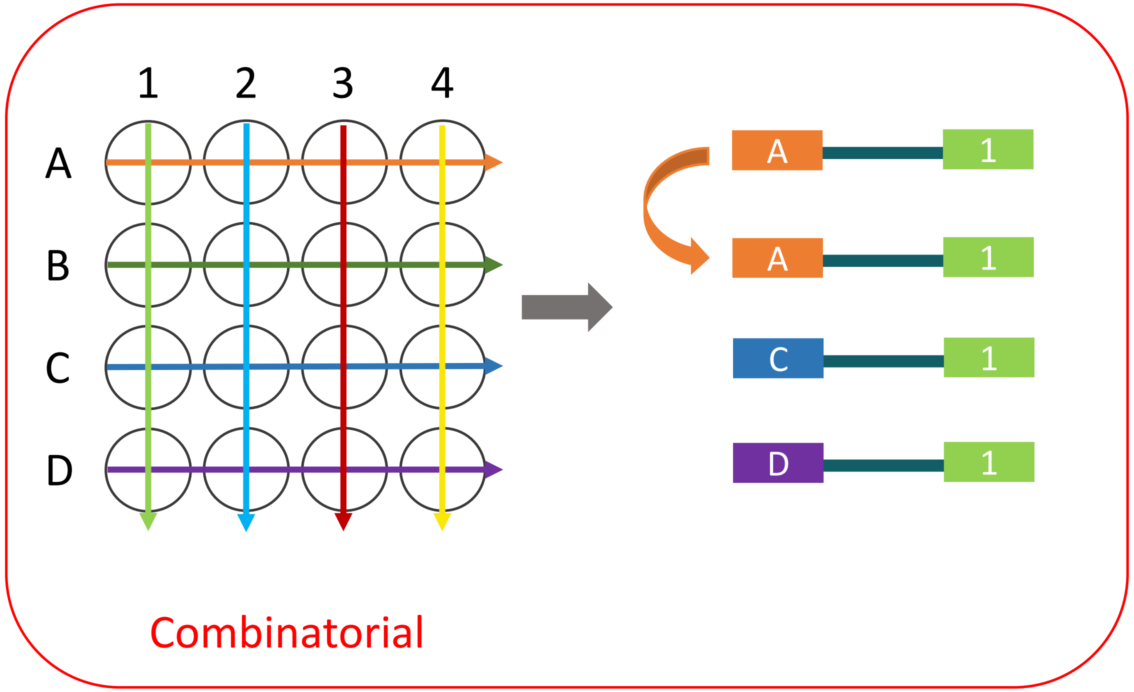

- Cheaper – not that many different primer+tags are needed
- With high enough coverage during sequencing, this approach should work well enough

PCR – Protocol

PCR Mix:

5.0 µl Mastermix
3.6 µl H2O
0.2 µl Primer F
0.2 µl Primer R
1.0 µl DNA

↓

- Since the primer combinations have to be unique for each sample, we cannot prepare a complete PCR mix and then add the DNA
- Make a plan!

Cycler settings:

94 °C	5 min	
94 °C	30 sec	35 x
45 °C	60 sec	35 x
72 °C	1 min	35 x
72 °C	5 min	

ID	Location	TagF	TagR
S1	A1	FA	R1
S2	B1	FB	R2
S3	C1	FC	R3
S4	D1	FD	R4
S5	E1	FE	R5
S6	F1	FF	R6
S7	G1	FG	R7
S8	H1	FH	R8

PCR – control

Gel electrophoresis to check the successful amplification:

- All bands visible?
- No double bands?
- No excessive primer clouds?
- Bands in the size range of ~800 bp we expect?

→

I would recommend to do this step in the beginning, when the pipeline is being established.

Afterwards, when testing many samples, this might be too much → only test random samples

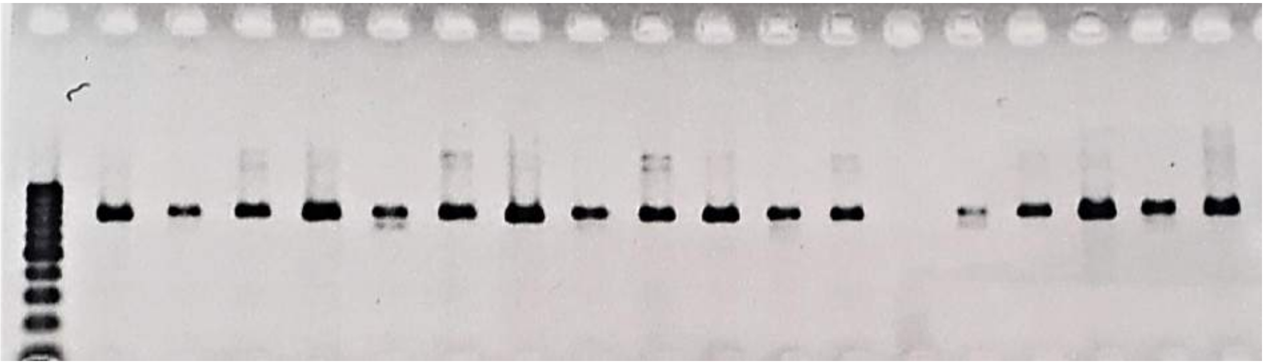

PCR

What did we do so far?

- We isolated DNA from individual mosquitoes
- We amplified the DNA (still individually)
- During amplification we marked the sequences with individual indices (tags)

Molecular techniques of vector identification

Library preparation

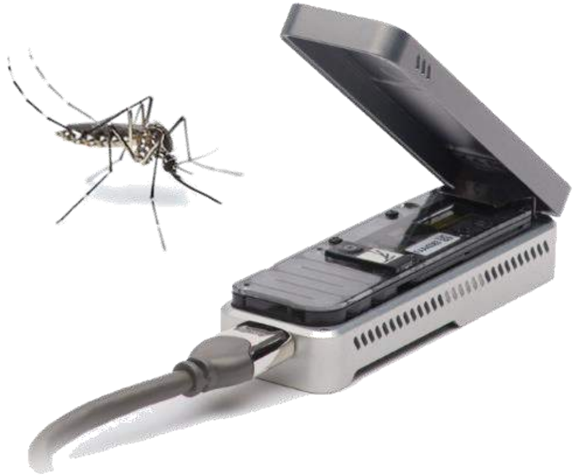

Recap

Where are we?

- We isolated DNA from individual mosquitoes
- We amplified the DNA (still individually)
- During amplification we marked the sequences with individual indices (tags)

What's next?

- We want to prepare the PCR products for sequencing (library preparation)
- We want to sequence all our PCR products together

Library preparation : What is a “library”?

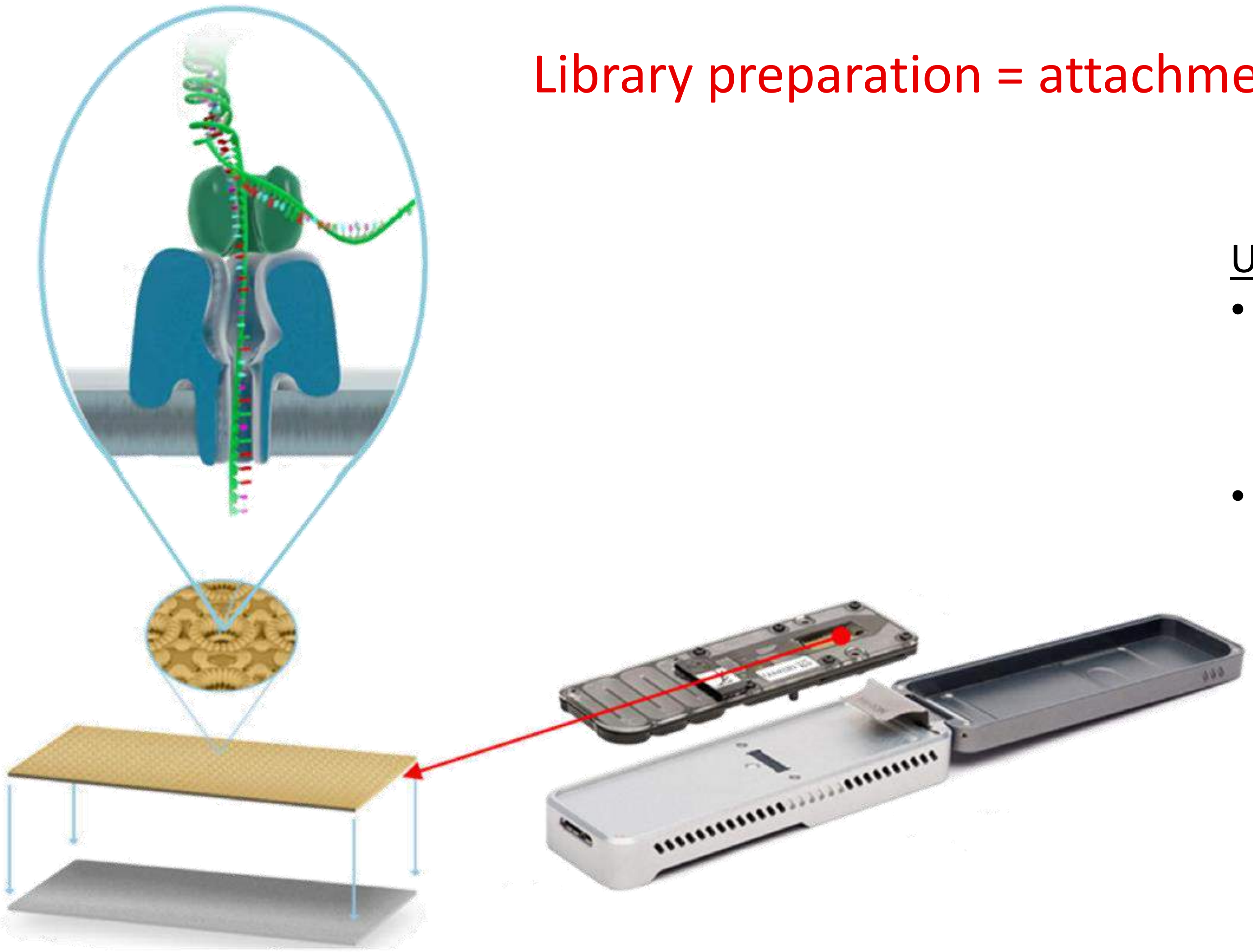

Library preparation = attachment of sequencing adapters to our DNA

Use of sequencing adapters:

- Motor protein
  - → Slows down speed of the DNA in the nanopore
  - → Unwinds the double strand
- Tether
  - → Improves sensitivity

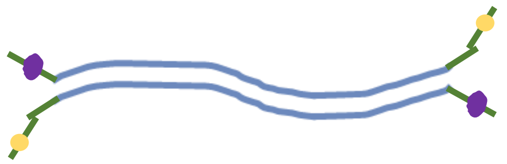

Library preparation : To Do

Pooling PCR products

DNA repair

End repair

dA tailing

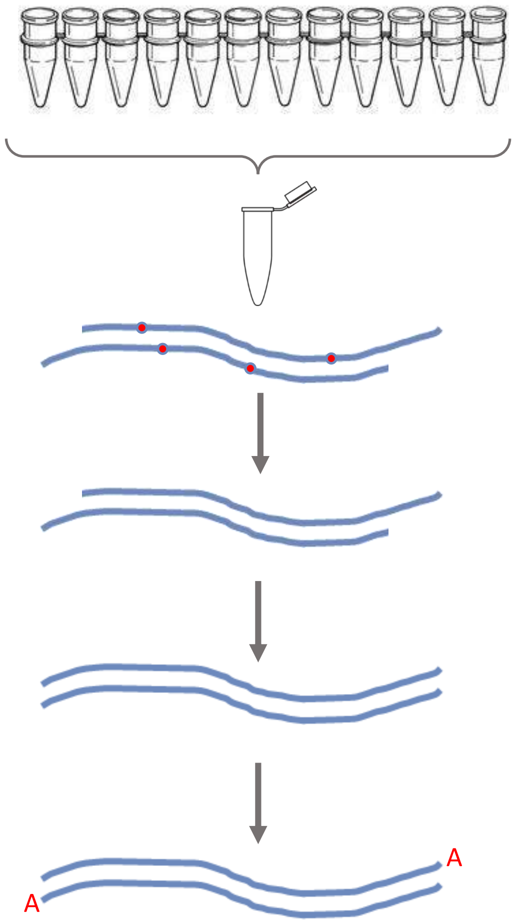

Magnetic bead Clean-up

Adapter ligation

Magnetic bead Clean-up

Prepare Flow cell

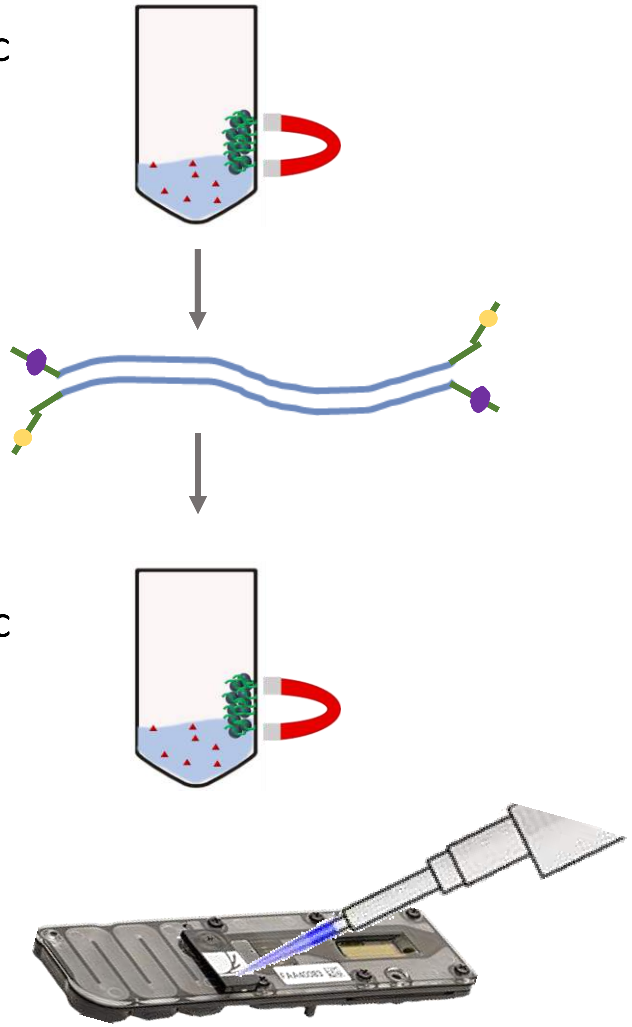

Library preparation

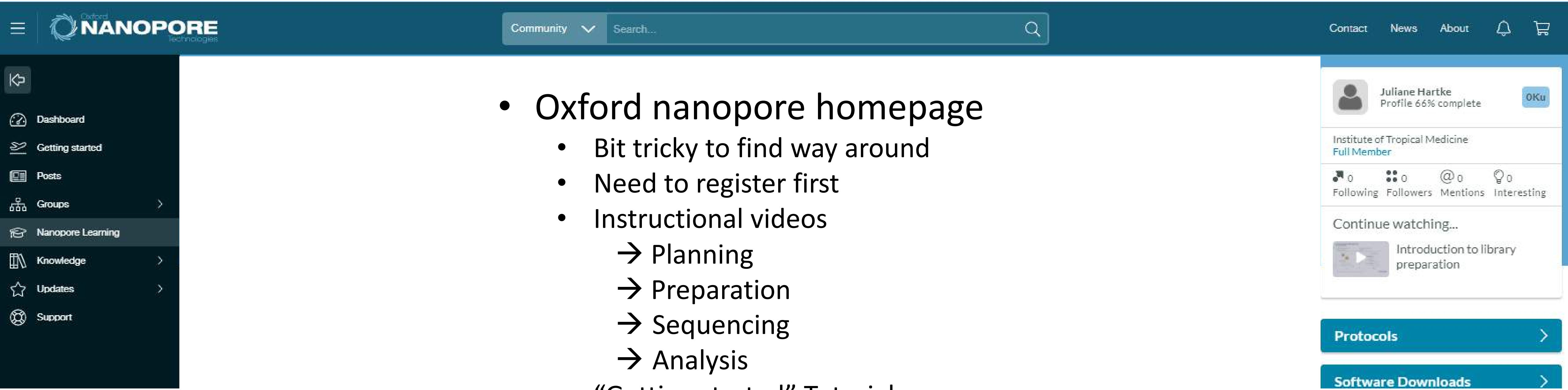

- Oxford nanopore homepage
  - Bit tricky to find way around
  - Need to register first
  - Instructional videos
    - → Planning
    - → Preparation
    - → Sequencing
    - → Analysis
  - “Getting started” Tutorials

https://community.nanoporetech.com/nanopore_learning/lessons

I highly recommend watching the relevant videos shortly before you start sequencing with the MinION!

Library preparation: What do we need?

- MinION sequencer
- MinION flow cell
- Ligation sequencing kit (SQK-LSK109)
- Computer that fulfills MinION standards
- AMPure XP beads
- NEBNext FFPE Repair Mix
- NEBNext Ultra II End repair/dA-tailing Module
- NEBNext Quick Ligation Module
- 1.5 ml Eppendorf LoBind tubes
- 0.2 ml PCR tubes
- Nuclease free water
- 70% Ethanol (in nuclease free water)
- Magnetic rack
- Microfuge
- Vortex mixer
- Thermal cycler
- Ice
- Pipettes and tips (P2, P10, P20, P100, P200, P1000)

The specifications for these change often, and library preparation packages can be discontinued!
Especially check the required Computer specifications!

These additional reagents are based on the Ligation Sequencing kit (SQK-LSK109). Depending on which kit is used they may change.

Library preparation: What do we need?

- MinION sequencer
- MinION flow cell
- Ligation sequencing kit (SQK-LSK109)
- Computer that fulfills MinION standards
- AMPure XP beads
- NEBNext FFPE Repair Mix
- NEBNext Ultra II End repair/dA-tailing Module
- NEBNext Quick Ligation Module
- 1.5 ml Eppendorf LoBind tubes
- 0.2 ml PCR tubes
- Nuclease free water
- 70% Ethanol (in nuclease free water)
- Magnetic rack
- Microfuge
- Vortex mixer
- Thermal cycler
- Ice
- Pipettes and tips (P2, P10, P20, P100, P200, P1000)

We want to be able to apply the protocol under field conditions.
How does that work?

Library preparation: What do we need?

- Microfuge
- Vortex mixer
- Thermal cycler
- Ice

Gel-electrophoresis

Microfuge

Thermal cycler

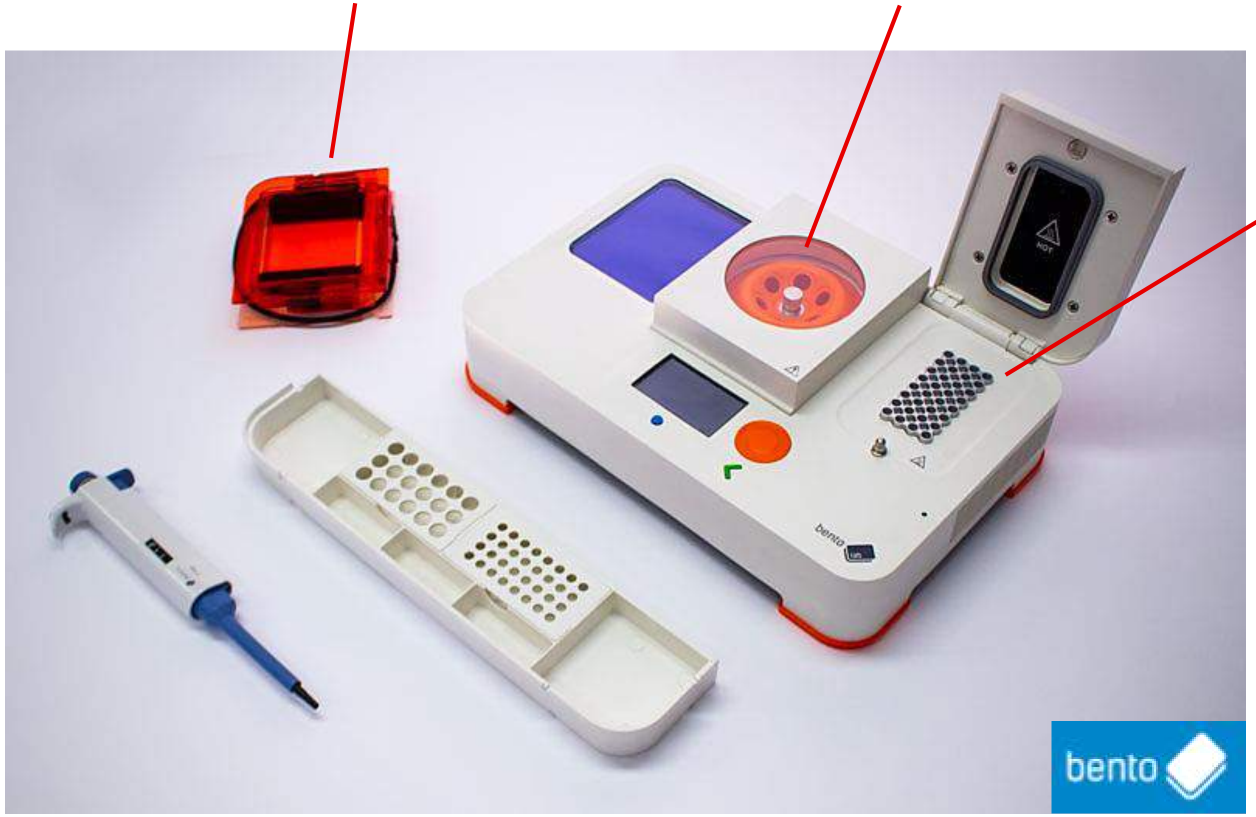

Can also be used for the PCR step

Library preparation: What do we need?

- Microfuge
- Vortex mixer → Use the “finger flick” or/and mix by pipetting or shaking the tube
- Thermal cycler
- Ice

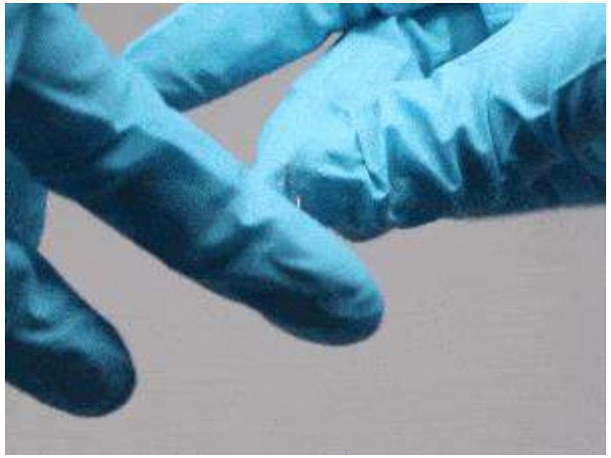

Library preparation: How to start?

- Read the nanopore protocol carefully beforehand
- Try to estimate how much time you will need (in the beginning: take double the amount that nanopore estimates)
- Check if everything that you need is there

  → Every protocol contains a checklist that you can use!

Equipment and consumables

Materials

- 1 µg (or 100-200 fmol) high molecular weight genomic DNA
- 1.5-3 µg (or 150-300 fmol) high molecular weight genomic DNA if using R10.3 flow cells
- OR 100+ ng high molecular weight genomic DNA if performing DNA fragmentation
- Ligation Sequencing Kit (SQK-LSK109)
- Flow Cell Priming Kit (EXP-FLP002)

Consumables

- Agencourt AMPure XP beads
- NEBNext® Companion Module for Oxford Nanopore Technologies® Ligation Sequencing (cat # E7180S). Alternatively, you can use the three NEBNext® products below:
- NEBNext FFPE Repair Mix (M6630)
- NEBNext Ultra II End repair/dA-tailing Module (E7546)
- NEBNext Quick Ligation Module (E6056)
- 1.5 ml Eppendorf DNA LoBind tubes
- 0.2 ml thin-walled PCR tubes
- Nuclease-free water (e.g. ThermoFisher, cat # AM9937)

Library preparation: How to start?

Long protocol version

1 **Thaw DNA CS (DCS) at room temperature, spin down, mix by pipetting, and place on ice.**

2 **Prepare the NEBNext FFPE DNA Repair Mix and NEBNext Ultra II End repair / dA-tailing Module reagents in accordance with manufacturer's instructions, and place on ice.**

3 **Prepare the DNA in nuclease-free water**

- For R9.4.1 flow cells, transfer 1 µg (or 100-200 fmol) genomic DNA into a 1.5 ml Eppendorf DNA LoBind tube, or 1.5-3 µg (or 150-300 fmol) genomic DNA if using R10.3 flow cells.
- Adjust the volume to 49 µl with nuclease-free water
- Mix thoroughly by flicking the tube
- Spin down briefly in a microfuge

4 **In a 0.2 ml thin-walled PCR tube, mix the following:**

Reagent	Volume
DNA CS	1 µl
DNA	47 µl
NEBNext FFPE DNA Repair Buffer	3.5 µl
NEBNext FFPE DNA Repair Mix	2 µl
Ultra II End-prep reaction buffer	3.5 µl
Ultra II End-prep enzyme mix	3 µl
**Total**	**60 µl**

5 **Mix gently by flicking the tube, and spin down.**

6 **Using a thermal cycler, incubate at 20°C for 5 mins and 65°C for 5 mins.**

Check-list version

INSTRUCTIONS	NOTES/OBSERVATIONS
In a 0.2 ml thin-walled PCR tube, mix the following:<br>☐ 1 µl DNA CS<br>☐ 47 µl DNA<br>☐ 3.5 µl NEBNext FFPE DNA Repair Buffer<br>☐ 2 µl NEBNext FFPE DNA Repair Mix<br>☐ 3.5 µl Ultra II End-prep reaction buffer<br>☐ 3 µl Ultra II End-prep enzyme mix<br>☐ Mix gently by flicking the tube, and spin down.<br>☐ Using a thermal cycler, incubate at 20°C for 5 mins and 65°C for 5 mins.	

- Long version is useful in the beginning because it is more detailed
- Check-list version is useful to tick off all steps and note observations
- Also check out online version with additional explanations and Videos

Library preparation: How to start?

- Download the MinION software for running the sequencing protocol
- Connect the MinION device to your computer and perform hardware check
- Clean work surfaces with ethanol
- Thaw the reagents needed for library preparation and put them on ice
- Quantify the DNA in the pooled PCR products (e.g. with Qubit, Nanodrop..)
  - ➢ Recommended amount depends on type of flow cell! (Check protocol)

Library preparation: How to start?

LNB	LNB: Ligation Buffer	Thaw at room temperature, spin down, place on ice
SFB	SFB: Short Fragment Buffer	Thaw at room temperature, mix by vortexing, spin down, place on ice
EB	EB: Elution Buffer	Thaw at room temperature, mix by vortexing, spin down, place on ice
DCS	DCS: DNA control strand	Thaw at room temperature, mix by pipetting, spin down, place on ice
FRB	FRB: NEB FFPE DNA Repair Buffer	Prepare according to manufacturers instructions, place on ice
FRM	FRM: NEB FFPE DNA Repair Mix	
URB	URB: Ultra End-prep Reaction Buffer	
UEM	UEM: Ultra End-prep Enzyme Mix	
T4	T4: NEBNext Quick T4 DNA Ligase	Spin down, place on ice
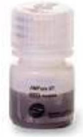	AMPure XP beads	Place at room temperature

Library preparation

① Pooling of PCR products

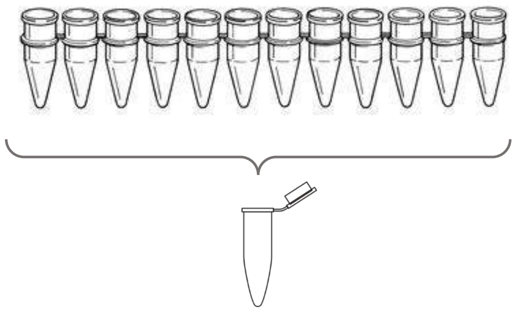

Pool in 0.2 ml thin-walled PCR tube

- Result: DNA in nuclease free water (end volume: 49 µl)
- Quantify pooled product and refer to protocol about desired amount
  → Dilute if necessary

Library preparation

② DNA repair and end-prep

DNA CS	1 µl	DCS
NEBNext FFPE DNA Repair Buffer	3.5 µl	FRB
NEBNext FFPE DNA Repair Mix	2 µl	FRM
Ultra II End-prep reaction buffer	3.5 µl	URB
Ultra II End-prep reaction buffer	3 µl	UEM

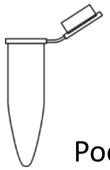

Pooled DNA

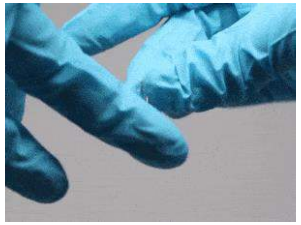

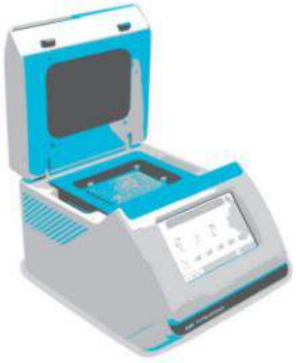

5 min at 20° C
5 min at 65° C

Library preparation

3 Magnetic bead clean-up

1:1 magnetic beads (60 µl)

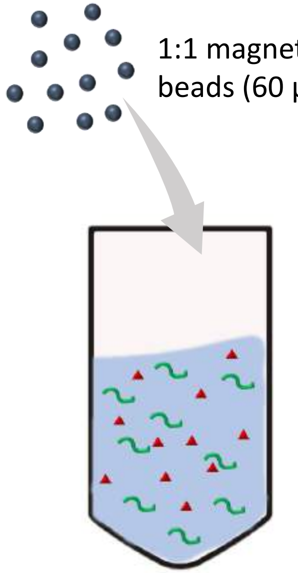

DNA with unwanted material

- Make sure beads are properly mixed
- Add 60 µl to DNA mix
- Mix tube by flicking

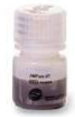

<u>Magnetic bead size selection:</u>

<1:1 bead:DNA → for longer DNA fragments

>1:1 bead:DNA → for shorter DNA fragments

We use 1:1 ratio

- Will select against <100 bp fragments
- Gets rid of primer dimers, free adapters

Library preparation

③ Magnetic bead clean-up

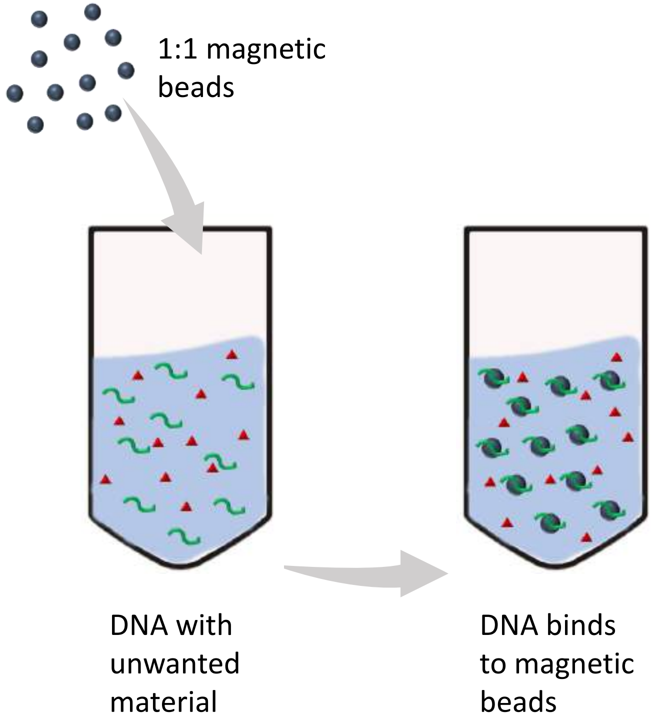

- Incubate for 5 min at room temperature

Protocol advises for Hula mixer

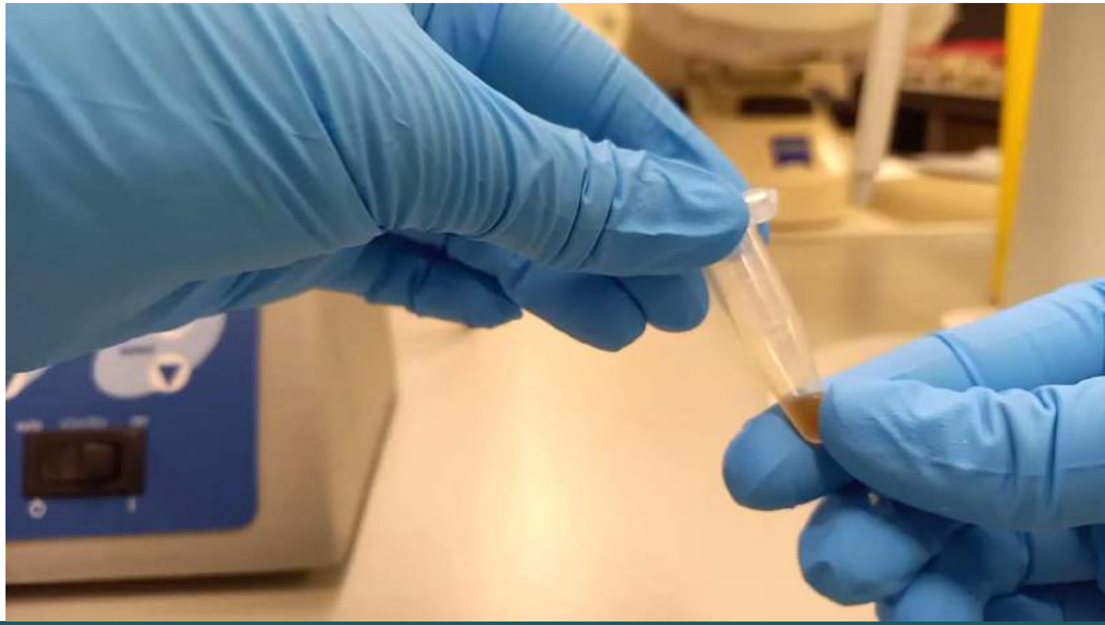

Library preparation

③ Magnetic bead clean-up

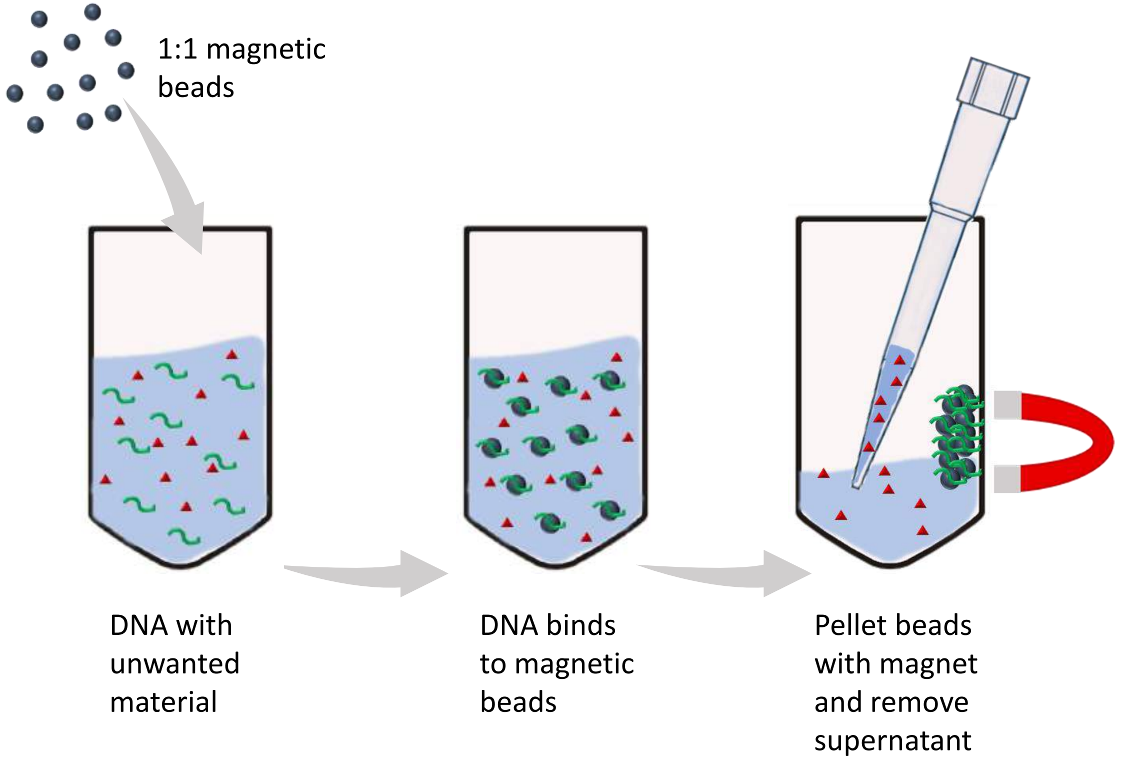

- Formation of pellet can take a while
- If the pellet is rather large and spread out, try playing around with the position of the magnet
- Only continue when the supernatant is clear

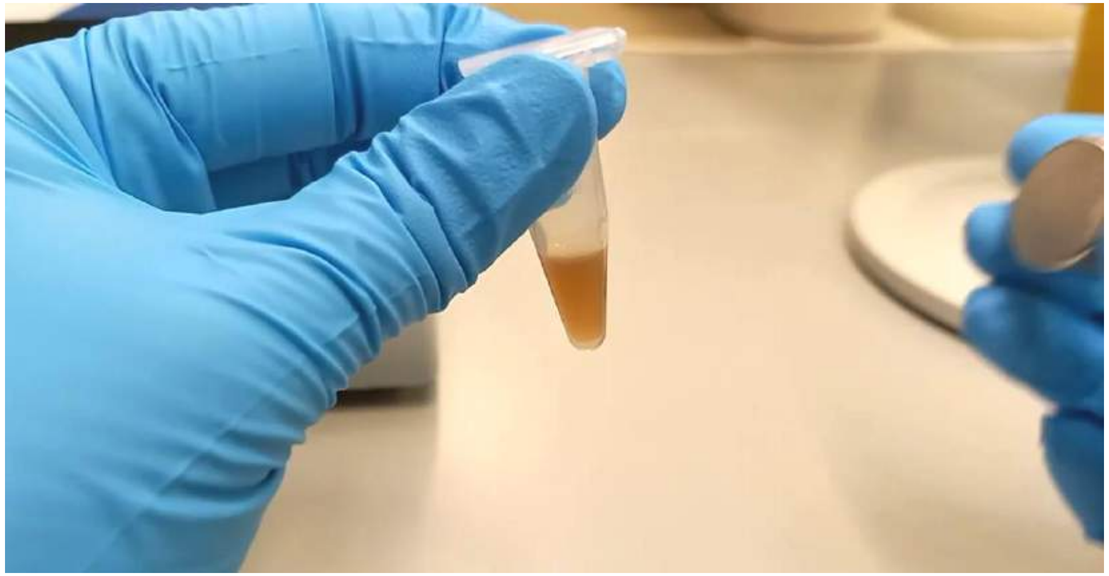

Library preparation

③ Magnetic bead clean-up

- Pipette tip at opposite site of pallet
- Remove supernatant slowly to not disturb the pellet

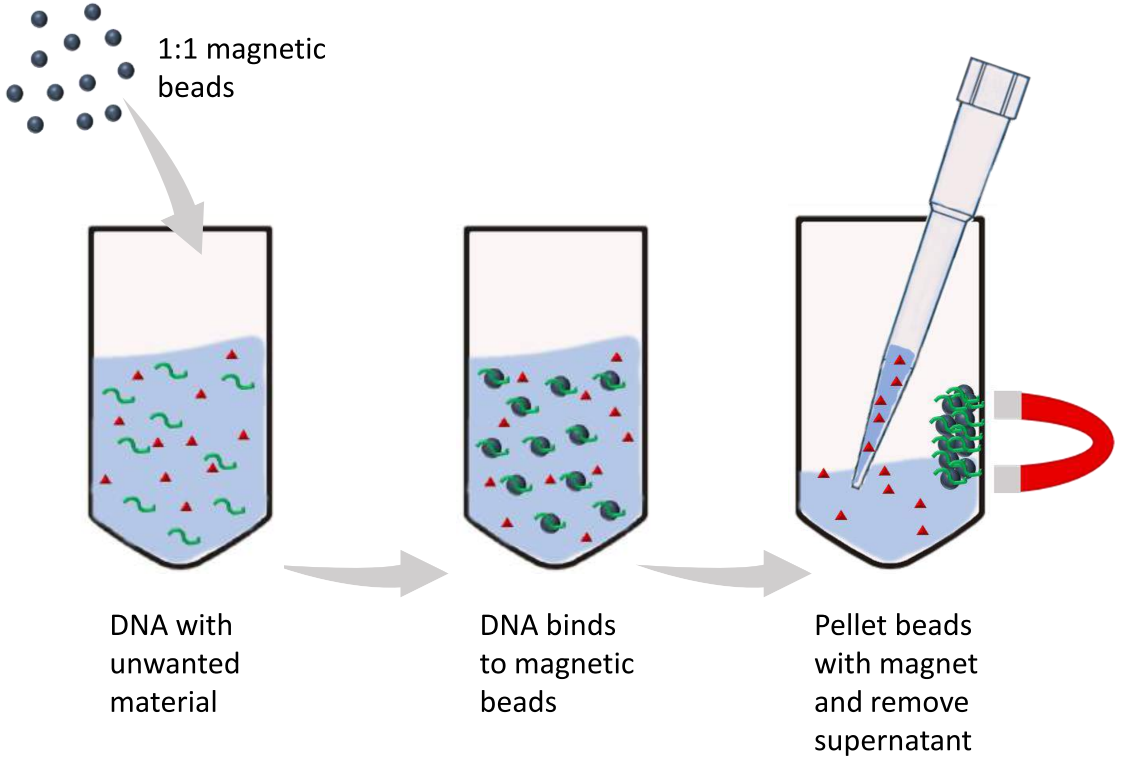

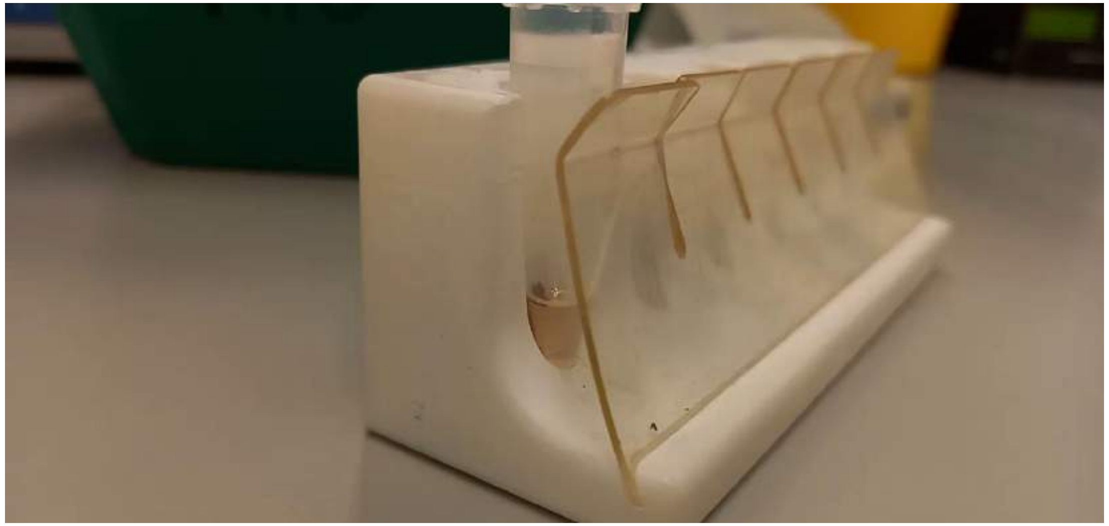

Library preparation

③ Magnetic bead clean-up

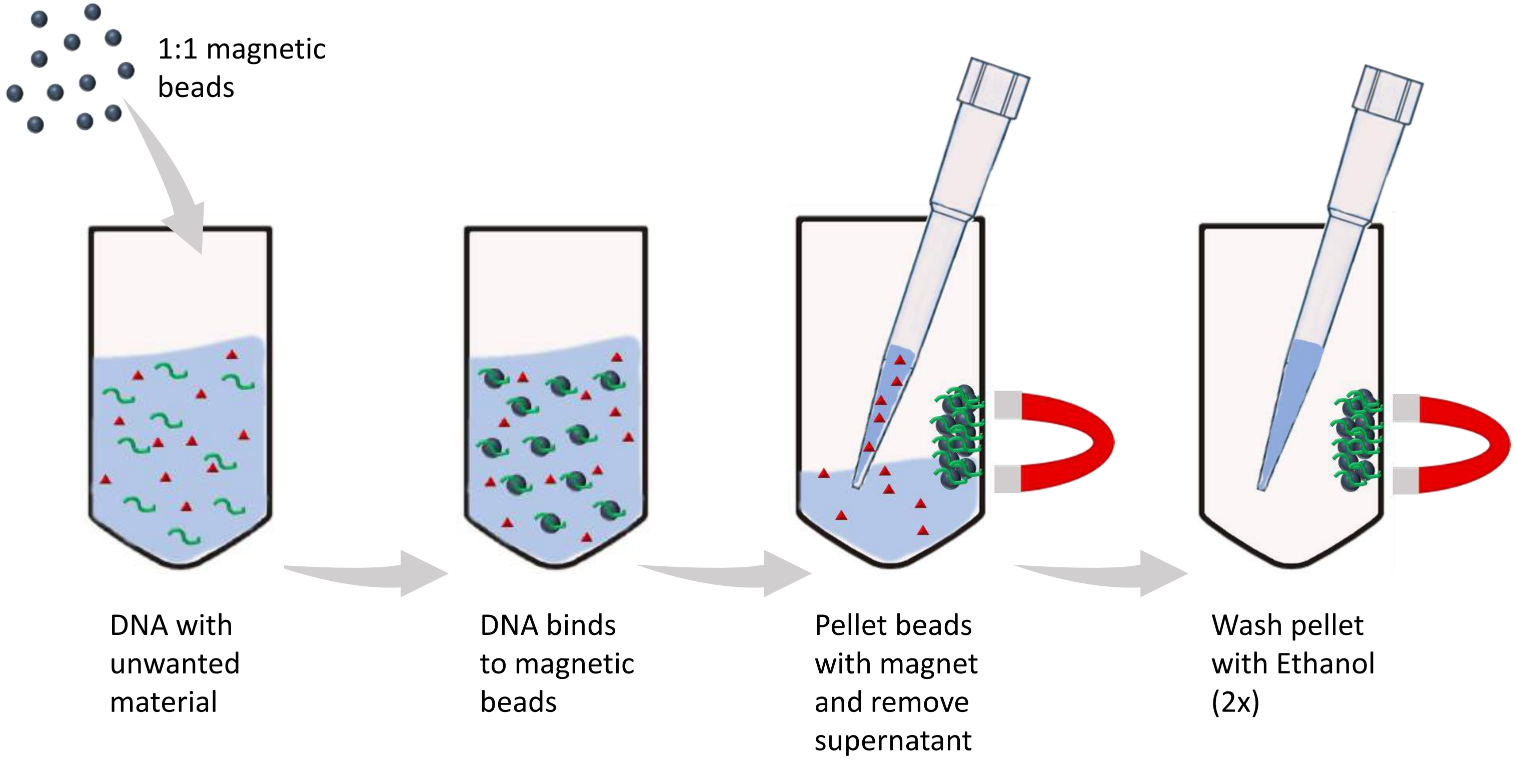

- Add 200 µl 70% ethanol slowly to not disturb the pellet, use opposite side
- After second wash step: spin down, re-pellet on magnet and pipette off remaining ethanol
- Allow to dry for 30 sec

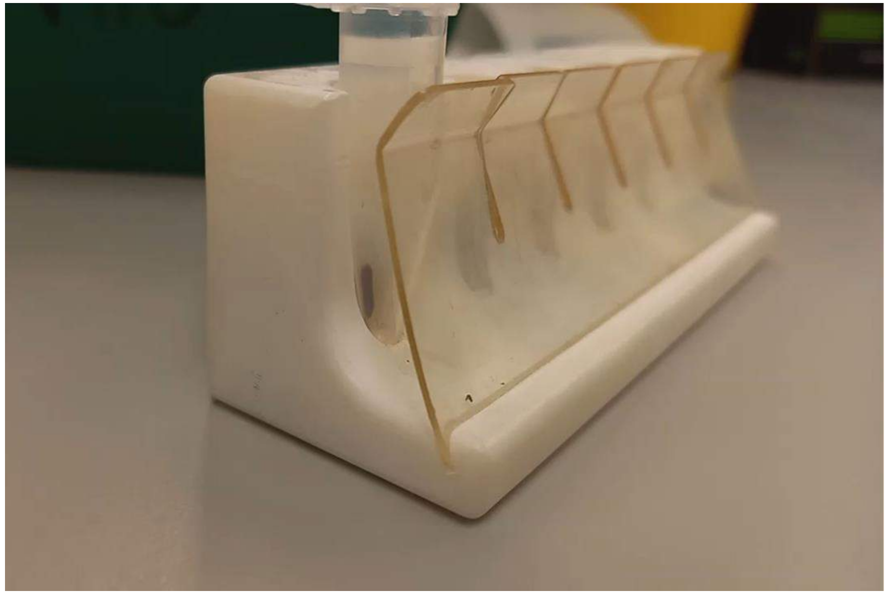

Library preparation

③ Magnetic bead clean-up

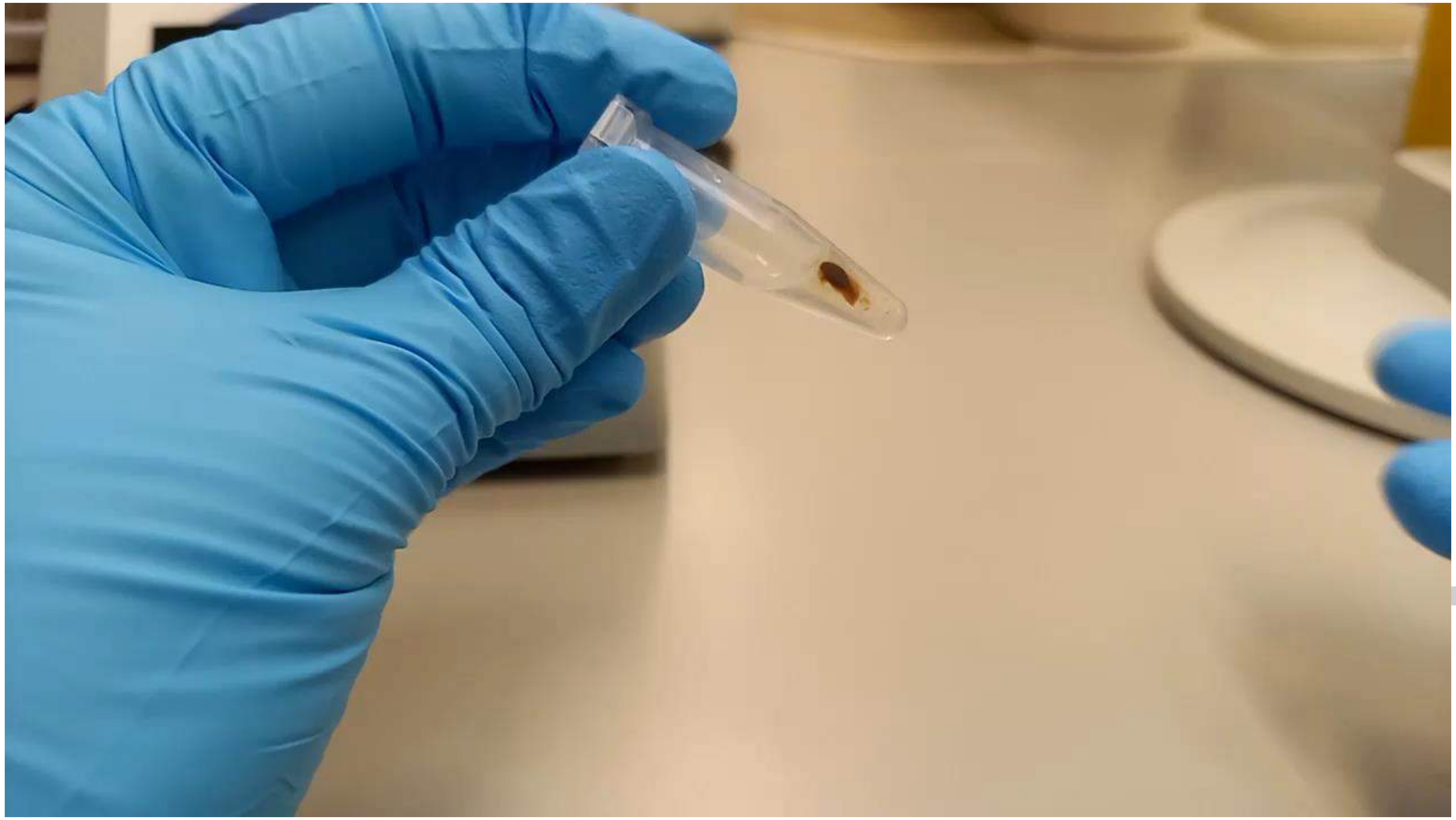

- Resuspend in 61 µl nuclease free water
- Make sure pellet is completely resuspended
- Incubate for 2 min at RT

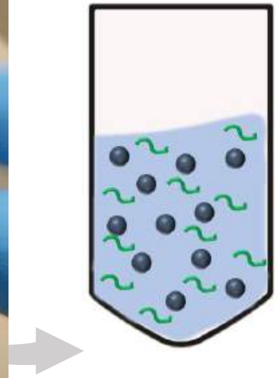

Resuspend pellet in nuclease free water

Library preparation

3 Magnetic bead clean-up

- Return tube to magnet and pellet beads
- Carefully transfer supernatant to new tube

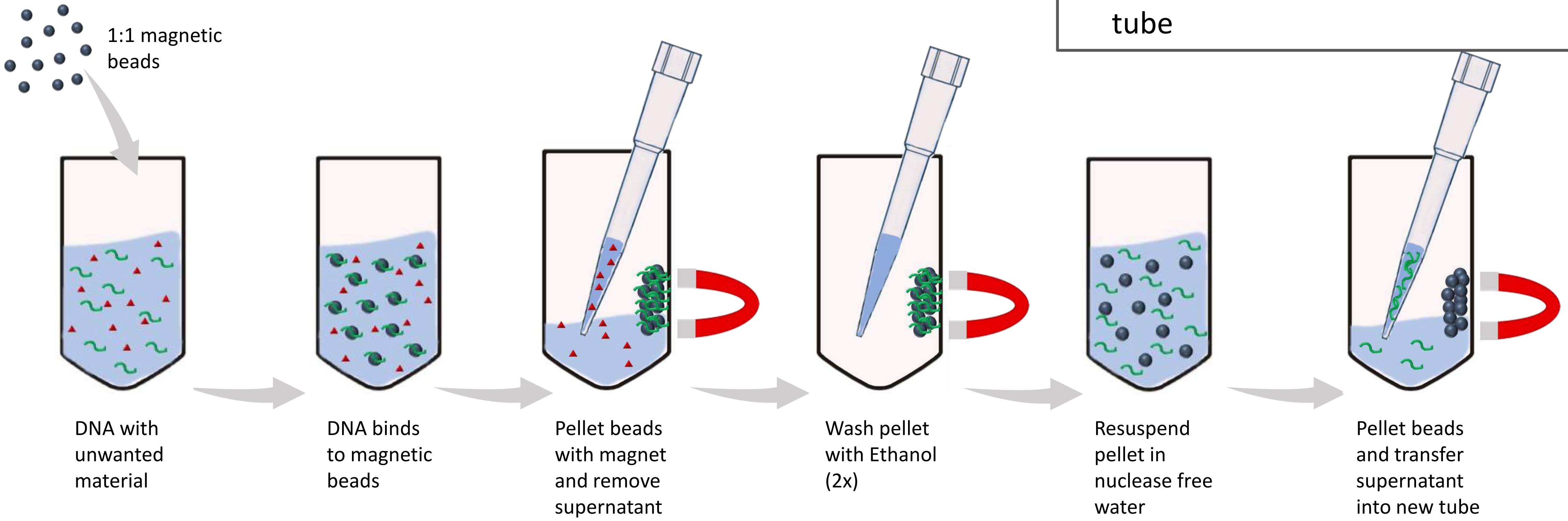

Library preparation

4 Adapter ligation

- Add all reagents to DNA from previous step

Ligation Buffer (LNB)	25 µl	LNB
NEBNext Quick T4 DNA Ligase	10 µl	T4
Adapter Mix (AMX)	5 µl	AMX

Mix by pipetting (vortexing does not work due to high viscosity)

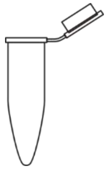

Add to DNA

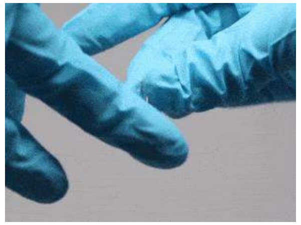

Mix by flicking the tube

Incubate for 10 min at room temperature

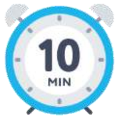

Library preparation

5 Magnetic bead clean-up

- Protocol says to use 0.4:1 ratio of beads, but we again use 1:1
- Same protocol as last time, this time we wash with Short Fragment buffer and resuspend DNA in Elution buffer

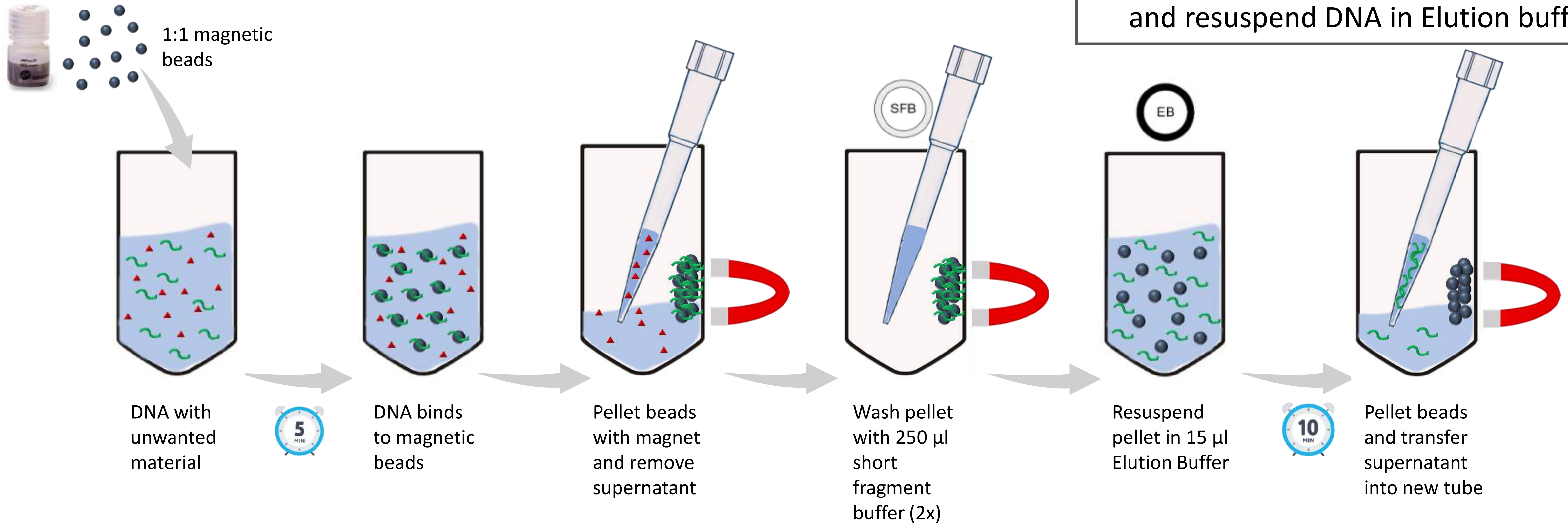

Library preparation: Recap

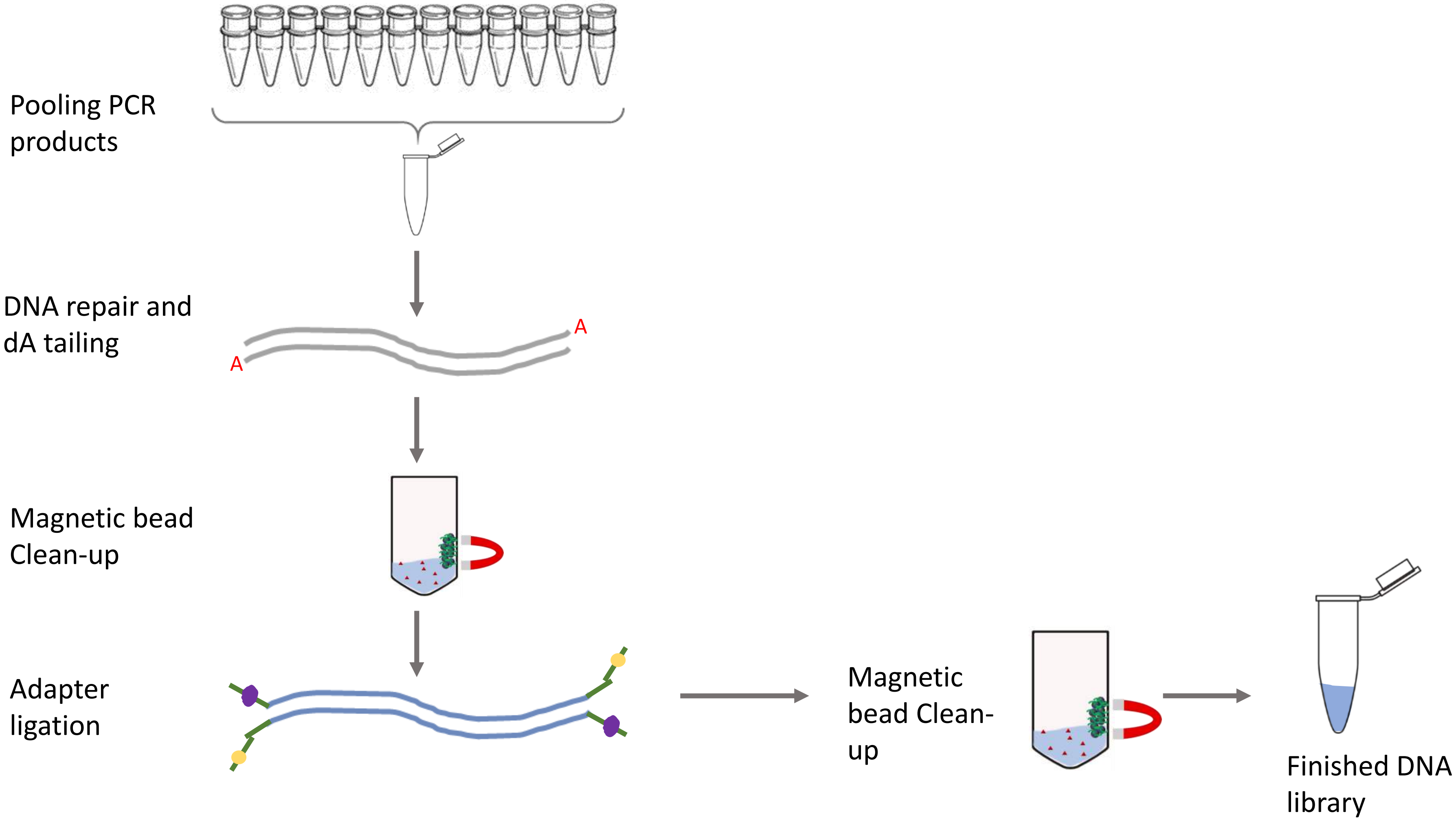

Prepare Flow cell

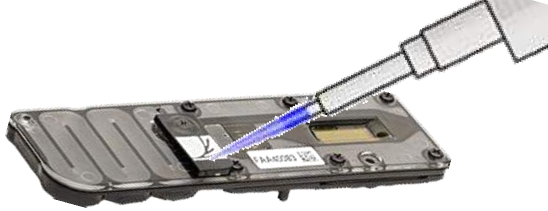

- SQB: Sequencing Buffer → Thaw at room temperature, mix by vortexing, spin down
- LB: Loading beads → Thaw at room temperature
- FB: Flush Buffer → Thaw at room temperature, mix by vortexing, spin down
- FLT: Flush Tether → Thaw at room temperature, mix by vortexing, spin down

- Open the MinION lid and place the flow cell under the clip
- Open the priming port

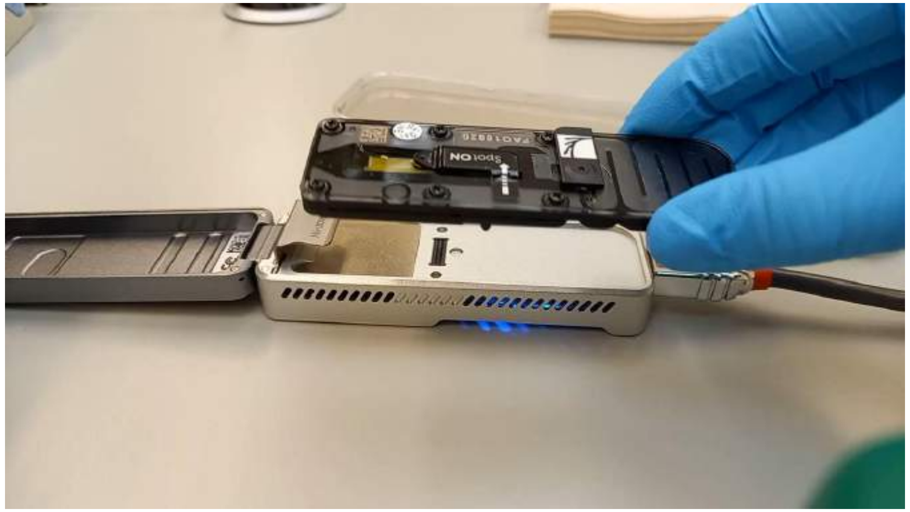

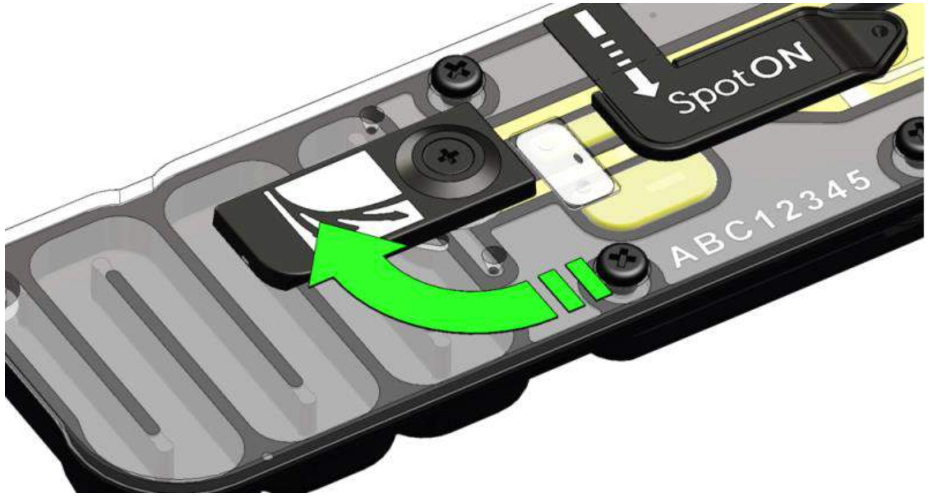

Prepare Flow cell

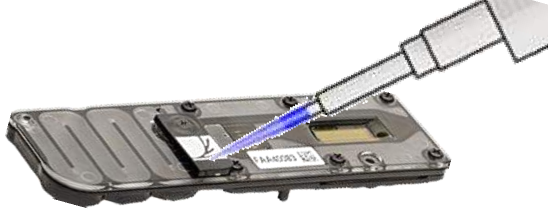

- Remove any potential air bubbles
- P1000 pipette: set to 200 µl
- Insert tip into priming port
- Adjust volume dial slowly until a small volume of buffer enters the pipette tip

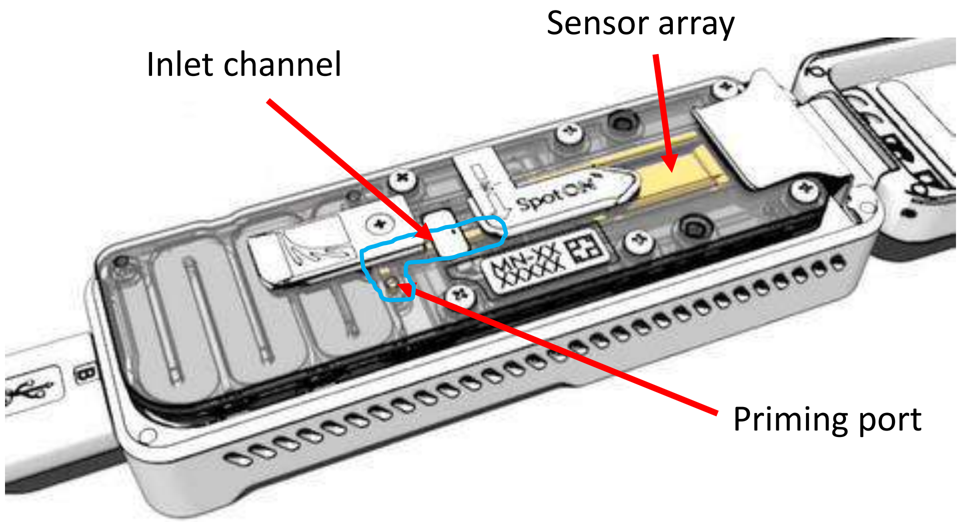

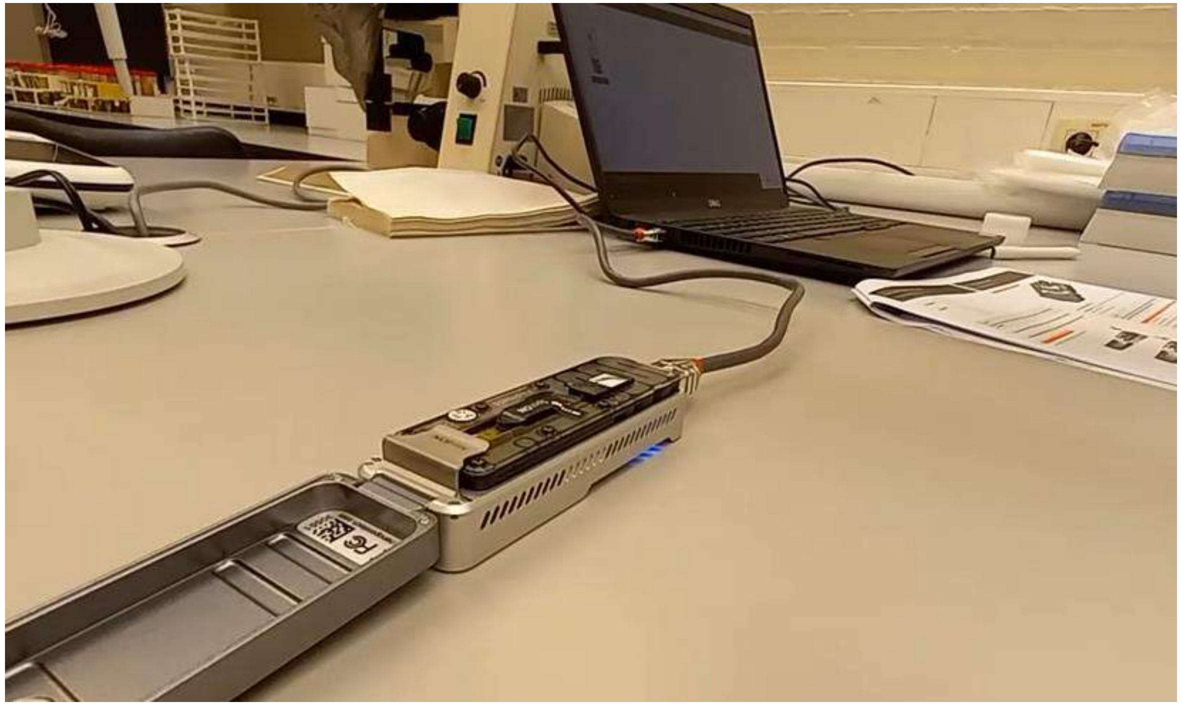

Prepare Flow cell

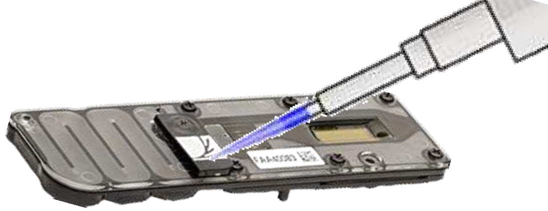

FLT: Flush Tether

30 µl FLT into FB tube

FB: Flush Buffer

Mix by vortexing

800 µl into priming port

5 MIN

Open SpotON port

200 µl into priming port

- Insert tip into priming port
- Pipette slowly
- Leave a tiny bit of buffer in the pipette – this prevents introduction of new air bubbles
- Before 2nd load: open SpotON port

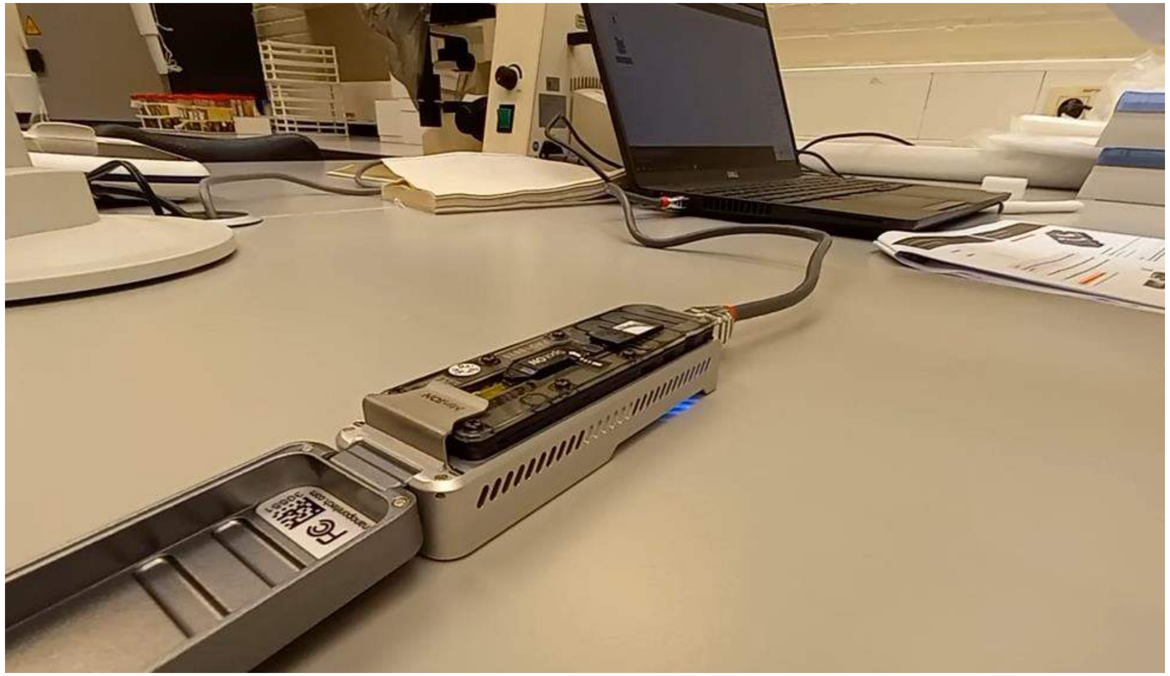

Prepare Flow cell

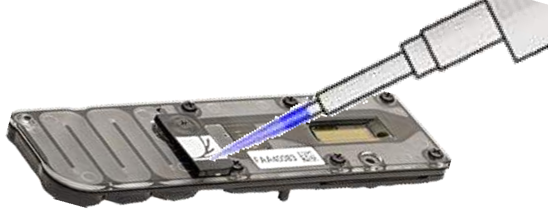

FLT: Flush Tether

30 µl FLT into FB tube

FB: Flush Buffer

Mix by vortexing

800 µl into priming port

Open SpotON port

200 µl into priming port

- Insert tip into priming port
- Pipette slowly
- Leave a tiny bit of buffer in the pipette – this prevents introduction of new air bubbles
- Before 2nd load: open SpotON port

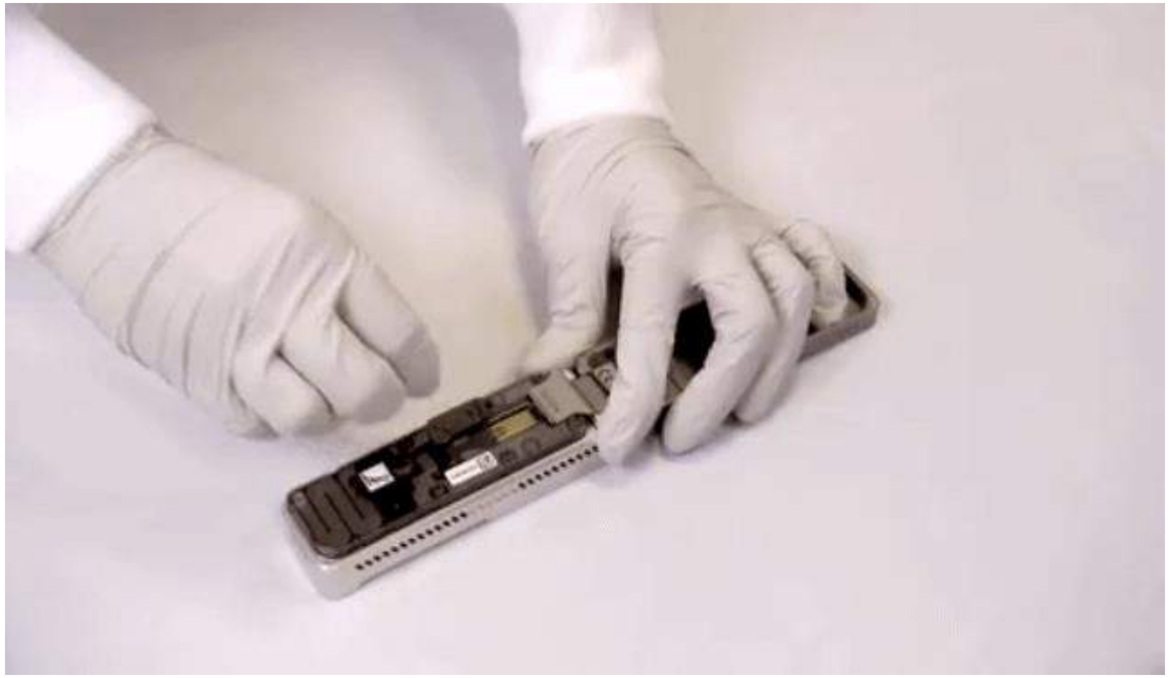

Prepare Flow cell

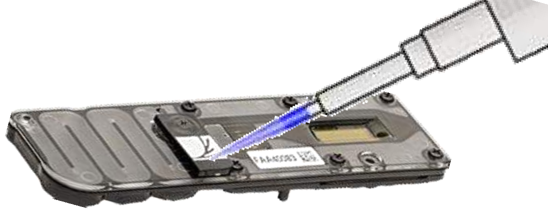

SQB: Sequencing Buffer 37.5 µl SQB

LB: Loading beads 25.5 µl LB (Mix immediately before use)

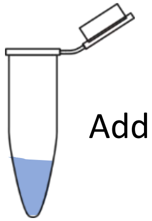

Add to 12 µl of the DNA library

- Mix library by pipetting directly before transfer
- Add 75 µl of the library in a drop wise fashion
- Wait until each drop is gone before adding the next drop
- Close SpotON port and priming port covers

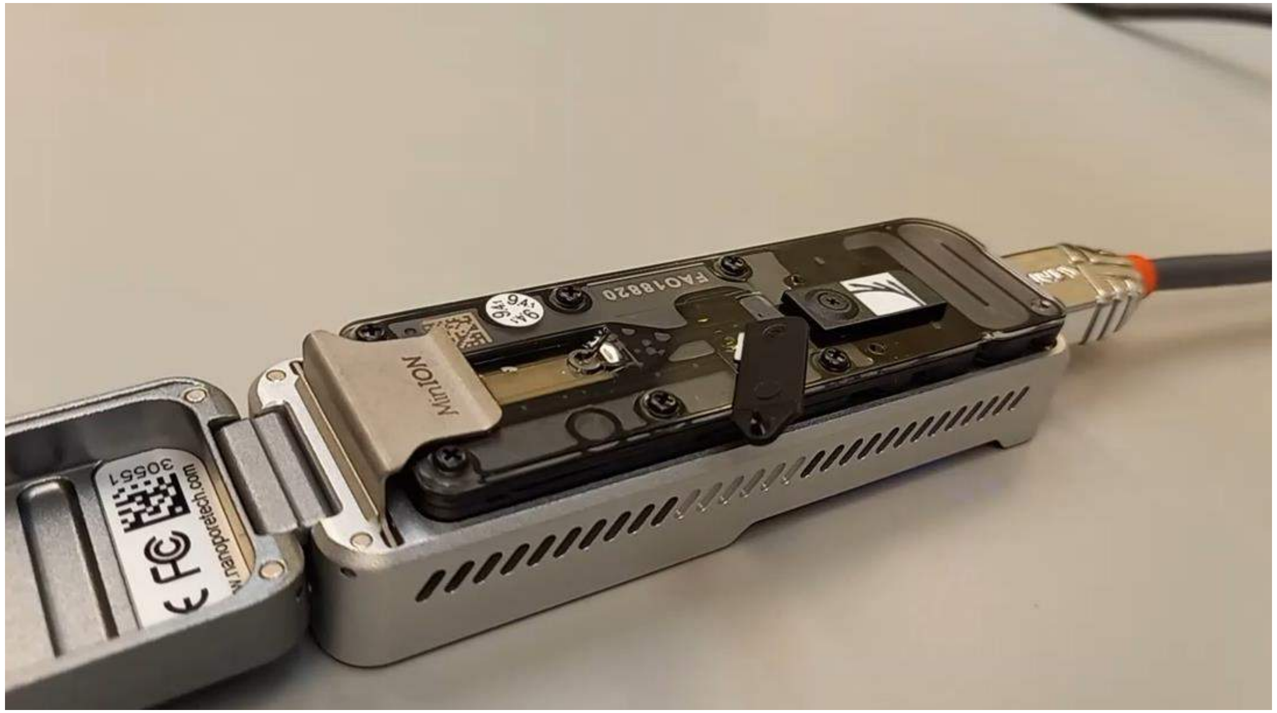

Molecular techniques of vector identification

Sequencing

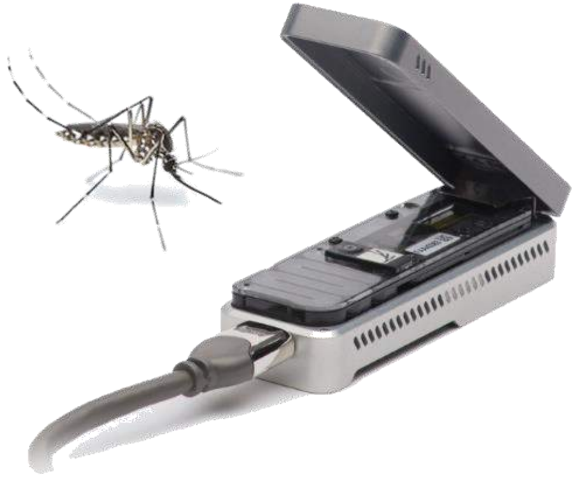

Recap

Where are we?

- We isolated DNA from individual mosquitoes
- We amplified the DNA and marked the sequences with individual indices (tags)
- We prepared the “library”: we ligated sequencing adapters to our DNA and performed several clean-up steps

What’s next?

- We want to start the sequencing run

Starting the sequencing run

- Check if the MinION is connected to the computer and the flow cell is in the MinION

- Check if you have an internet connection to start the sequencing run (you can disconnect once it is running)
- If an internet connection is not possible at all, request the offline version of MinKNOW from nanopore beforehand

- Open the MinKNOW software on your computer

- Install updates if necessary

- Login and chose “Start sequencing”

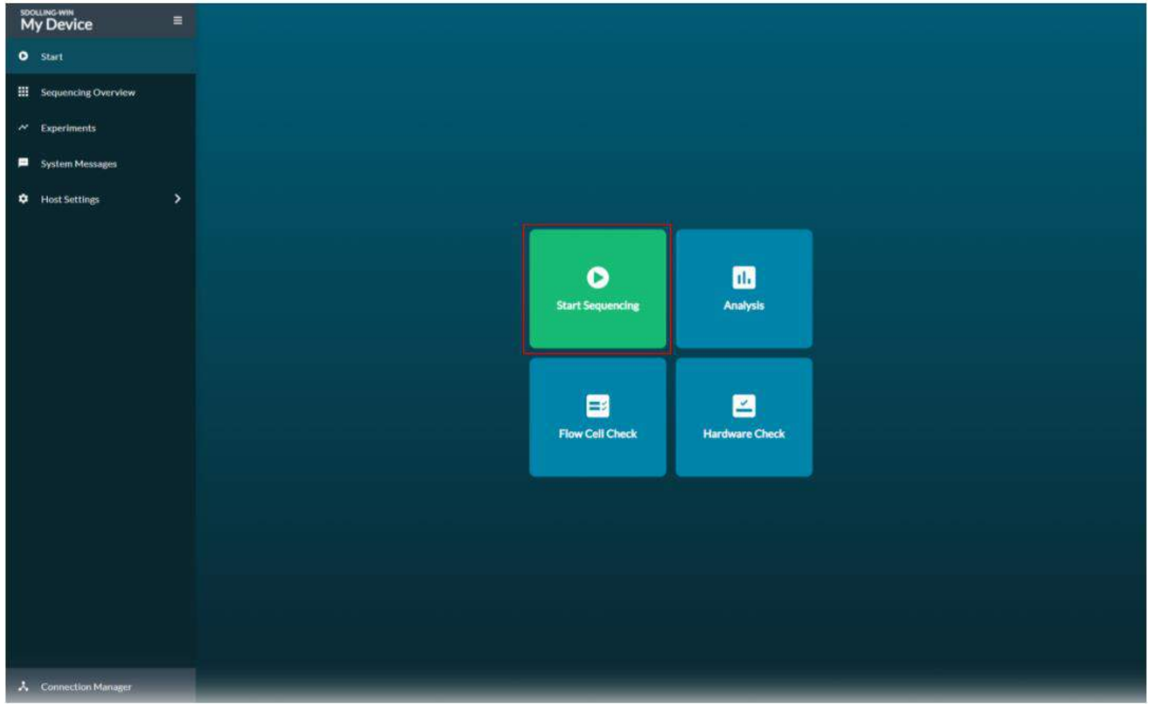

Starting the sequencing run

- Choose a name for the sequencing run
- Pick your flow cell type
- Give a sample ID

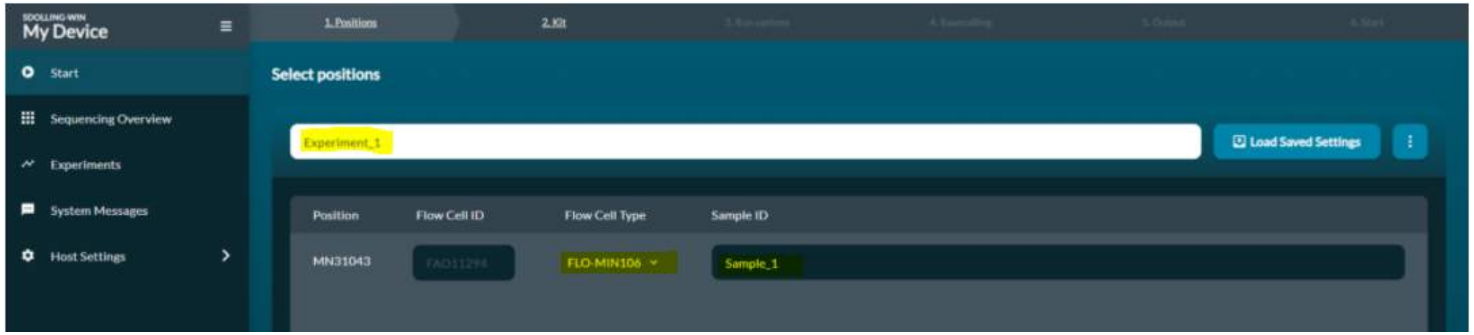

- On the next page, choose the correct sequencing kit
- Also possible to choose extension kits (e.g. for barcoding), but we will skip this

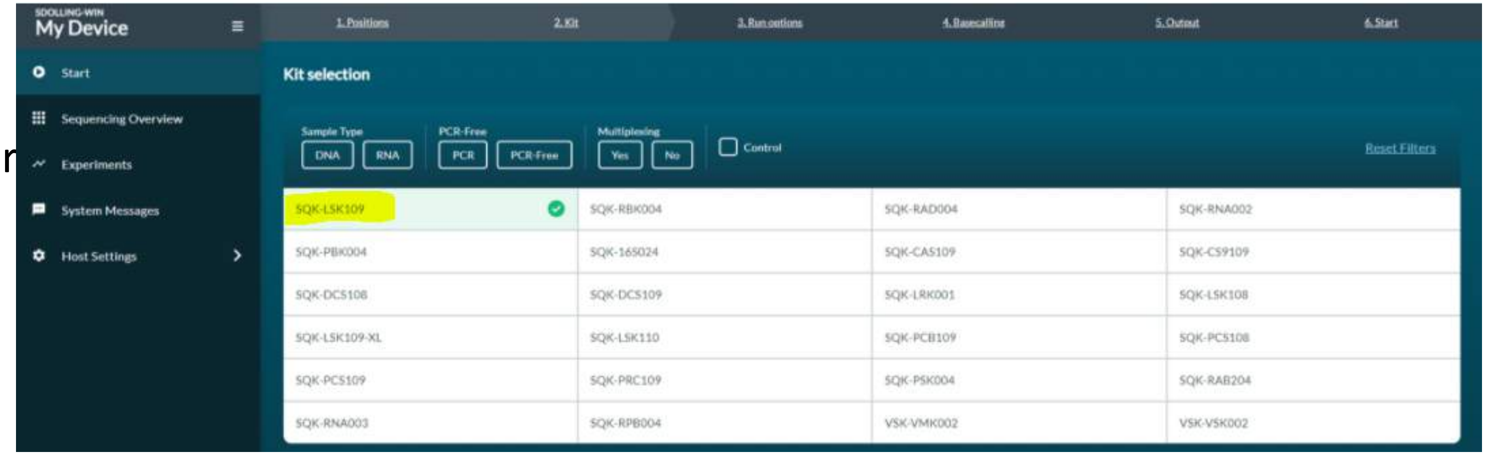

Starting the sequencing run

- Now choose the run options
- Choose the time that you want to run the sequencing for (also possible to end it earlier during the run)
- Leave the voltage as it is

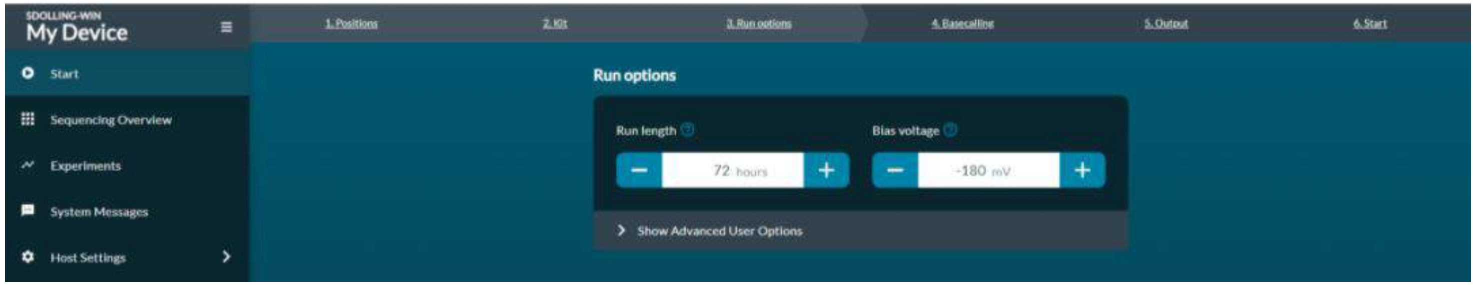

- Choose the basecalling options (I would recommend fast basecall)
- if you run the sequencing offline: basecall not possible
  → either run it when you have an internet connection again
  → or run it offline with Guppy (more on that on the nanopore homepage)

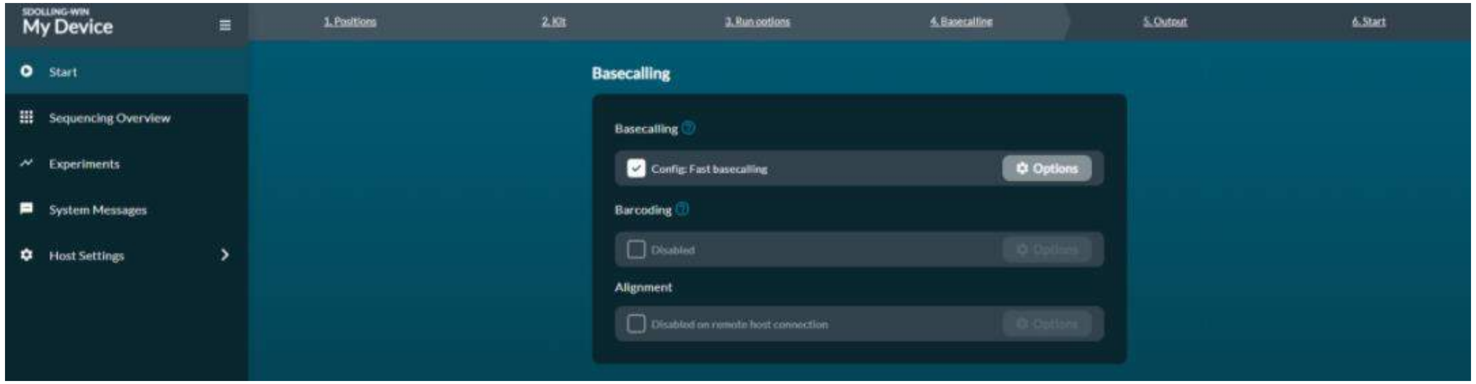

Starting the sequencing run

- Choose the location where data should be saved
- Output format → recommend to save as fast5 and fastq

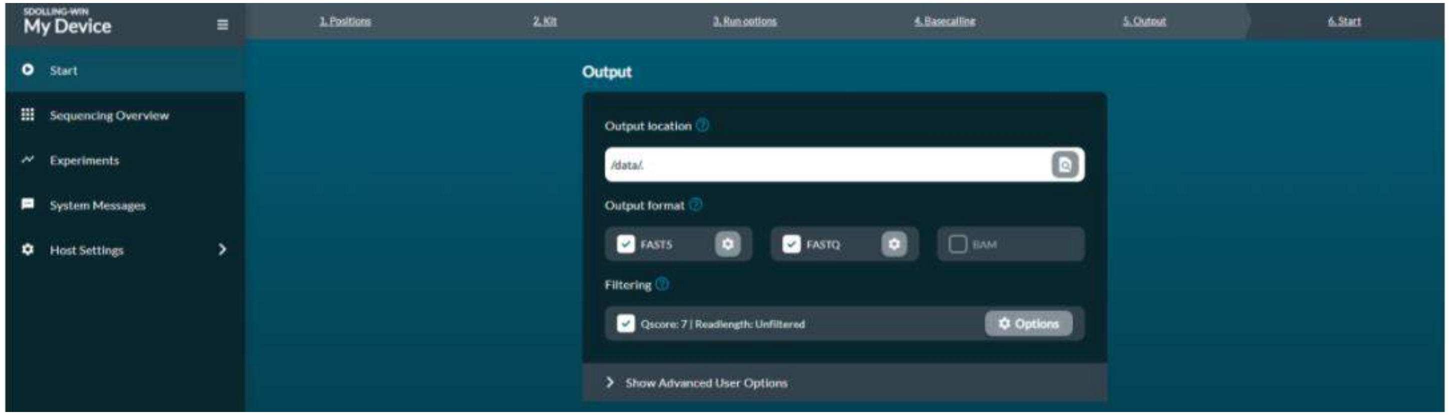

After this you can start the sequencing run

Starting the sequencing run

- MinION will now try to reach its target temperature to start sequencing. This may take a few minutes

- The flow cell will then cycle through the 2048 pores and choose the ¼ pores that work best

- Then sequencing starts

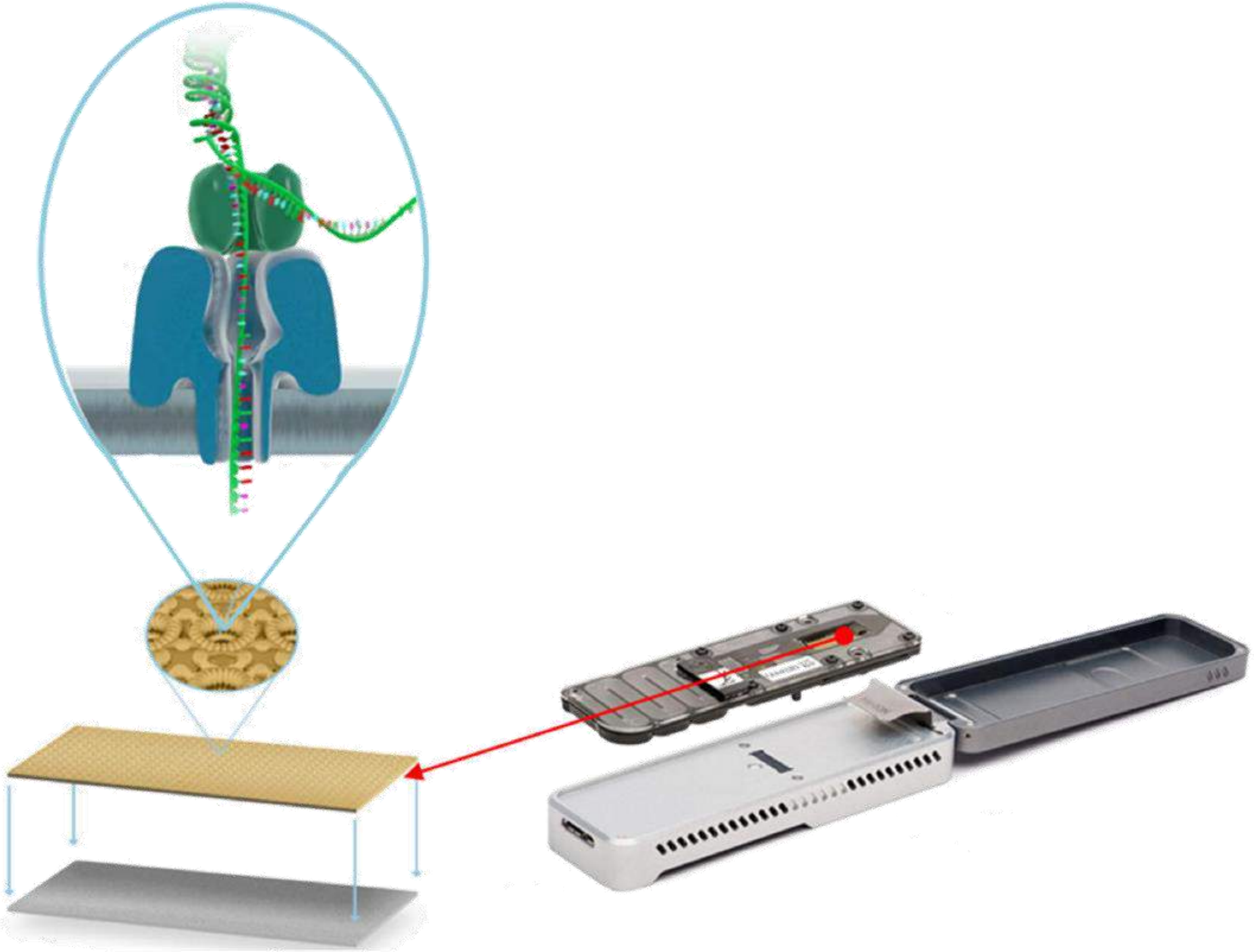

Monitoring the sequencing run

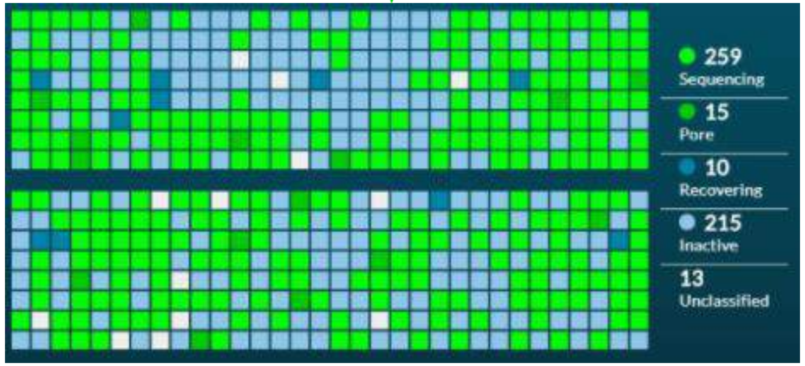

Overview of status of single pores:

- Shows status of currently accessed 512 pores
- Optimal if a lot of pores are bright green (sequencing) plus a few dark green (active)
- A cluster of blue pores could indicate an air bubble on the sensor array

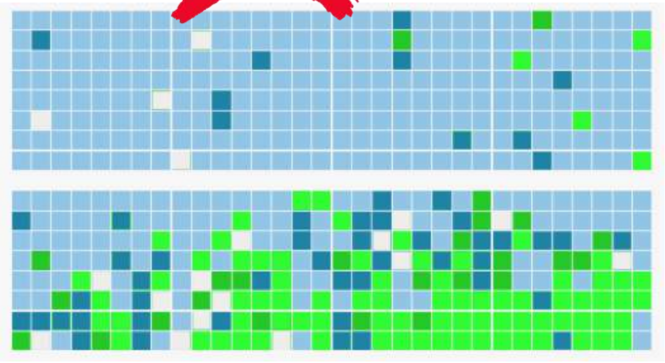

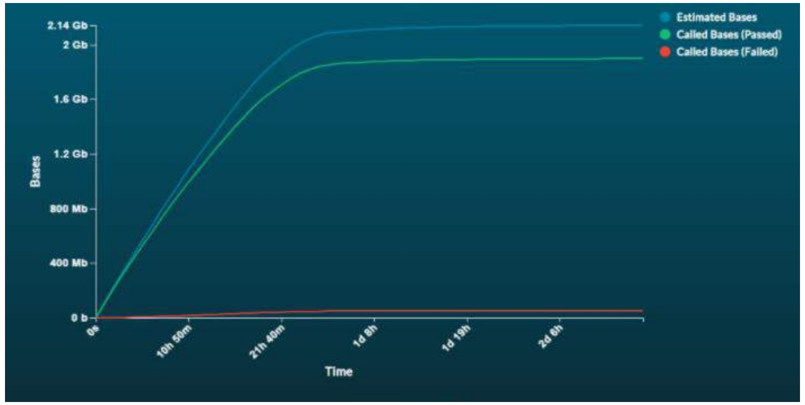

Cumulative output:

- Can be shown for bases and reads
- Number that has been sequenced and basecalled

Monitoring the sequencing run

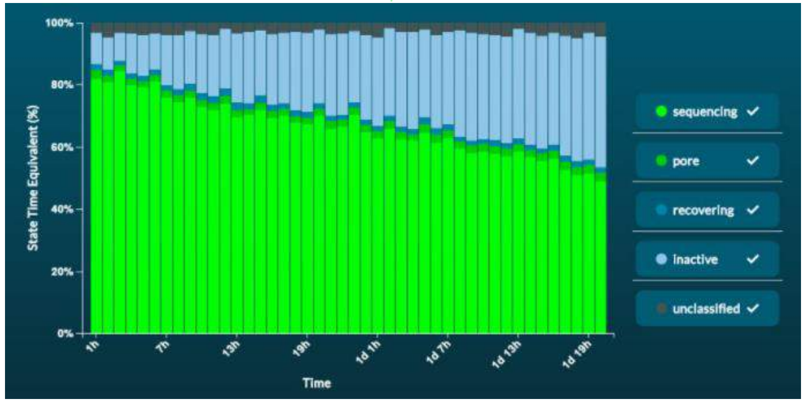

Duty time:

- Summarizes the state of the pores over time
- Colors are the same as in the status graph
- Performance of flow cell will decline over time

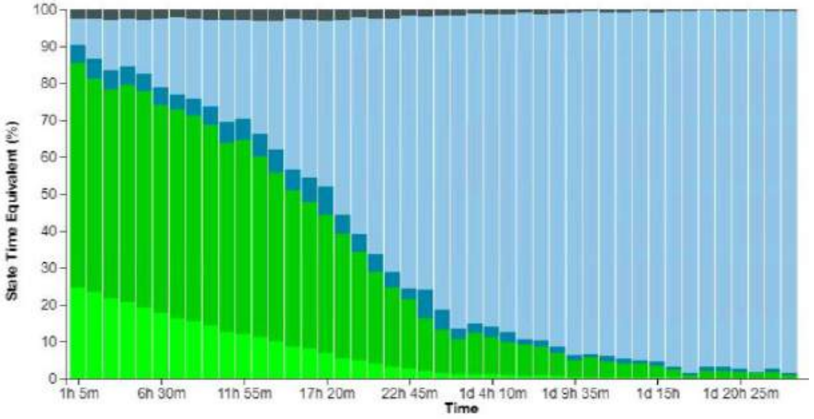

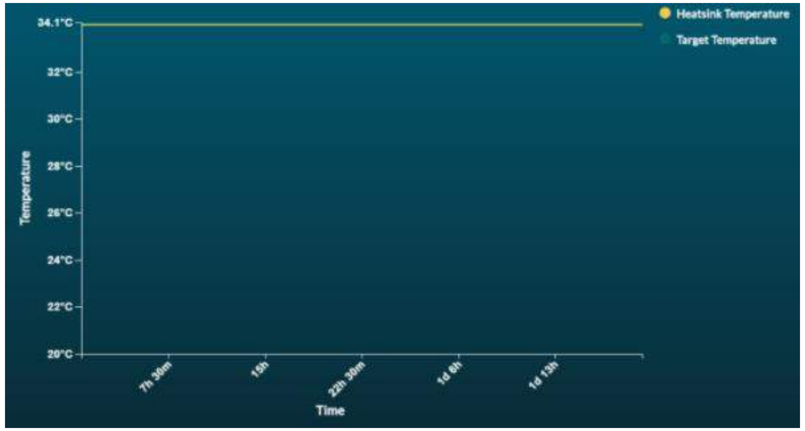

Temperature curve:

- Overview of temperature of the flow cell
- Should be stable over the complete sequencing time
- Check if the environment could cause temperature fluctuations

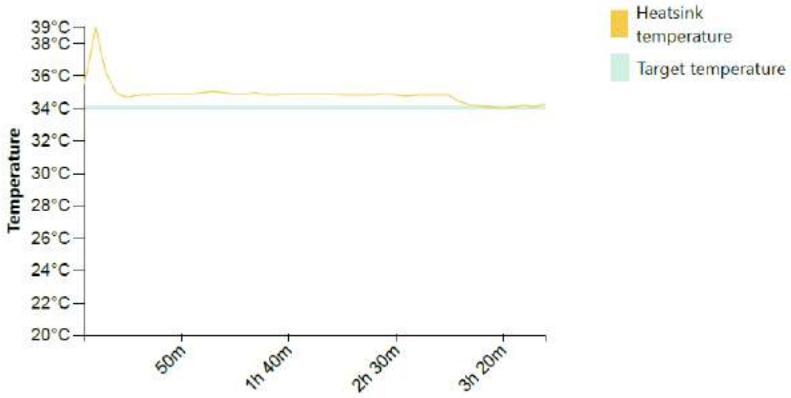

Monitoring the sequencing run

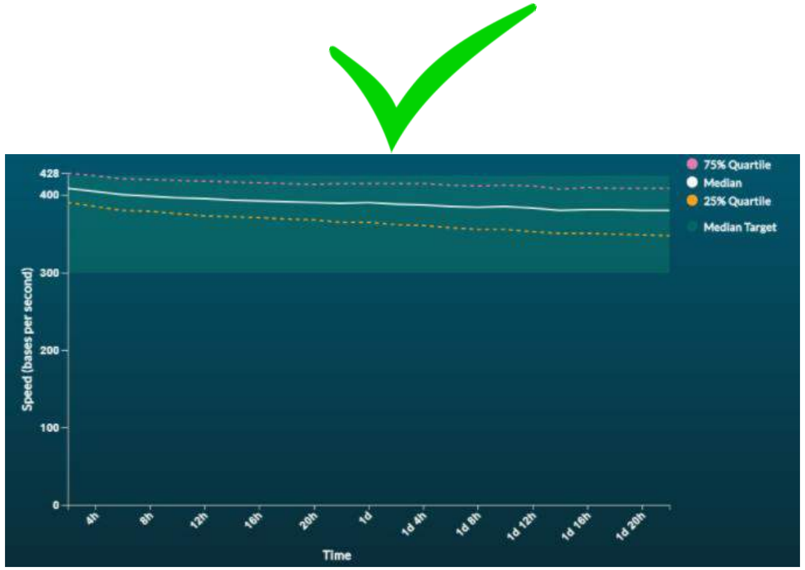

<u>Translocation speed:</u>

- Shows performance of flow cell
- Should stay within the green target range
- Drop of translocation speed: often because too much DNA has been loaded into flow cell
- Increase: often because the temperature is too high

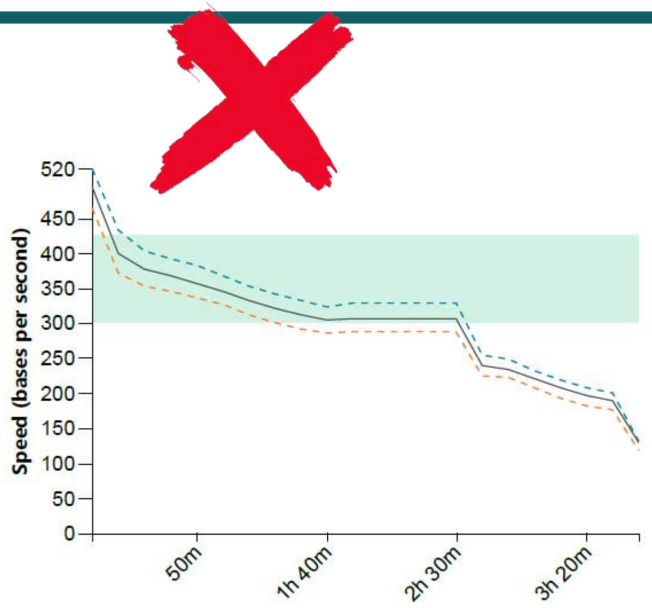

Recap

Where are we?

- We isolated DNA from individual mosquitoes
- We amplified the DNA and marked the sequences with individual indices (tags)
- We prepared the “library”: we ligated sequencing adapters to our DNA and performed several clean-up steps
- We sequenced our DNA

What’s next?

- We will analyze our data bioinformatically

Molecular techniques of vector identification

Bioinformatic analysis

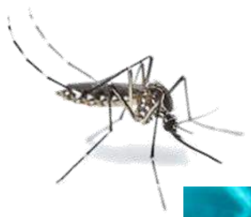

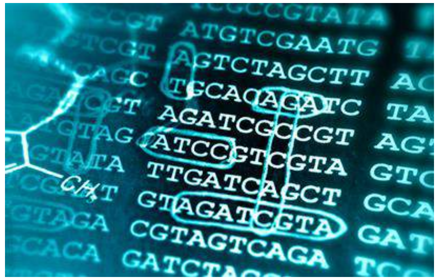

Recap

Where are we?

- We isolated DNA from individual mosquitoes
- We amplified the DNA (still individually)
- During amplification we marked the DNA with individual indices (tags)
- We prepared the DNA for sequencing
- We sequenced the DNA and basecalled the reads

What's next?

- We want to sort the sequences into individuals again
- We want to check which species we find in the dataset

Bioinformatics in general

- Working environment
  - We work with Ubuntu (a Unix distribution)
  - Command line operated

- Environment is perfect to work with large datasets
  - Possible to manipulate multiple files simultaneously
  - Possible to effectively and easily manipulate large datasets (e.g. reformat tables, exchange patterns etc.…)

- We need to learn a few commands, but with those commands we have a lot of power!

Bioinformatic pipeline

What does our data look like now?

Electrical signal → FAST5 file → Basecalling → FASTQ file

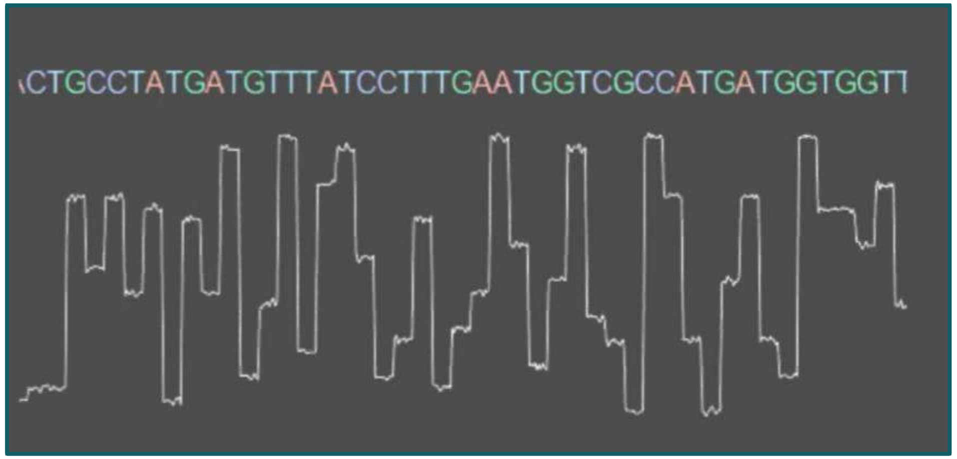

Bioinformatic pipeline

What does our data look like now?

Electrical signal → FAST5 → Basecalling → FASTQ file

nanopore FAST5

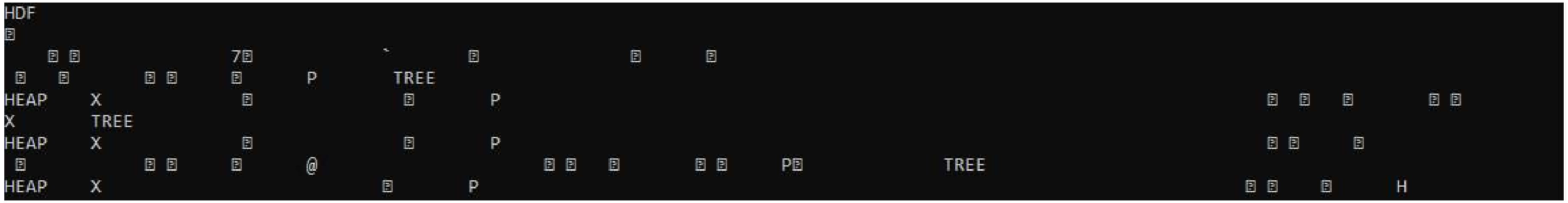

not readable by us

nanopore FASTQ

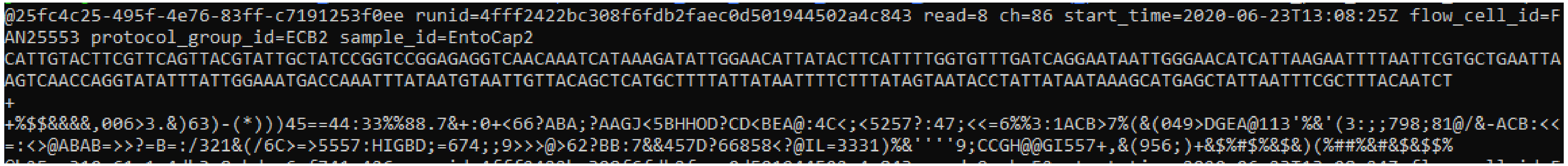

```
@25fc4c25-495f-4e76-83ff-c7191253f0ee runid=4fff2422bc308f6fdb2faec0d501944502a4c843 read=8 ch=86 start_time=2020-06-23T13:08:25Z flow_cell_id=F
AN25553 protocol_group_id=ECB2 sample_id=EntoCap2
CATTGTACTTCGTTCAGTTACGTATTGCTATCCGGTCCGGAGAGGTCAACAAATCATAAAGATATTGGAACATTATACTTCATTTTGGTGTTTGATCAGGAATAATTGGGAACATCATTAAGAATTTTAATTCGTGCTGAATTA
AGTCAACCAGGTATATTTATTGGAAATGACCAAATTTATAATGTAATTGTTACAGCTCATGCTTTTATTATAATTTTCTTTATAGTAATACCTATTATAATAAAGCATGAGCTATTAATTTCGCTTTACAATCT
+
+%$$&&&&,006>3.&)63)-(*)))45==44:33%%88.7&+:0+<66?ABA;?AAGJ<5BHHOD?CD<BEA@:4C<;<5257?:47;<<=6%%3:1ACB>7%(&(049>DGEA@113'%&'(3:;;798;81@/&-ACB:<<
=:<>@ABAB=>>?=B=:/321&(/6C>=>5557:HIGBD;=674;;9>>>@>62?BB:7&&457D?66858<?@IL=3331)%&''''9;CCGH@@GI557+,&(956;)+&$%#$%&$&)(%##%&#&$&$$%
```

readable by us

→ What information does a FASTQ file contain?

Bioinformatic pipeline

What does our data look like now?

Typical Illumina read

```
@K00136:366:HCNWLBBXX:5:1101:25479:1226 2:N:0
AAACTATCACATTTGGCCTCCAAGCCCCCTTGCCCAAAACAATCTCTT
+
AAFFJJAJJJJJJJJJJJJJJJJJJJJJJJJJJJJJJJJJJJJJJJJF
```

1) read identifier/header (always starts with @)
2) The nucleotide sequence
3) + (optional: again the ID)
4) base quality per nucleotide

MinION read (short)

read ID | nanopore run ID | Sample Name | Read number | Pore number | Time of sequencing

```
@d6bf0b89-8fcf-49a3-9449-baff86468732 runid=84acc27b67bd59236a01d86fcc2ebf30e3ab3cec sampleid=AeAnCOIITS read=91 ch=330 start_time=2020-07-14T09:17:27Z
AATTGCAGTTTCTGATTCGTTCTACATGTTGCCACCTTTGGATCAGTATATTGGGTTGAGTGTCACATTCAGAGCTATCTGGTGCAATATTGGATTGGGGTGCTATCTTGGTGGTGGCTGAAGTTATTTGTTTGCCTGTGTGCGGGTTGCTGCC
+
%%&/$$%')2-+*((+/#%%%$%#$$$$$'##$##$$%#%)$&'$%$$%&%(%''&)$#%#$''#$$#$$&$###%##%$%###%&%$%'*(%$&'%($%'"##%%&%()%((%&&'(+&&&(%*$%%'#$$&$$$$%%$&#''-&$%#"'$##
```

MinION read (long)

```
@a93819bc-57c3-4e0f-b9e6-e5f237c4078d runid=4fff2422bc308f6fdb2faec0d501944502a4c843 read=10 ch=255 start_time=2020-06-23T13:08:25Z flow_cell_id
=FAN25553 protocol_group_id=ECB2 sample_id=EntoCap2
CAGTGTACTTCGTTCAGTTACGTATTGCTCTGTCGAGGCGACTAAGCTTCAGGGTGACCAAAATCAAAATAAATGTTGGTATAAAATTGGATCTCCTCCTCTGCTGGATCAAAGAATGAAGTATTTAAATTTCGATCAGTTAAT
AATATTGTAATAGCTCCAGCTAATACGGGTAATGATAATAATAATAAAATAGCAGTAGTACTACAGATCAAACAAATAATGACATTGATCAATGTAATTCCCGATCATATATTAATAACAGTAGTAATAAAATTTACTGCTCTA
AAATCGAAAAATCCCTGCTAAATGTAAAGAAAAAAATAGCTAAATCTACGAAACCCCTGCATGAGCAATTCAGAGAAGATAATGGAGGGTAAACAGTTCATCCGGTTCTGCTCCATTTTCTACTATACTTCTAGAAATAAGAAG
TGTTAAAGAGAGAAGTATTCAAAATCTTATATTATTCGAAAGCTATATCTGGTGCTCTAATATTAATGGAACAAGTCAATTTCCAAATCCTCCAATTATAATATAAGATTTTGAATACTTCCCCCTTCTTTAACACTTCTTATT
TCTAGAAGTATAGTAGAAAATGGAGCAGGAACCGGATGAACTGTTTACCCTCCATTATCTTCTGGAATTGCTCATGCAGGGCTTCAGTAGATTTAGCTATTTTCTTTACATTTAGCAGGATTTCTTCGATTTTGGGAGCGGTAA
ATTTATTACTGTTATTAATAACGATCTCCGGAATCTAGATCGTACCATTGTTTGATCAGTATTACCTTTGTGTTGTATCATTGCGCGCGAACTGCGTATTGGCGATCGAGAACAACTCTTTGATCCGGCGGAAGAAGGAGATCC
CAGTACCGCCGGCATTTATTTTGATTTTTGATCGCCCTGATTTGAATACGCCTCGACAGGCTGTACGTAATC
+
(&&&$'$57:8:=8)%0/.+.$&$&&39=B93.*&%'(&'%2/%''8>>+)2-.450/5129/&447<C?:<A11@A@B?C95;/8698::76@-3124-5(($%.0+'7?@(:9</*,9585@A7435>D970-,17.FB?AE
<C>@>@38@C.C.AC<?=7:9(()**%*-268/&&658834$;=<792/''$%&%,0*55''760/*6=?@8=68=>,-20*('(%%'&%$$%$+&($)%,-3344+5:9:69<=<:5-'+(()06754;7A88711>;9.F/>
@6830+.3&20-.52233/69:76:<>5>DMN>8?;8;8>9;=677+$$%-1&36;760=:@;<6AGCE4@E;;'?5;AFBE@9;<;:58:>@73>>?;9--<'+420/(.;8;=@BJ;CB76<5/0003)1;/334734)+*?
EH>?><-'2%'&%%0447779=?G;;A?@9B>90;.+($#%$'-*(*--32362./73:=CD?@??@;=EA@8;-*(&%).3-3579:;;?)8,-,0:=@@9;8,.(,*(%$''/44;=;;<AA@<>;@==<:8+>BB:I@9<>
.9,-(('&-:9C;(3)8>975'*/'//20BEBC9;CC>?CBICDCC@E@3=2?;<=>89<.8/11.75:,48A:<;668:8:A=40/3-,124299?CCBALC9HIC;CEEFCA<35573KJB<FB+,)13/-#&%%(/$*1&(
().3122+0',,*$%*('%##(./004$0')(*&&$''+)&&$%'*'$%%-/0)(.+&&)(%&%$$$$*)*$$&$&(*'#%)%+#'%%**($$%&&&$$&*./351,)'$%$'##%)'(()22$$1#%*(+%&&&%'$+(*$$-
'%(-'$'$#%%(63343++++*.(++))(+%$%%2320;.43(($###$$&'**)&'$''''&""-/11/(*
```

Bioinformatic pipeline

During basecall, reads are sorted into different categories

```
./
../
drift_correction_FAN25553_4fff2422.csv*
fast5_fail/
fast5_pass/
fast5_skip/
fastq_fail/
fastq_pass/
final_summary_FAN25553_4fff2422.txt*
mux_scan_data_FAN25553_4fff2422.csv*
sequencing_summary_FAN25553_4fff2422.txt*
```

1) fast5 fail → Quality score < 7
2) fast5 pass → Quality score > 7
3) fast5 skip → not basecalled
4) fastq fail
5) fastq pass

Sequences are split up into multiple files with 4000 reads/file

```
FAN25553_pass_4fff2422_0.fastq*
FAN25553_pass_4fff2422_1.fastq*
FAN25553_pass_4fff2422_10.fastq*
FAN25553_pass_4fff2422_11.fastq*
FAN25553_pass_4fff2422_12.fastq*
FAN25553_pass_4fff2422_13.fastq*
FAN25553_pass_4fff2422_14.fastq*
FAN25553_pass_4fff2422_15.fastq*
FAN25553_pass_4fff2422_16.fastq*
FAN25553_pass_4fff2422_17.fastq*
```

This is where the practical part begins!

Bioinformatics in general

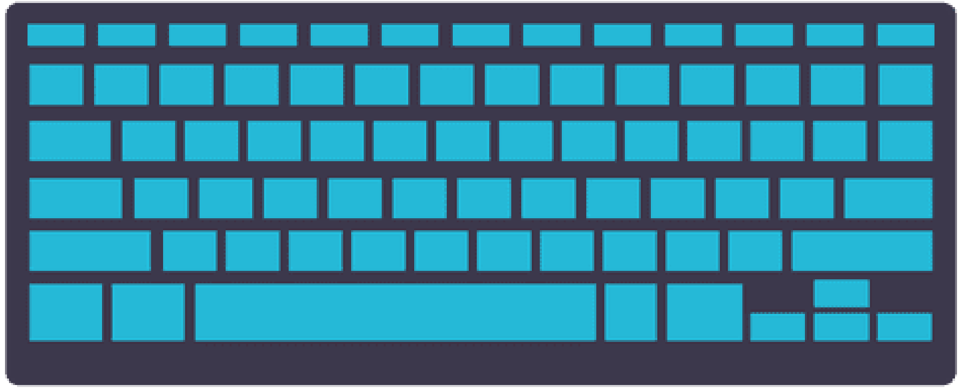

= use command

Bioinformatics in general

- Overview of most important commands:

command	meaning	example		
cd	change directory	cd ..	cd directory	cd ~/directory/subdirectory
ls/ll	list	ls	ls directory	ls ~/directory/subdirectory
cp	copy	cp file ../file		cp file ~/directory/subdirectory/.
mv	move	mv oldfilename newfilename		
head	show first lines	head file	head -n 20 file	
tail	show last lines	tail file	tail -n 20 file	
less	display content of file	less file	-> q	
cat	concatenate (merge together)	cat file1 file2 > file12		
sed	stream editor (manipulates text)	sed ‘s/pattern/newpattern/’ file > newfile		

- Useful:

[STRG]+ C will interrupt the current command

Bioinformatic pipeline

- Navigate to the folder with the sequencing dataset you downloaded yesterday
  - From home directory go 2 steps up

`cd ../..`

  - Then go to the directory "mnt"

`cd mnt`

  - From there navigate to the location of your folder
    e.g.: **`cd c/data/Minion/examples`**

- Check the content of the folder

`ll`

These are the files you should see in the folder:

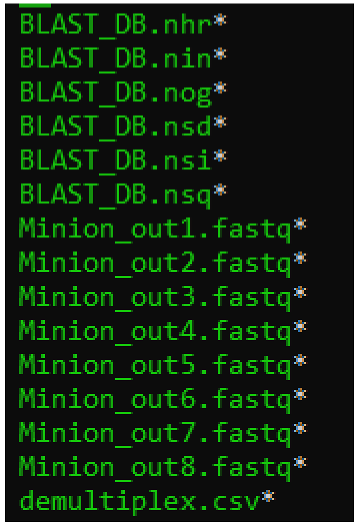

BLAST database we will use for error correction

sequencing reads in FASTQ format

demultiplexing information

Bioinformatic pipeline

Demultiplex file:

SampleID	Tag F	Tag R	Primer F	Primer R
S1_COI	ATCCGGTCGGAGA	TTGCGTCTCACGC	GGTCAACAAATCATAAAGATATTGG	TAAACTTCAGGGTGACCAAAAAATCA
S2_COI	ATCCGGTCGGAGA	GCCGGTCCAAGTG	GGTCAACAAATCATAAAGATATTGG	TAAACTTCAGGGTGACCAAAAAATCA
S3_COI	ATCCGGTCGGAGA	CTGTCGAGGCGAC	GGTCAACAAATCATAAAGATATTGG	TAAACTTCAGGGTGACCAAAAAATCA
S4_COI	ATCCGGTCGGAGA	TCTACTGTTGTGC	GGTCAACAAATCATAAAGATATTGG	TAAACTTCAGGGTGACCAAAAAATCA
S5_COI	ATCCGGTCGGAGA	TGTATATTCAGCG	GGTCAACAAATCATAAAGATATTGG	TAAACTTCAGGGTGACCAAAAAATCA

contains information on Sample ID, primer sequences and tag sequences

miniBarcoder readable format:

```
S1_COI,ATCCGGTCGGAGA,TTGCGTCTCACGC,GGTCAACAAATCATAAAGATATTGG,TAAACTTCAGGGTGACCAAAAAATCA
S2_COI,ATCCGGTCGGAGA,GCCGGTCCAAGTG,GGTCAACAAATCATAAAGATATTGG,TAAACTTCAGGGTGACCAAAAAATCA
S3_COI,ATCCGGTCGGAGA,CTGTCGAGGCGAC,GGTCAACAAATCATAAAGATATTGG,TAAACTTCAGGGTGACCAAAAAATCA
S4_COI,ATCCGGTCGGAGA,TCTACTGTTGTGC,GGTCAACAAATCATAAAGATATTGG,TAAACTTCAGGGTGACCAAAAAATCA
S5_COI,ATCCGGTCGGAGA,TGTATATTCAGCG,GGTCAACAAATCATAAAGATATTGG,TAAACTTCAGGGTGACCAAAAAATCA
```

→ saved as **`demultiplex.csv`**

Bioinformatic pipeline

Demultiplex file:

- Take a look at the file with:

```
less demultiplex.csv
```

- Navigate through file with arrow keys or space bar, exit with “q”

Bioinformatic pipeline

- Combine all FASTQ files into one single file

```
cat *.fastq > Minion_dataset.fastq
```

alternative:

```
cat Minion_out1.fastq Minion_out2.fastq Minion_out3.fastq Minion_out4.fastq … > Minion_dataset.fastq
```

file 1 file 2 file 3 file 4

* = wild card 1: if used as *.fastq the command will use all files that end in .fastq

- Check what your command did by looking at the content of the directory

```
ll
```

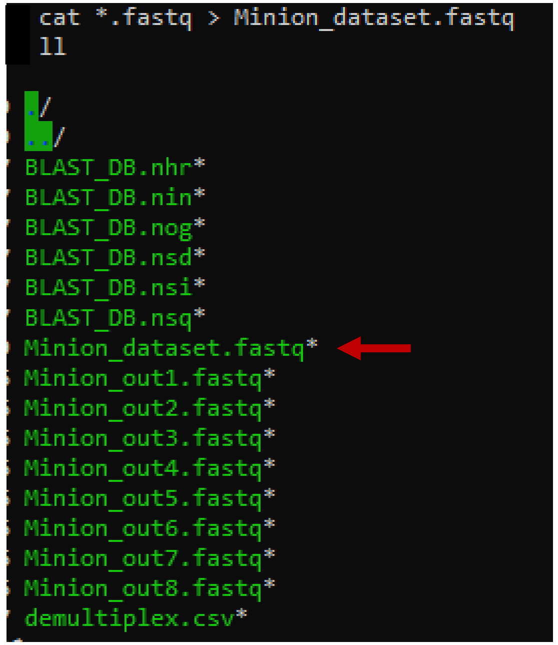

- Create a new directory where we can run all analyses safely

```
mkdir analysis
```

Bioinformatic pipeline

- Copy all needed files into that directory

```
cp BLAST* analysis/.
cp Minion_dataset.fastq analysis/.
cp demultiplex.csv analysis/.
```

!

Use the tab key to autocomplete file names and directory names!

- Move into the new directory and check the content

```
cd analysis
ll
```

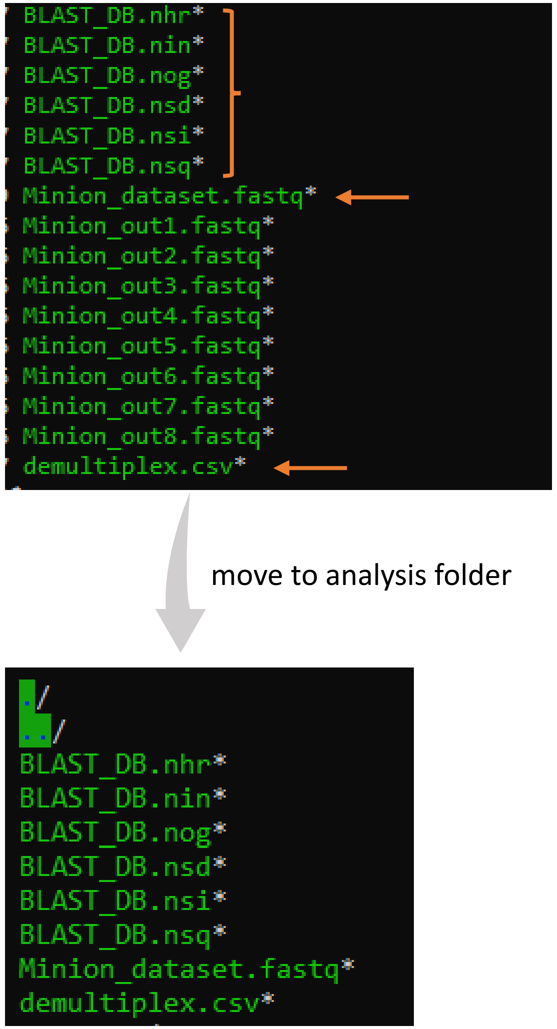

Bioinformatic pipeline

FASTQ

```
1 @HISEQ:468:CA15DANXX:7:1101:1327:1952 1:N:0
2 NGATGTATTACGATGTAAATAATCTGTACGGTTGGGCGATGTGCGAACCGTT
3 +
4 #<<ABFGGGGGGFGGGGGGGGGGGGGGDGGGFGGGGGGEGGGGGGGGGGGGG
1 @HISEQ:468:CA15DANXX:7:1101:1494:1957 1:N:0
2 NATAATTCTATCGTAAAGTCTCTTTGAAATATACTTACAATATGTGATTATT
3 +
4 #=<ABFGGGCGGGGGGGGGGGGGGGGGGFGGGGGGGGGGGGGGGGGDGDGGG
```

FASTA

```
1 >HISEQ:468:CA15DANXX:7:1101:1327:1952 1:N:0
2 NGATGTATTACGATGTAAATAATCTGTACGGTTGGGCGATGTGCGAACCGTT
1 >HISEQ:468:CA15DANXX:7:1101:1494:1957 1:N:0
2 NATAATTCTATCGTAAAGTCTCTTTGAAATATACTTACAATATGTGATTATT
```

- Reformat the file from FASTQ to FASTA format (delete the quality scores)

```
sed -n ‘1~4s/^@/>/p;2~4p’ Minion_dataset.fastq > Minion_dataset.fasta
```

```
sed ‘s/pattern/newpattern/’ file > newfile
```

```
1~4    ⟶ split file into 4-line blocks, the following statement is true for line 1
s///   ⟶ substitute the following pattern for a new pattern
^@/>   ⟶ look for @ at the beginning of a line, exchange with >
p      ⟶ now print this line with the modifications
2~4p   ⟶ print the second line of the 4-line blocks
```

Bioinformatic pipeline

1 Demultiplexing (separating) reads

2 Correcting barcodes

3 Correction 2

4 Building consensus

5 BLAST

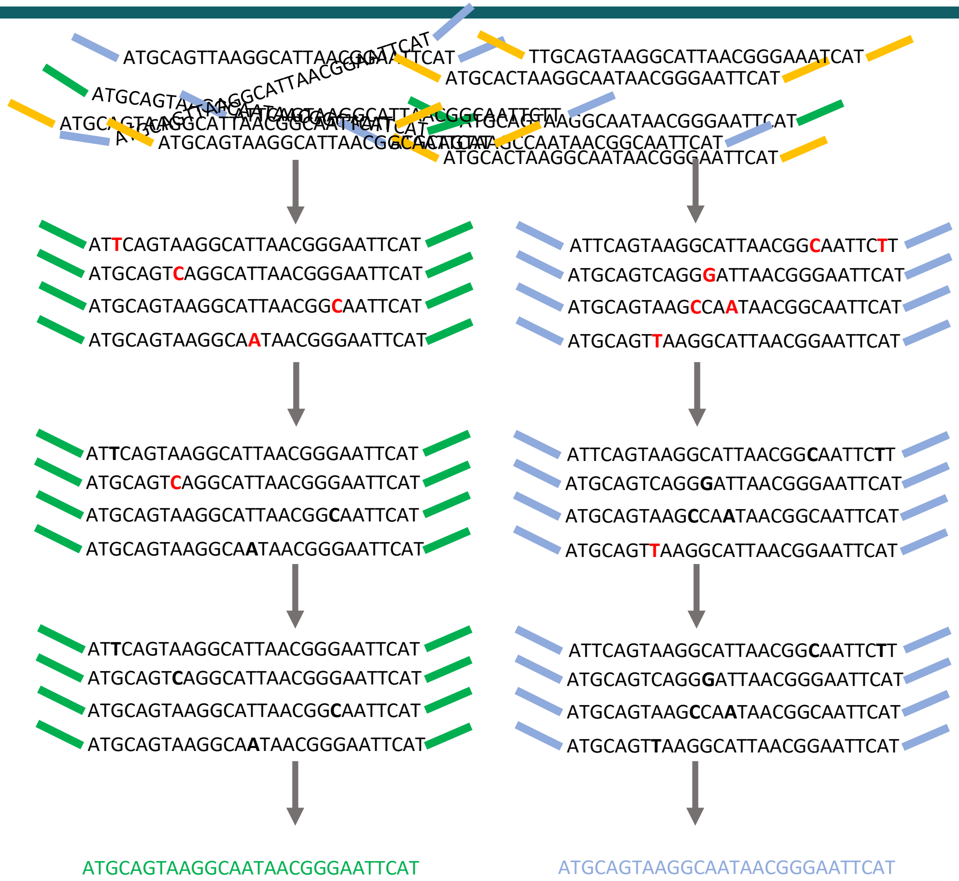

Bioinformatic pipeline

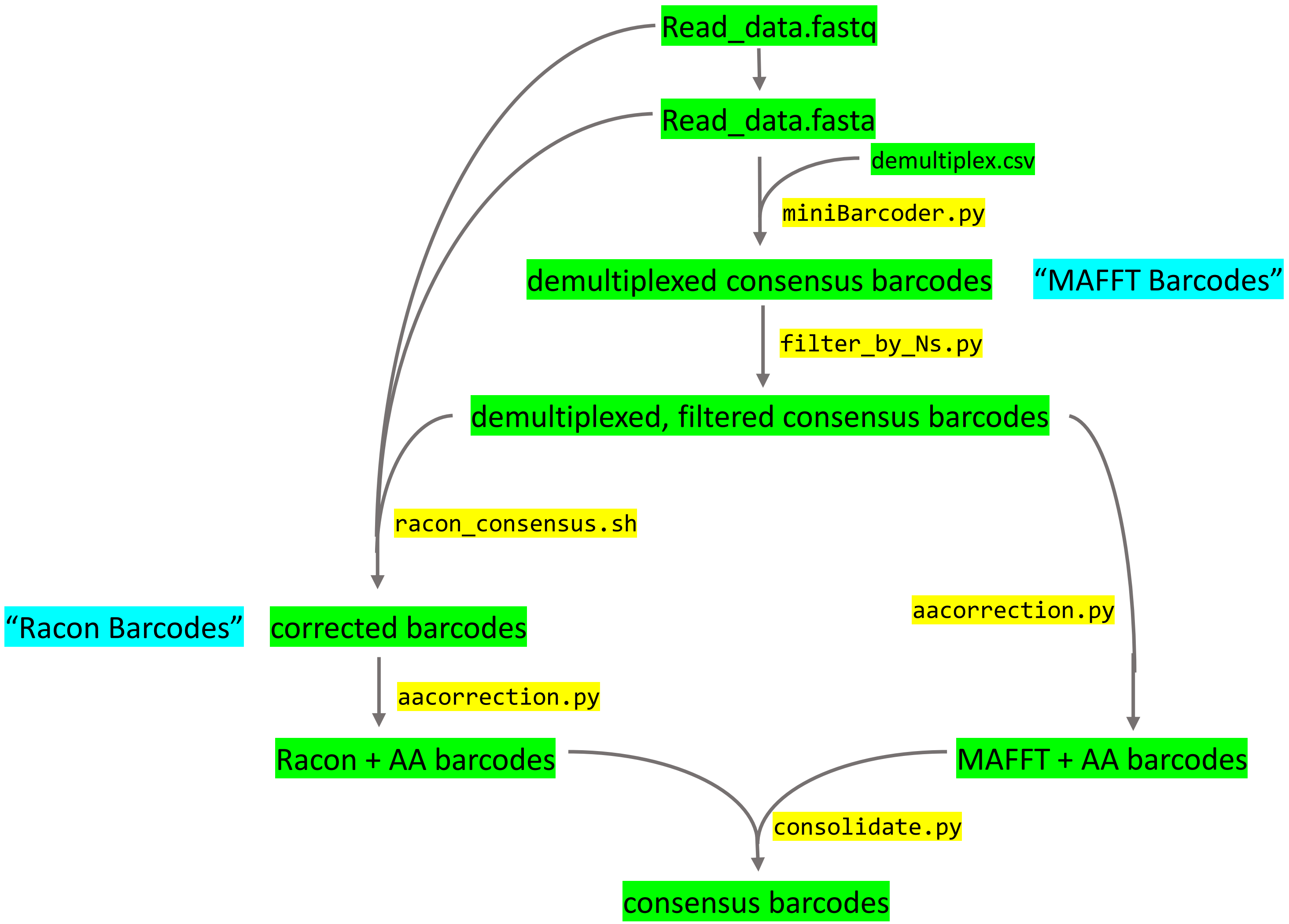

Bioinformatic pipeline

- Activate the conda environment that we installed

`conda activate mbconda`

```
juliane@2W8M433:~$ conda activate mbconda
(mbconda) juliane@2W8M433:~$
```

- Use miniBarcoder to demultiplex the read set

`miniBarcoder -h`

```
usage: miniBarcoder.py [-h] -f INFASTA -d DEMFILE -o OUTDIR -l MINLEN
                       [-m MODE] [-mm {0,1,2,3,4,5}] [-e EVALUE] [-g GAPS]
                       [-D MAXDEPTH] [-t THREADS] [-bl BLEN]

Script for obtaining barcodes

optional arguments:
  -h, --help            show this help message and exit
  -f INFASTA, --infasta INFASTA
                        Path to input fasta file
  -d DEMFILE, --demfile DEMFILE
                        Path to demultiplexing file
  -o OUTDIR, --outdir OUTDIR
                        set output directory path
  -l MINLEN, --minlen MINLEN
                        exclude barcode sequences identified that are shorter
                        than specified length
  -m MODE, --mode MODE  run with unique tag mode (1) or dual tag mode (2)
  -mm {0,1,2,3,4,5}, --mismatch {0,1,2,3,4,5}
                        number of mismatches allowed in tags, must be <=5,
                        default 2
  -e EVALUE, --evalue EVALUE
                        evalue for primer search using glsearch36,default 1e+6
  -g GAPS, --gaps GAPS  number of gaps allowed for primer identification,
                        default 5
  -D MAXDEPTH, --maxdepth MAXDEPTH
                        set max depth per coverage to improve speed, default
                        100, must be >2
  -t THREADS, --threads THREADS
                        number of threads for glsearch and mafft
  -bl BLEN, --barcodelength BLEN
                        estimated barcode length, used for unique tag mode
                        only, please keep it slightly shorter than actual
                        barcode length, due to indel errors
```

Bioinformatic pipeline

- Activate the conda environment that we installed

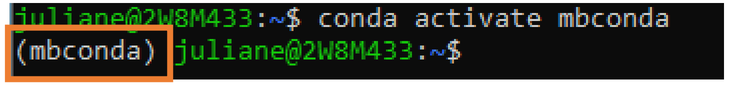


```
conda activate mbconda
```

- Use miniBarcoder to demultiplex the read set

```
miniBarcoder -h
```

```
miniBarcoder -f Minion_dataset.fasta -d demultiplex.csv -o miniBarcoder_out -l 650
```

Bioinformatic pipeline

- Activate the conda environment that we installed

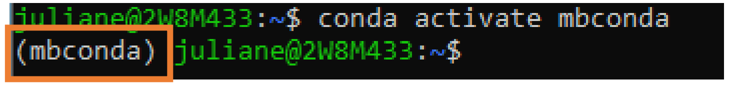


```
conda activate mbconda
```

- Use miniBarcoder to demultiplex the read set

```
miniBarcoder -h
```

```
miniBarcoder -f Minion_dataset.fasta -d demultiplex.csv -o miniBarcoder_out -l 650 -t 4
```

- Check what your command did by looking at the content of the directory

```
ll
```

- Take a look into the newly created directory with the miniBarcoder output

```
ll miniBarcoder_out
```

Bioinformatic pipeline

- Activate the conda environment that we installed

conda activate mbconda

- Use miniBarcoder to demultiplex the read set

miniBarcoder -h

miniBarcoder -f Minion_dataset.fasta -d dem

- Check what your command did by looking at the con

ll

- Take a look into the newly created directory with the

ll miniBarcoder_out

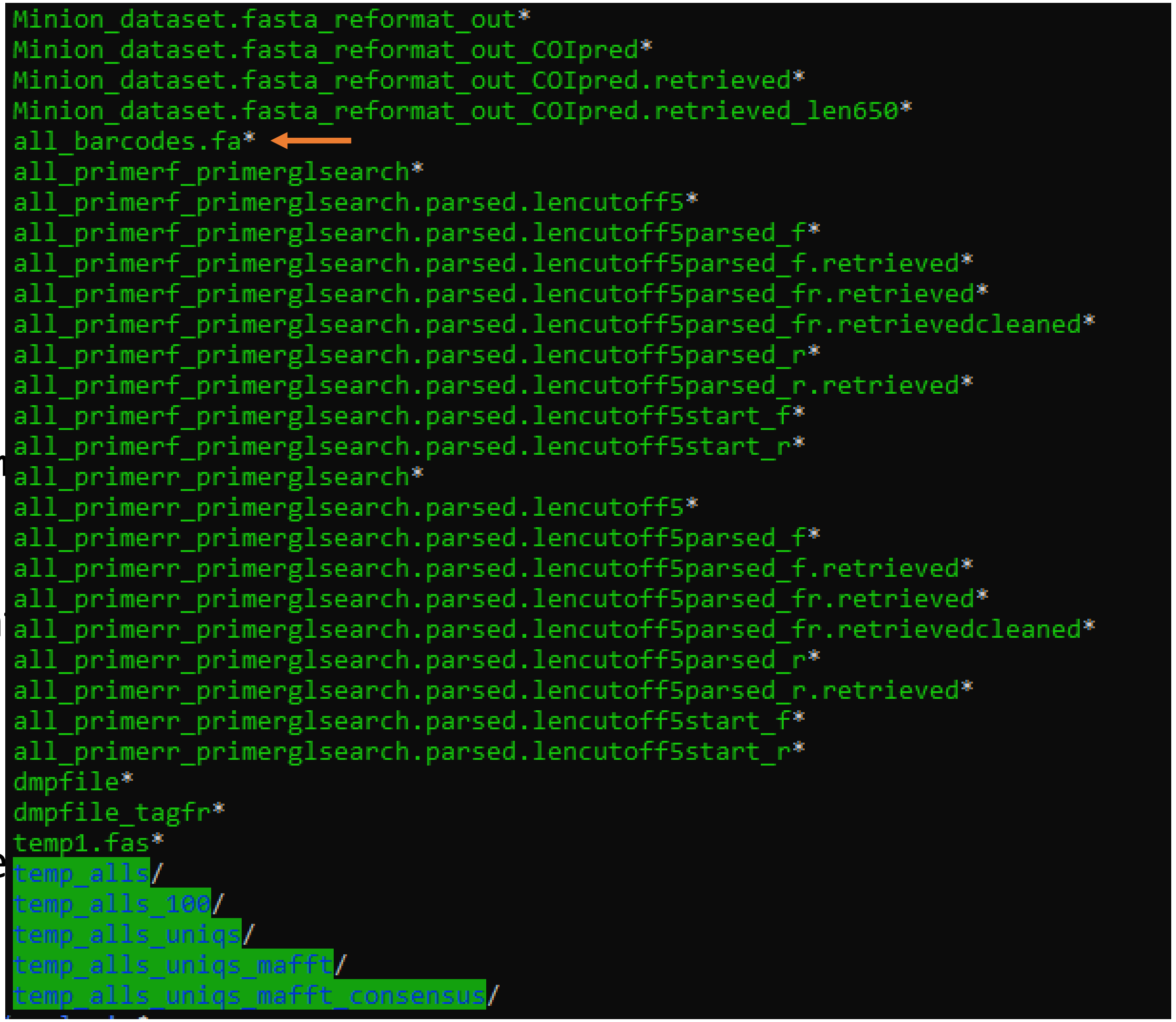

Bioinformatic pipeline

- Look at the file with the demultiplexed barcodes

```
less miniBarcoder_out/all_barcodes.fa
```

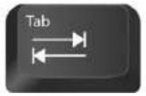

!

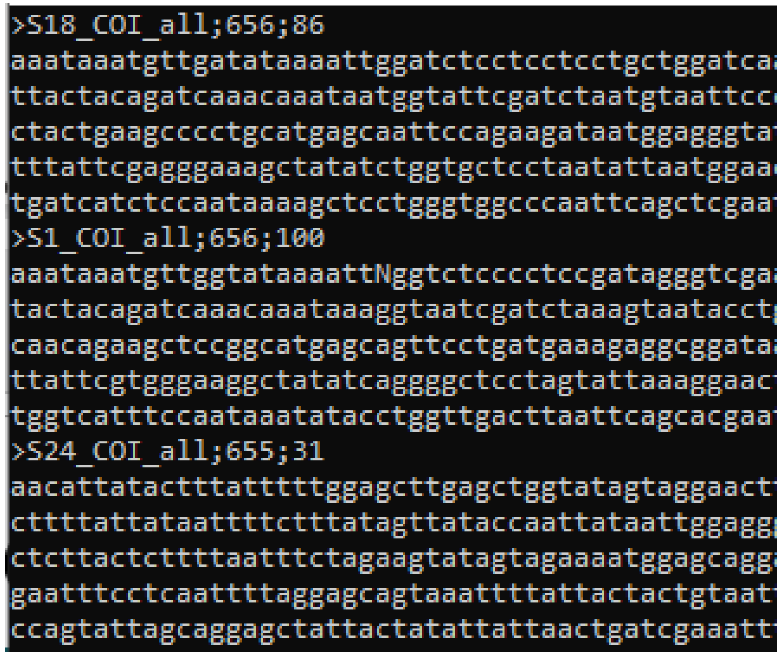

- Rename the barcoding file and copy it from the miniBarcoder directory to the current directory

```
mv miniBarcoder_out/all_barcodes.fa MAFFT_barcodes.fa
```

Bioinformatic pipeline

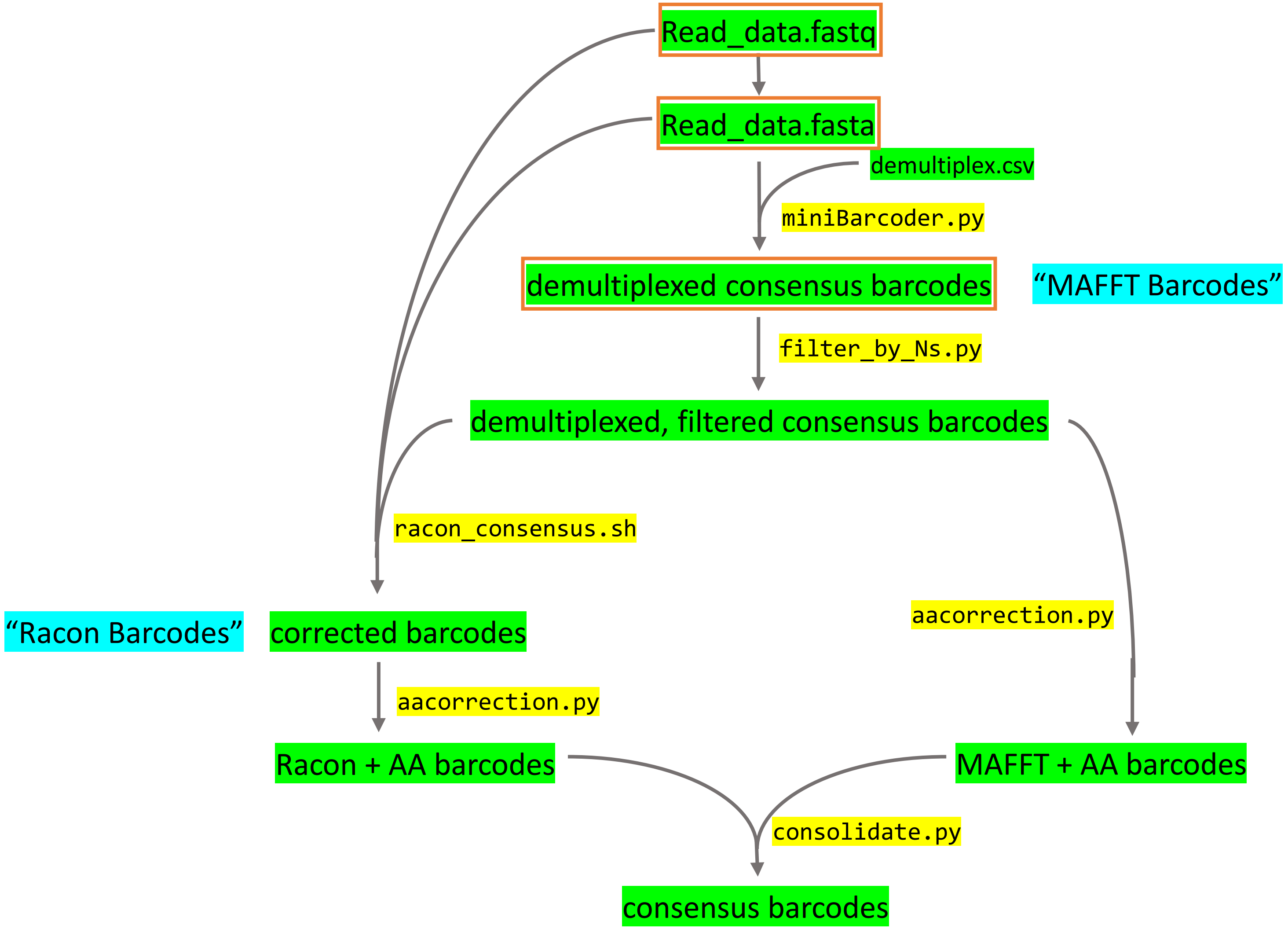

Srivathsan et al. 2019; BMC

Bioinformatic pipeline

- Filter reads that contain a lot of ambiguous sites (“N”s). They are not informative for us

```
filter_by_Ns.py -h
```

```
filter_by_Ns.py -i MAFFT_barcodes.fa –n 6
```

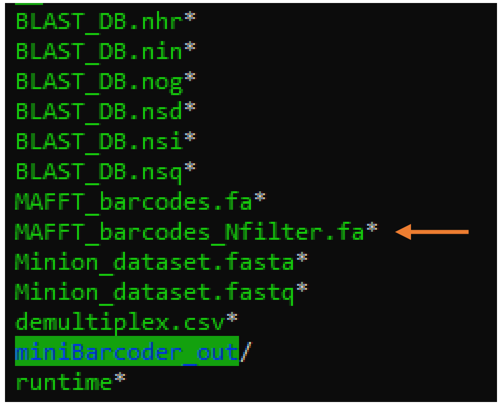

- Check what your command did by looking at the content of the directory

```
ll
```

Bioinformatic pipeline

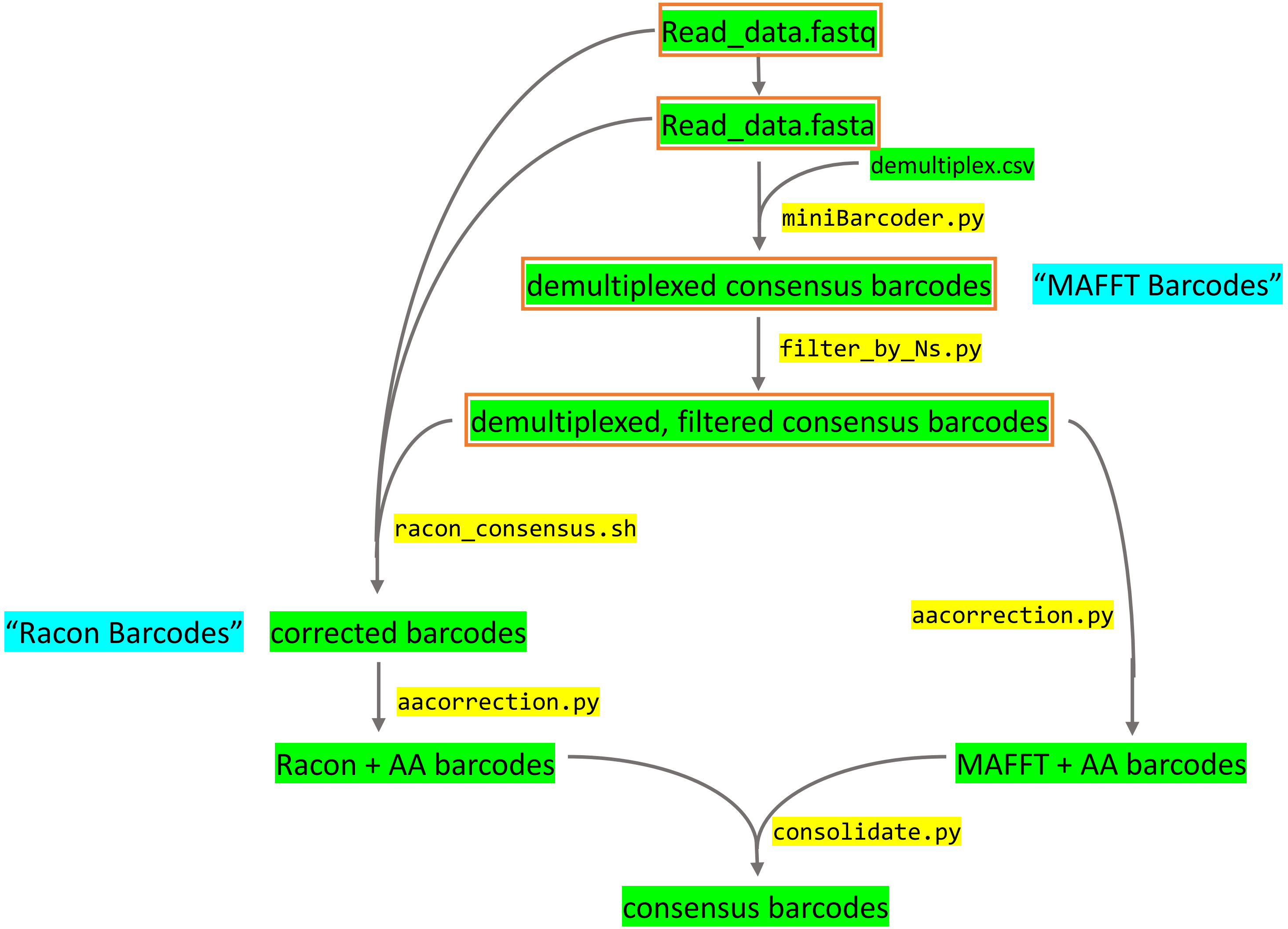

Srivathsan et al. 2019; BMC

Bioinformatic pipeline

- Correct barcodes again. This time we use the program “Racon”

```
racon_consensus.sh Minion_dataset.fastq Minion_dataset.fasta miniBarcoder_out MAFFT_barcodes.fa RACON_barcodes
```

- Check if the command worked

```
ll
```

Bioinformatic pipeline

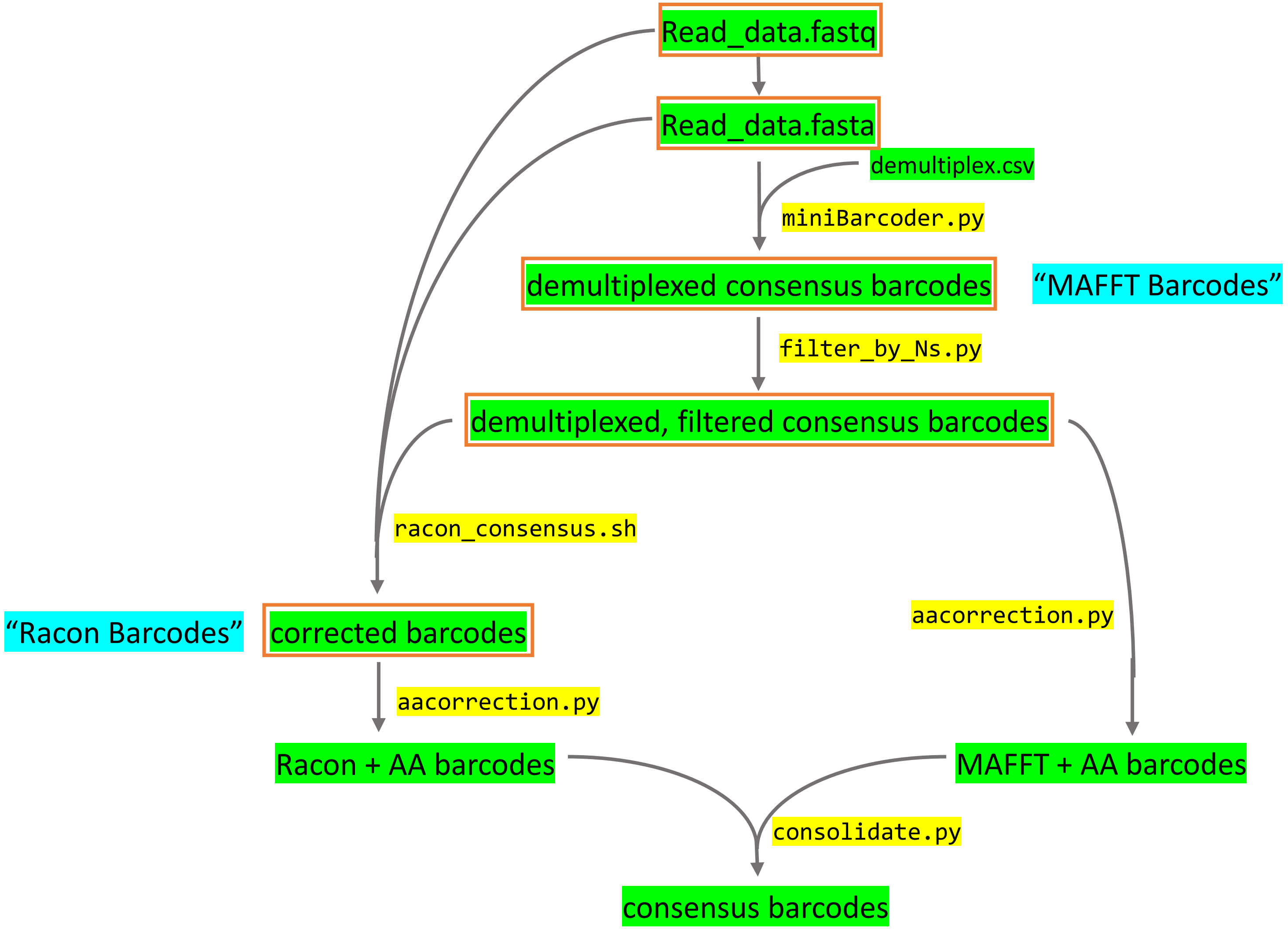

Bioinformatic pipeline

- We will now run the amino acid correction. This step is a second correction step aimed at targeting frame shifts

Frameshift:

- caused by insertions or deletions
- MinION sequencing: indels can happen in homopolymeric regions (repetition of the same base)

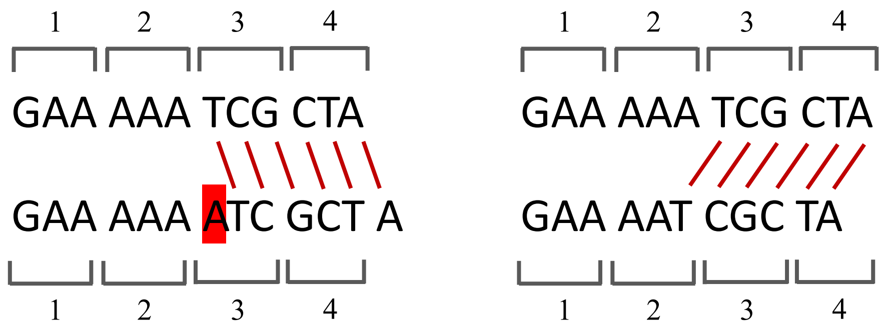

comparison to other sequences via BLAST

→ identification of frame shifts due to indels

→ correction via conserved AA motifs

- BLAST step: identification of best hits → alignment → identification of correct reading frame
- Correction of mismatches

Bioinformatic pipeline

- We will now run the amino acid correction. This step is a second shifts

aacorrection.py -h

a) online version → Online BLAST database
Really slow
Makes sense in the beginning

b) offline version → Local database
Fast
Makes sense when you have established the database

```
usage: aacorrection.py [-h] -b INFASTA -o OUTFILE [-p THREADS]
                       [-bo BLASTOUTFILE] [-bf BLASTACCFILE] [-d PATH_TO_DB]
                       [-a NAMBS] [-l MINLEN] [-L MAXLEN] [-c CONGAPS]
                       [-n NAMINO] [-g GENCODE] [-e EVALUE] [-H HPLEN]
                       [-s SUPPORT]

Script for correcting barcodes using conserved amino acids

optional arguments:
  -h, --help            show this help message and exit
  -b INFASTA, --barcodes INFASTA
                        Path to input barcode fasta file
  -o OUTFILE, --outfile OUTFILE
                        outfile file name
  -p THREADS, --threads THREADS
                        number of threads for BLAST,default=4
  -bo BLASTOUTFILE, --blastout BLASTOUTFILE
                        Path to blast output file, outputformat 6
  -bf BLASTACCFILE, --blastfasta BLASTACCFILE
                        Path to fasta file containing sequences of BLAST hits,
                        required if -bo or --blastout is given
  -d PATH_TO_DB, --db PATH_TO_DB
                        Path to nucleotide database with database prefix, if
                        local copy is unavailable you can try typing 'nt
                        -remote'. note that remote has not been extensively
                        tested and is slower
  -a NAMBS, --amb NAMBS
                        proportion of ambiguities allowed per barcode,
                        default=0.01
  -l MINLEN, --minlen MINLEN
                        exclude barcodes shorter than this length, default=640
  -L MAXLEN, --maxlen MAXLEN
                        exclude barcodes longer than this length, default=670
  -c CONGAPS, --congaps CONGAPS
                        exclude sequences containing any gap of length >=
                        value, default=5
  -n NAMINO, --namino NAMINO
                        number of flanking amino acids around the gap used for
                        correction, default=3
  -g GENCODE, --gencode GENCODE
                        genetic code https://www.ncbi.nlm.nih.gov/Taxonomy/Uti
                        ls/wprintgc.cgi, default=5, invertebrate mitochondrial
  -e EVALUE, --evalue EVALUE
                        e-value for BLAST search, default=1e-5
  -H HPLEN, --hplen HPLEN
                        minimum homopolymer length, default=2
  -s SUPPORT, --support SUPPORT
                        minimum support for indel in references. Reducing this
                        increases chances of errors and improper detection of
                        reading frames
```

Bioinformatic pipeline

- We will now run the amino acid correction. This step is a second correction step aimed at targeting frame shifts

```
aacorrection.py -h
```

```
aacorrection.py -b RACON_barcodes.fa -d BLAST_DB -o RACON_aacorr_barcodes_Nfilter.fa
```

Bioinformatic pipeline

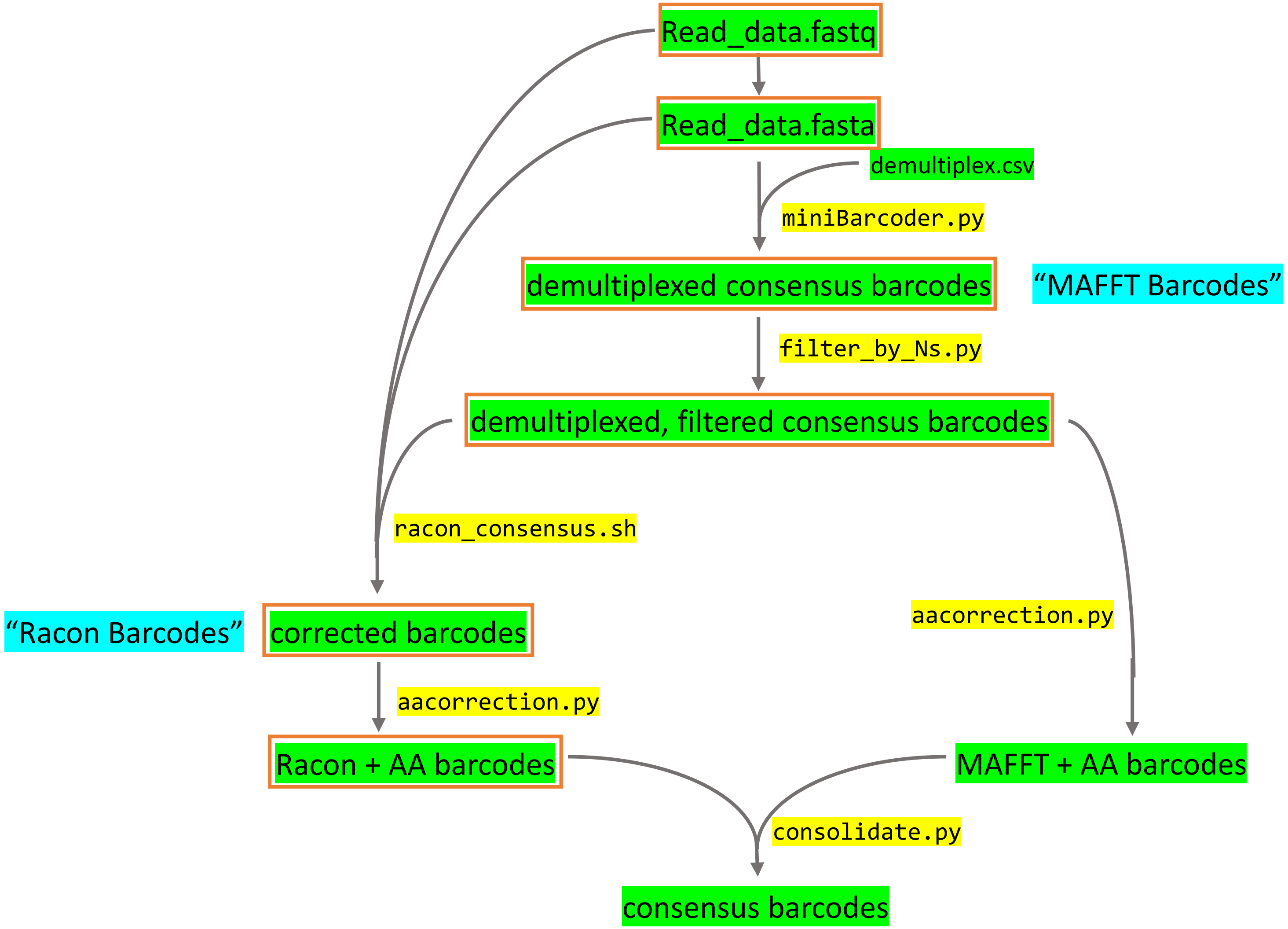

Bioinformatic pipeline

- We will now run the amino acid correction for the MAFFT barcodes

```
aacorrection.py -b MAFFT_barcodes_Nfilter.fa -d BLAST_DB -o MAFFT_aacorr_barcodes_Nfilter.fa
```

Bioinformatic pipeline

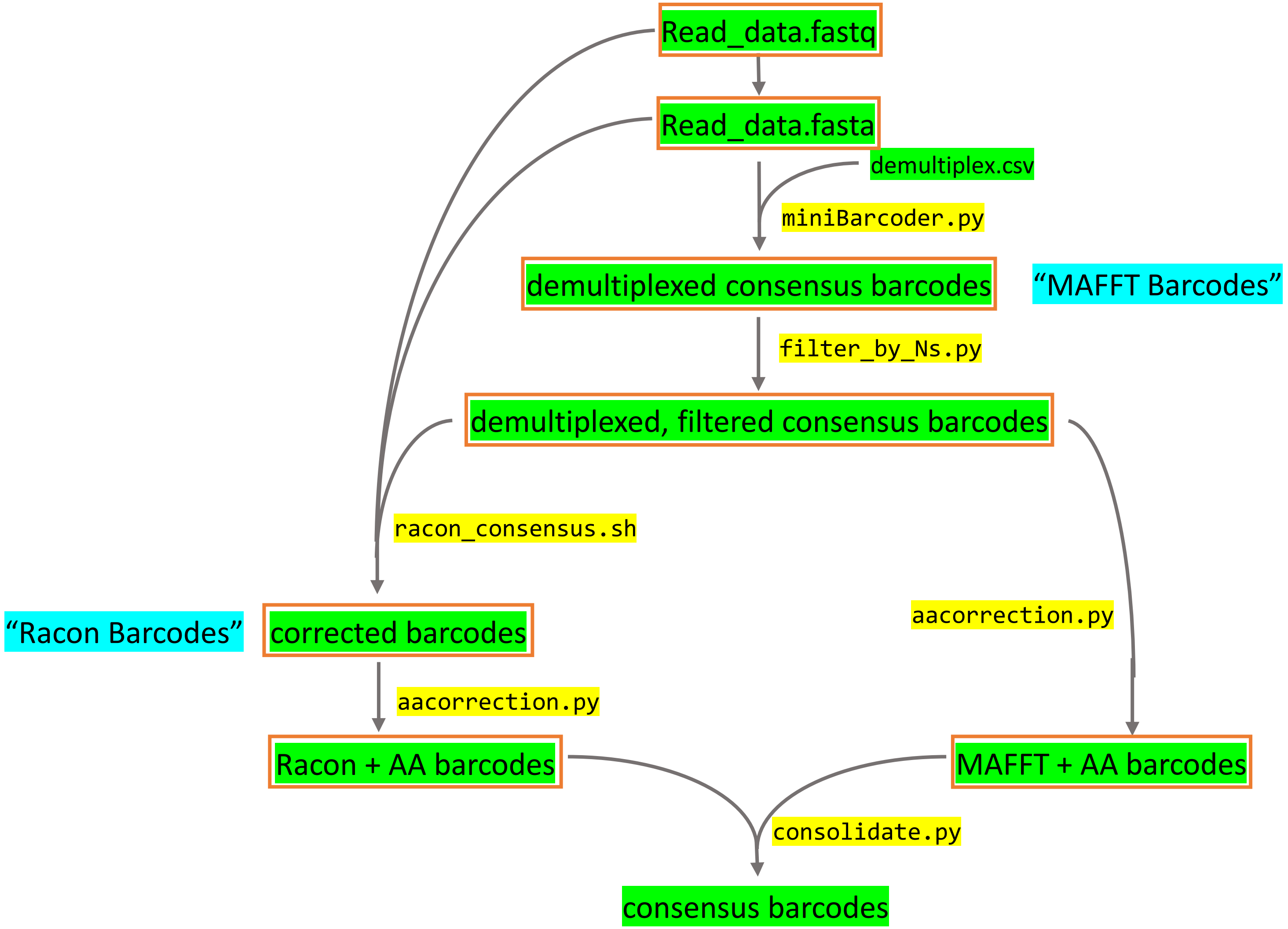

Bioinformatic pipeline

- Merging of the two different corrected barcode files

consolidate.py -h

```
usage: consolidate.py [-h] -m MAFFTB -r RACONB -o OUTFILE -t TEMP

Script for obtaining consolidated barcodes from MAFFT+AA and RACON+AA barcodes

optional arguments:
  -h, --help            show this help message and exit
  -m MAFFTB, --mafft MAFFTB
                        Path to input mafft corrected barcode fasta file
  -r RACONB, --racon RACONB
                        Path to input racon corrected barcode fasta file
  -o OUTFILE, --outfile OUTFILE
                        Path to output corrected barcode fasta file
  -t TEMP, --tempdir TEMP
                        Path to tempdirectory
```

Bioinformatic pipeline

- Merging of the two different corrected barcode files

```
consolidate.py -h
```

```
consolidate.py -m MAFFT_aacorr_barcodes_Nfilter.fa -r RACON_aacorr_barcodes_Nfilter.fa -o
consolidated_barcodes.fa -t temp_consol
```

Now we will check which species were in our initial sample

- Navigate to the NCBI BLAST homepage

  blast.ncbi.nlm.nih.gov

- Choose nucleotide BLAST

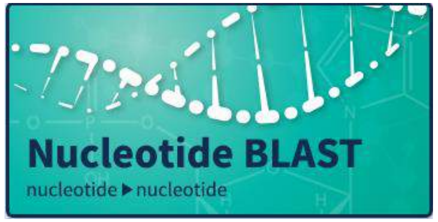

Bioinformatic pipeline

- Copy the sequences from the consolidate_barcodes.fa file into the query field either copy them from the terminal, or navigate to the file on Windows

- Scroll down and klick on BLAST

- The BLAST can take a few minutes

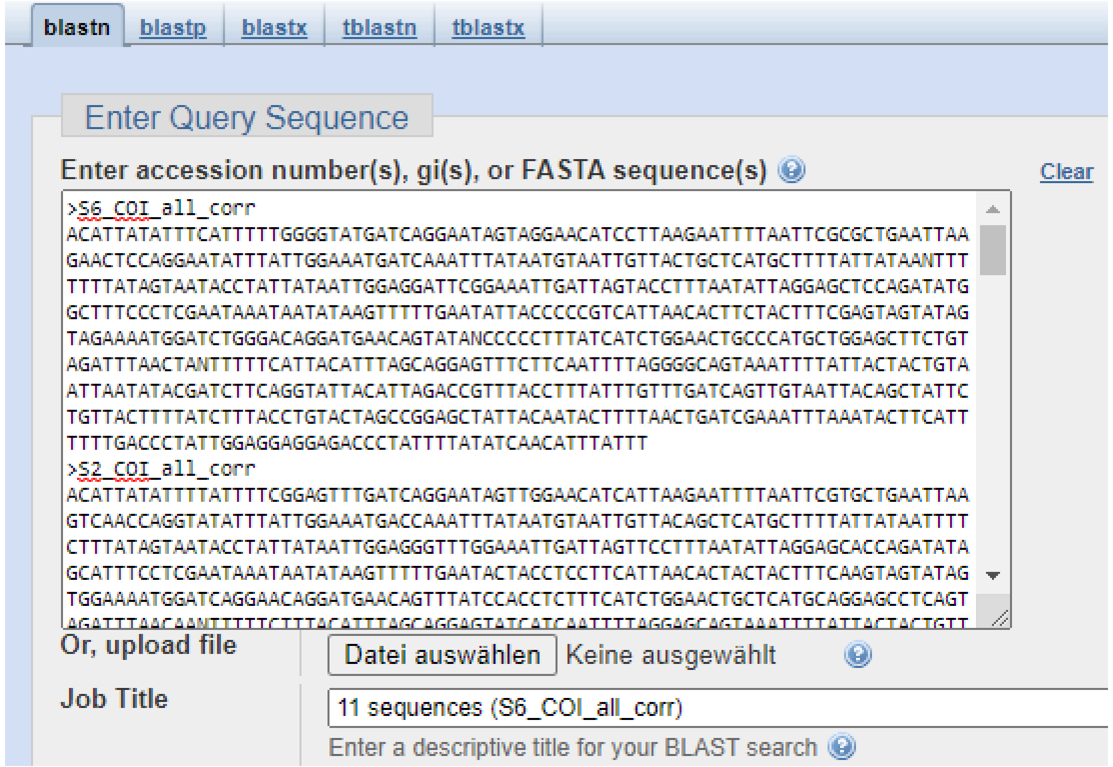

Bioinformatic pipeline

**Job Title**	**11 sequences (S6_COI_all_corr)**
**RID**	WJAEM0YY013 *Search expires on 12-05 01:09 am* Download All
**Results for**	6:lcl|Query_48906 S1_COI_all_corr(657bp)
**Program**	BLASTN Citation
**Database**	nt See details
**Query ID**	lcl|Query_48906
**Description**	S1_COI_all_corr
**Molecule type**	dna
**Query Length**	657
**Other reports**	Distance tree of results

Filter Results

Organism *only top 20 will appear* exclude

Type common name, binomial, taxid or group name

+ Add organism

Percent Identity to **E value** to **Query Coverage** to

Filter **Reset**

Descriptions Graphic Summary Alignments Taxonomy

Sequences producing significant alignments **Download** New **Select columns** Show 100

select all *100 sequences selected* GenBank Graphics Distance tree of results

	Description	Common Name	Max Score	Total Score	Query Cover	E value	Per. Ident	Acc. Len	Accession
☑	Ochlerotatus sticticus voucher APHA-4-2015G06 cytochrome oxidase subunit 1 (COI) gene, partial cds; mitochon...	Ochlerotatus sti...	1192	1192	100%	0.0	99.09%	658	MK403532.1
☑	Ochlerotatus sticticus voucher APHA-4-2015G07 cytochrome oxidase subunit 1 (COI) gene, partial cds; mitochon...	Ochlerotatus sti...	1192	1192	100%	0.0	99.09%	658	MK403176.1
☑	Ochlerotatus sticticus voucher RBINS:IG 32.776/129 cytochrome oxidase subunit 1 (COI) gene, partial cds; mitoc...	Ochlerotatus sti...	1192	1192	100%	0.0	99.09%	658	KM258266.1

Bioinformatic pipeline

ID	Morphological identification
S1_COI	Ochlerotatus sticticus
S2_COI	Ochlerotatus caspius
S3_COI	Ochlerotatus sticticus
S4_COI	Aedes vexans
S5_COI	Aedes rusticus
S6_COI	Aedes geniculatus
S7_COI	Aedes koreicus
S8_COI	Aedes japonicus
S9_COI	Aedes albopictus
S18_COI	Anopheles culicifacies
S24_COI	Anopheles tesselatus

Molecular techniques of vector identification

Primer Design

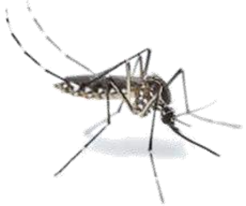

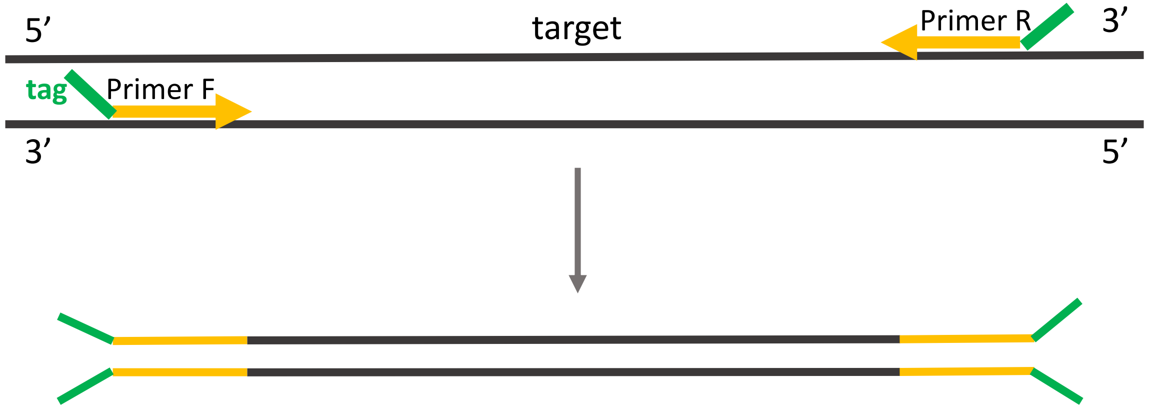

Primer Design

Example 1: double-stranded DNA sequence

```
atgcaacgatgatttttctctacaaaccacaaagatatcggtacattatattttattttaggcgcttgagct
tacgttgctactaaaaagagatgtttggtgtttctatagccatgtaatataaaataaaatccgcgaactcga

ggtatagtaggaacatctttaagtttaattatccgtgctgaactaagccaaccaggaagacttattggtaat
ccatatcatccttgtagaaattcaaattaataggcacgacttgattcggttggtccttctgaataaccatta

tgatcaaatctataatgtagtagttacagcccacgcttttgtaataattttttttatagtaatacctattac
actagtttagatattacatcatcaatgtcgggtgcgaaaacattattaaaaaaaatatcattatggataatg

taatcggaggatttggtaattgattactccctttaatattaggagctcctgatatagcattcccacgaataa
attagcctcctaaaccattaactaatgagggaaattataatcctcgaggactatatcgtaagggtgcttatt
```

Primer Design

Example 1: double-stranded DNA sequence

5' → 3'

```
5' atgcaacgatgatttttctctacaaaccacaaagatatcggtacattatattttattttaggcgcttgagct
3' tacgttgctactaaaaagagatgtttggtgtttctatagccatgtaatataaaataaaatccgcgaactcga

   ggtatagtaggaacatctttaagtttaattatccgtgctgaactaagccaaccaggaagacttattggtaat
   ccatatcatccttgtagaaattcaaattaataggcacgacttgattcggttggtccttctgaataaccatta

   tgatcaaatctataatgtagtagttacagcccacgcttttgtaataatttttttatagtaatacctattac
   actagtttagatattacatcatcaatgtcgggtgcgaaaacattattaaaaaaatatcattatggataatg

   taatcggaggatttggtaattgattactccctttaatattaggagctcctgatatagcattcccacgaataa 3'
   attagcctcctaaaccattaactaatgagggaaattataatcctcgaggactatatcgtaagggtgcttatt 5'
```

3' ← 5'

Primer Design

Example 1: double-stranded DNA sequence

5′ → 3′

```
5′ atgcaacgatgatttttctctacaaaccacaaagatatcggtacattatattttattttaggcgcttgagct
3′ tacgttgctactaaaaagagatgtttggtgtttctatagccatgtaatataaaataaaatccgcgaactcga

   ggtatagtaggaacatctttaagtttaattatccgtgctgaactaagccaaccaggaagacttattggtaat
   ccatatcatccttgtagaaattcaaattaataggcacgacttgattcggttggtccttctgaataaccatta

   tgatcaaatctataatgtagtagttacagcccacgcttttgtaataattttttttatagtaatacctattac
   actagtttagatattacatcatcaatgtcgggtgcgaaaacattattaaaaaaaatatcattatggataatg

   taatcggaggatttggtaattgattactccctttaatattaggagctcctgatatagcattcccacgaataa 3′
   attagcctcctaaaccattaactaatgagggaaattataatcctcgaggactatatcgtaagggtgcttatt 5′
```

3′ ← 5′

Primer Design

Example 1: double-stranded DNA sequence

5’ Forward primer 3’

```
5’ atgcaacgatgatttttctctacaaaccacaaagatatcggtacattatattttattttaggcgcttgagct
3’ tacgttgctactaaaaagagatgtttggtgtttctatagccatgtaatataaaataaaatccgcgaactcga

   ggtatagtaggaacatctttaagtttaattatccgtgctgaactaagccaaccaggaagacttattggtaat
   ccatatcatccttgtagaaattcaaattaataggcacgacttgattcggttggtccttctgaataaccatta

   tgatcaaatctataatgtagtagttacagcccacgcttttgtaataatttttttatagtaatacctattac
   actagtttagatattacatcatcaatgtcgggtgcgaaaacattattaaaaaaatatcattatggataatg

   taatcggaggatttggtaattgattactccctttaatattaggagctcctgatatagcattcccacgaataa 3’
   attagcctcctaaaccattaactaatgagggaaattataatcctcgaggactatatcgtaagggtgcttatt 5’
```

Reverse primer

3’ 5’

Forward Primer 5’ -> 3’: atgcaacgatgatttttctc

Reverse Primer 5’ -> 3’ : ttattcgtgggaatgctata

Primer Design

Example 2: single-stranded DNA sequence

atgcaacgatgatttttctctacaaaccacaaagatatcggtacattatattttattttaggcgcttgagct

ggtatagtaggaacatctttaagtttaattatccgtgctgaactaagccaaccaggaagacttattggtaat

tgatcaaatctataatgtagtagttacagcccacgcttttgtaataattttttttatagtaatacctattac

taatcggaggatttggtaattgattactccctttaatattaggagctcctgatatagcattcccacgaataa

Primer Design

Example 2: single-stranded DNA sequence

→

5’ atgcaacgatgatttttctctacaaaccacaaagatatcggtacattatattttattttaggcgcttgagct

ggtatagtaggaacatctttaagtttaattatccgtgctgaactaagccaaccaggaagacttattggtaat

tgatcaaatctataatgtagtagttacagcccacgcttttgtaataattttttttatagtaatacctattac

taatcggaggatttggtaattgattactccctttaatattaggagctcctgatatagcattcccacgaataa 3’

Primer Design

Example 2: single-stranded DNA sequence

5’ atgcaacgatgatttttctctacaaaccacaaagatatcggtacattatattttattttaggcgcttgagct

ggtatagtaggaacatctttaagtttaattatccgtgctgaactaagccaaccaggaagacttattggtaat

tgatcaaatctataatgtagtagttacagcccacgcttttgtaataatttttttatagtaatacctattac

taatcggaggatttggtaattgattactccctttaatattaggagctcctgatatagcattcccacgaataa 3’

Primer Design

Example 2: single-stranded DNA sequence

Forward primer

5’ atgcaacgatgatttttctctacaaaccacaaagatatcggtacattatattttattttaggcgcttgagct

ggtatagtaggaacatctttaagtttaattatccgtgctgaactaagccaaccaggaagacttattggtaat

tgatcaaatctataatgtagtagttacagcccacgcttttgtaataattttttttatagtaatacctattac

taatcggaggatttggtaattgattactccctttaatattaggagctcctgatatagcattcccacgaataa 3’

Reverse primer

Forward Primer 5’ -> 3’: atgcaacgatgatttttctc
Reverse Primer 5’ -> 3’ : ttattcgtgggaatgctata

Primer Design: Excercise

What are the sequences for forward and reverse primer?
Use 20 bases.

ccaagaatctggtaaaaaagaatccttcggtactttaggtataatttatgctatagctgcaattggtattgc

ttaggcttcgtagtttgagctcatcatatatttactgtaggtatagatgtagacactcgagcttattttata

cttccgctactataattattgctgtgcctacaggaattaaaatttttagatggttaagaactttgcatggtt

aagacaaattaatttcagcccttctctactttgggcccttggatttatttttctctttaccgtaggaggtgg

Forward Primer 5’ -> 3’:
Reverse Primer 5’ -> 3’ :

Primer Design: Excercise

What are the sequences for forward and reverse primer?
Use 20 bases.

Forward primer

5’ ccaagaatctggtaaaaaagaatccttcggtactttaggtataatttatgctatagctgcaattggtattgc

ttaggcttcgtagtttgagctcatcatatatttactgtaggtatagatgtagacactcgagcttattttata

cttccgctactataattattgctgtgcctacaggaattaaaatttttagatggttaagaactttgcatggtt

aagacaaattaatttcagcccttctctactttgggcccttggatttattttctctttaccgtaggaggtgg 3’

Reverse primer

Forward Primer 5’ -> 3’: ccaagaatctggtaaaaaag
Reverse Primer 5’ -> 3’ : ccacctcctacggtaaagag

Primer Design

Length:

- Typical length should be 18-24 bases
  - Shorter: mismatches become more likely -> more undesired products
  - Longer: annealing efficiency is lower -> PCR takes longer

GC content:

- Should be around 50-60%
- Influences the Melting temperature

Melting temperature:

- Difference in melting temperature of primer pair should not be > 5°C
- Around 55-65°C

Annealing temperature:

- Difference of Melting temperature and annealing temperature should not be >5°C

Primer-Primer interactions:

- Avoid Forward and Reverse Primer binding -> Primer dimers
- Avoid within Primer binding -> Hairpin structures

Primer Design

<u>Length</u>:

- Typical length should be 18-24 bases
    - Shorter: mismatches become more likely -> more undesired products
    - Longer: annealing efficiency is lower -> PCR takes longer

<u>GC content:</u>

- Should be around 50-60%
- Influences the Melting temperature

<u>Melting temperature:</u>

- Difference in melting temperature of primer pair should not be > 5°C
- Around 55-65°C

<u>Annealing temperature:</u>

- Difference of Melting temperature and annealing temperature should not be >5°C

Use online Primer design tools like Primer3

<u>Primer-Primer interactions:</u>

- Avoid Forward and Reverse Primer binding -> Primer dimers
- Avoid within Primer binding -> Hairpin structures

} Inactivates Primers

Primer Design

Example 2: single-stranded DNA sequence

Forward primer

5’ atgcaacgatgatttttctctacaaaccacaaagatatcggtacattatattttattttaggcgcttgagct

ggtatagtaggaacatctttaagtttaattatccgtgctgaactaagccaaccaggaagacttattggtaat

tgatcaaatctataatgtagtagttacagcccacgcttttgtaataattttttttatagtaatacctattac

taatcggaggatttggtaattgattactccctttaatattaggagctcctgatatagcattcccacgaataa 3’

Reverse primer

Forward Primer 5’ -> 3’: atgcaacgatgatttttctc
Reverse Primer 5’ -> 3’ : ttattcgtgggaatgctata

Primer Design

Example 2: single-stranded DNA sequence

Forward primer

5’ atgcaacgatgatttttctctacaaaccacaaagatatcggtacattatattttattttaggcgcttgagct

ggtatagtaggaacatctttaagtttaattatccgtgctgaactaagccaaccaggaagacttattggtaat

tgatcaaatctataatgtagtagttacagcccacgcttttgtaataatttttttatagtaatacctattac

taatcggaggatttggtaattgattactccctttaatattaggagctcctgatatagcattcccacgaataa 3’

Reverse primer

Forward Primer 5’ -> 3’: atgcaacgatgatttttctc
Reverse Primer 5’ -> 3’ : ttattcgtgggaatgctata

Where do we place our tag sequences?

gtagttattgc caggtagtaac

Primer Design

Example 2: single-stranded DNA sequence

Forward primer

5’ atgcaacgatgatttttctctacaaaccacaaagatatcggtacattatattttattttaggcgcttgagct

ggtatagtaggaacatctttaagtttaattatccgtgctgaactaagccaaccaggaagacttattggtaat

tgatcaaatctataatgtagtagttacagcccacgcttttgtaataatttttttatagtaatacctattac

taatcggaggatttggtaattgattactccctttaatattaggagctcctgatatagcattcccacgaataa 3’

Reverse primer

Forward Prgtagttattgcatgcaacgatgatttttctc
Reverse Pricaggtagtaacttattcgtgggaatgctata

Where do we place our tag sequences?

Primer Design

Example 2: single-stranded DNA sequence

Forward primer

gtagttattgc atgcaacgatgatttttctctacaaaccacaaagatatcggtacattatattttattttaggcgcttgagct

ggtatagtaggaacatctttaagtttaattatccgtgctgaactaagccaaccaggaagacttattggtaat

tgatcaaatctataatgtagtagttacagcccacgcttttgtaataattttttttatagtaatacctattac

taatcggaggatttggtaattgattactccctttaatattaggagctcctgatatagcattcccacgaataa 3’

caatgatggac

Reverse primer

Forward Prgtagttattgcatgcaacgatgatttttctc
Reverse Pricaggtagtaacttattcgtgggaatgctata

Where do we place our tag sequences?

Primer Design

ATCCGGTCGGAGA GGTCAACAAATCATAAAGATATTGG

tag — Primer

- No homopolymers >2 bp (e.g. TTT or AAA)
- Tags cannot share >6 bp sequence stretches
- Account for indels (MinION error rate!) → calculate with 3 bp errors of any kind and combination
- Cannot end in "GG"
- Length of tag is tradeoff between demultiplexing rate and PCR success
